# Computational exploration of the chemical structure space of possible reverse tricarboxylic acid cycle constituents

**DOI:** 10.1038/s41598-017-17345-7

**Published:** 2017-12-13

**Authors:** Markus Meringer, H. James Cleaves

**Affiliations:** 1 German Aerospace Center (DLR), Earth Observation Center (EOC), Münchner Straße 20, D–82234 Oberpfaffenhofen–Wessling, Germany; 2 Earth-Life Science Institute, Tokyo Institute of Technology, 2-12-IE-1 Ookayama, Meguro-ku, Tokyo, 152-8551 Japan; 3 The Institute for Advanced Study, 1 Einstein Drive, Princeton, NJ 08540 USA; 4 Blue Marble Space Institute of Science, 1515 Gallatin St. NW, Washington, DC 20011 USA; 5 Center for Chemical Evolution, Georgia Institute of Technology, Atlanta, GA 30332 Georgia

## Abstract

The reverse tricarboxylic acid (rTCA) cycle has been explored from various standpoints as an idealized primordial metabolic cycle. Its simplicity and apparent ubiquity in diverse organisms across the tree of life have been used to argue for its antiquity and its optimality. In 2000 it was proposed that chemoinformatics approaches support some of these views. Specifically, defined queries of the Beilstein database showed that the molecules of the rTCA are heavily represented in such compound databases. We explore here the chemical structure “space,” e.g. the set of organic compounds which possesses some minimal set of defining characteristics, of the rTCA cycle’s intermediates using an exhaustive structure generation method. The rTCA’s chemical space as defined by the original criteria and explored by our method is some six to seven times larger than originally considered. Acknowledging that each assumption in what is a defining criterion making the rTCA cycle special limits possible generative outcomes, there are many unrealized compounds which fulfill these criteria. That these compounds are unrealized could be due to evolutionary frozen accidents or optimization, though this optimization may also be for systems-level reasons, e.g., the way the pathway and its elements interface with other aspects of metabolism.

## Introduction

The first organisms to appear on Earth were presumably single-celled. Assuming these reproduced by binary fission as modern prokaryotes do, they would inevitably have depleted their environments of organic feedstocks rapidly due to their growth, which would have led to a very strong selective pressure for direct inorganic carbon assimilation, or carbon autotrophy. Unicellular organisms have discovered at least 6 unique pathways for accomplishing this^1–3^, including one extant linear and 5 extant cyclic pathways.

The TCA cycle is present in all known aerobic organisms, serving as an oxidative energy generating process for the cell which liberates CO_2_, as well as a process for shunting metabolites between various metabolic pools. The *r*TCA operates in several microorganisms, in which it serves as a mechanism for fixing CO_2_ into biomass. These organisms tend to cluster near the base of the universal tree of life reconstructed from 16S rRNA sequences^3^, and it has been suggested by some researchers that this was perhaps the original CO_2_ fixation process in biology, or at least oldest surviving biological CO_2_ fixation pathway^4^.

**Figure 1:**
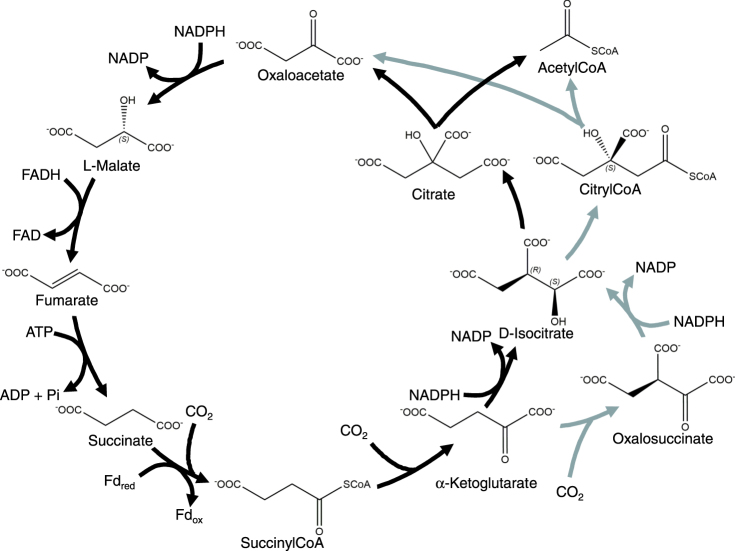
The rTCA cycle. Light arrows represent less common alternative parts of the pathway. Note that the exit of the CoA moiety during the conversion of succinylCoA to α-ketoglutarate is not explicitly represented in this figure.

It is not entirely clear which of the CO_2_ fixation pathways is the oldest, with some authors favoring the rTCA (Fig. 1) and others the Wood-Ljungdahl pathway^5–8^, and indeed, there may have been earlier carbon-fixing metabolisms which were crucial for the emergence of life but lost during evolution.

The rTCA, when run in the opposite direction, forms the core of intermediary metabolism in a wide variety of organisms. Thus 16 years ago Morowitz *et al*.^5^ documented how using a simple set of structural constraints derived from physical and chemical considerations a small set of chemical compounds, including all of the rTCA intermediates, emerged from the 3.5 million member Beilstein on-line database (now available through Reaxys, https://www.reaxys.com). That such a small number of selection rules could generate such a constrained subset of obvious biological importance was offered as evidence that this approach would prove useful in the study of biogenesis. This also was suggested to indicate that the (r)TCA metabolism, which is so central in the universal “chart of pathways”, might also be central to the origin of life, emergent from organic chemistry, and unique among possible CO_2_ fixation pathways.

These authors sought to determine the minimal set of structural rules that would return all of the compounds of the rTCA from the Beilstein database. In their analysis, and in our subsequent discussion, the contiguous backbone carbon skeletons were considered the cycle intermediates: covalently bound cofactors such as CoA were not included, nor were the various phosphoryl and electron donors and acceptors (*e.g*., ATP, NADP^+^, *etc*.) which allow the cycle to operate. Morowitz *et al*. began by looking at compounds with the formula C_*x*_H_*y*_O_*z*_ (arbitrarily excluding compounds containing N, S or P) for which 1 ≤ x ≤ 6, 1 ≤ y < 99, and 1 ≤ z < 99. 6 was chosen as the cutoff for the number of carbon atoms, as this is the minimum number to include all of the rTCA intermediates, and 99 was chosen as an arbitrary upper limit for the number of H and O atoms. This first cut yielded 2,790 compounds, including all of the rTCA cycle intermediates.

These selection rules were further refined by examining which compounds were likely to be sufficiently water soluble, and which were likely to have low heats of combustion, which gave refined selection rules: x/z ≤ 1 and y/z ≤ 2 for 1 ≤ x ≤ 3, and x/z ≤ 1 and y/z ≤ 1.5 for 4 ≤ x ≤ 6. The cutoff value of x = 6 comes from the fact that this is the largest number of C atoms found in any of the rTCA intermediates, and is thus arbitrary for this set, and not necessarily reflective of a natural restriction to cycle design.

Subsequently, compounds lacking C=O (‘carbonyl’) motifs, cyclic compounds, compounds with C—O—C (which were deemed to be difficult to synthesize non-enzymatically within this CHO formula space), compounds containing C-C triple bonds and O—O bonds (on the grounds of stability) were culled from the set. Radicals and ions were also excluded, and each pair of enantiomers was treated as a single molecule. This final set was composed of 153 compounds (see Table SI I). Henceforth we call the rules developed by Morowitz *et al*.^5^ simply the *Morowitz rules* here for the sake of brevity and to denote they are derived from that publication. There may be other combinations of rules which would more effectively achieve Morowitz *et al*.’s intended purpose, however we used the rules developed in that publication as a starting point for the sake of further exploration and comparison.

However, while this study was among the first to make use of chemoinformatics and chemical databases in an attempt to understand the logic of chemical and biochemical evolution, organic chemistry includes a much wider variety of compounds and reactions than biochemistry. While organic chemistry began as the study of biological carbon-containing compounds, it quickly grew to encompass molecules containing carbon which were accessible by non-biochemical mechanisms^9^. Though there are presently ~170,000 small molecule natural products catalogued in the Dictionary of Natural Products (http://dnp.chemnetbase.com), the “common core” of metabolism is generally considered to be composed of ~500 compounds^10^. Likewise, though the Beilstein database is one of the most extensive, organic structure space^11^ has since been explored more extensively both experimentally and computationally^12–15^, as of 2012 computed databases of possible low molecular weight compounds included up to 166 billion compounds, which is ~50,000 times larger than the Beilstein on-line database was in 2000.

There have been a few more recent approaches to the question of the relative rarity of rTCA like cycles, for example that presented by Zubarev *et al*.^16^ using reaction generation methods. This approach implemented 7 “reaction rules” iteratively applied to acetate until a product set containing 221 unique structures was generated which included all rTCA components (note that the original paper includes a typo stating that 175 unique structures were contained in this set – personal communication from author). A further study by Bar-Even *et al*. used the naturally existing reactions catalogued in the Kegg database to explore the types of new carbon-fixation pathways could potentially be engineered^17^.

For an updated and more comprehensive look at the question of how large the “rTCA-like chemical space” is, one might simply ask what the overlap of the set of structures reported by Morowitz *et al*. with compound sets retrieved from recent databases using the Morowitz rules is. However, as already suggested by Morowitz *et al*., there is an alternative, *algorithmic* approach to answering these questions: based on the Morowitz rules, one can build, using principles of graph theory and constructive combinatorics, the entire set of valid organic compounds meeting these rules. This approach has the advantage that it is complete, *i.e*. the resulting set does not grow with time and is not affected by selection bias like databases. This is the approach taken here.

Using this methodology, we find that the organic structure space of conceivable rTCA components is much larger than was considered at the time of Morowitz *et al*.’s 2000 publication. Given the multiple papers since that time which have considered biochemistry in the light of the size and physico-chemical properties of the components of any subset of chemical space^18–20^, we offer an updated evaluation of how consideration of structure space allows evaluation of the uniqueness and optimality of terrestrial intermediary metabolism.

## Results and Discussion

### Structure Enumeration

It is necessary to define a molecular model for the sake of enumeration, given that different models require different accounting techniques. Our method avoids charged and radical species, as did Morowitz *et al*.’s methodology. Furthermore, we consider tautomers to be non-unique structures, and that hydrates of carbonyl functional groups are also equilibrium structures, and thus should not be counted as unique structures. Thus we suppressed structures which contained enol motifs as well as C(OH)_2_ motifs. The logic behind this was that enols can always be resolved into other unique structures, while C(OH)_2_ motifs resolve into either aldehydes or ketones or high energy tautomers of carboxylic acids or ketenes, depending on their context.

**Figure 2:**
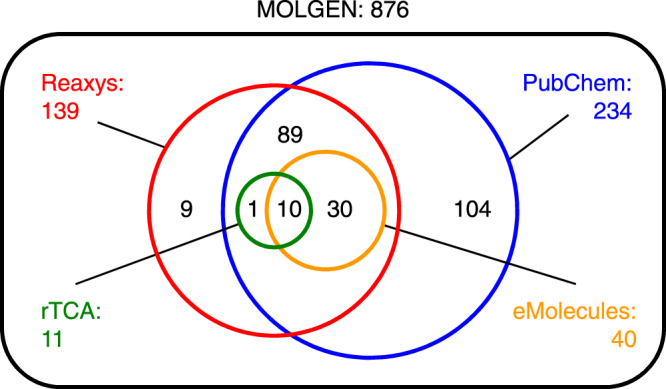
Venn diagram of compounds enumerated with MOLGEN using the Morowitz rules, their representation in online databases and the 11 intermediates of the rTCA cycle.

First we used the atomic ratio definitions of Morowitz *et al*. (2000) to define a molecular formula set. The corresponding structure space was then explored by MOLGEN 5 (see reference^21^, www.molgen.de) using the badlist items described in the Methods section and depicted in Figure SI1, which were designed to eliminate labile and tautomeric structures. This was a fairly straightforward computation, yielding a total of 876 structures (Fig. 2). Electronically readable InChI representations are provided in Table SI1.

Our generation methodology had important disparities in the representation of unique molecules compared to Morowitz *et al*. (2000). For example, this previous publication included a variety of redundant structures that were excluded by our criteria, for example the hydrates of carbonyl compounds (*e.g*., C_2_H_4_O_3_, 2, 2-di-hydroxy-acetaldehyde (CAS # 631-59-4) which our methodology returned as glyoxal, C_2_H_2_O_2_, or ethanedial (CAS # 107-22-2)) as well as enols (*e.g*., C_4_H_4_O_4_, 2-hydroxy-4-oxo-but-2-enoic acid (CAS # 114847-32-4) which our methodology returned as C_4_H_4_O_4_, 2,4-dioxobutanoic acid (CAS # 1069-50-7)).

Using these definitions, we were able to avoid a significant amount of 18,247 redundant structures in the enumerated set. However, comparison of the enols and hydrates from the eMolecules database showed that there was one structure which was identified as an enol which did not exist as a ketone in the generated set, 2,3-dihydroxyfumaric acid. This compound is found in the generated set as 2-hydroxy-3-oxobutanoic acid, and appears in both the Morowitz *et al*. and Zubarev *et al*. sets as such. Hydrates, those of formaldehyde and acetaldehyde, were found represented as such in commercial databases, and it is not unusual for some compounds to be purified as hydrates, which highlights the difficulties in making hard-and-fast exclusion rules, even for such seemingly simple compounds.

For the intersections of our enumerated set with other databases, in Reaxys there were 20 enols, 8 of which could be tautomerized two ways to give a total of 28 possible ketone parents. 17 of the 20 enols had representatives in our set, as did 23 of the 28 parent ketones. In PubMed there were 31 enols which were removed, which represented 41 possible parent tautomers. The representation of these in our set was 29/31 and 38/41 respectively. Again, the curation of molecules as their minor tautomers in databases is a problem which is difficult to completely account for.

**Figure 3:**
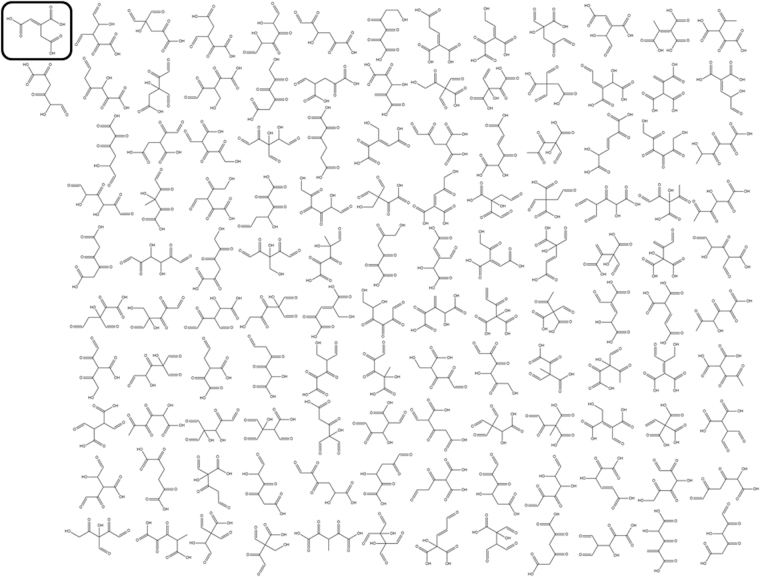
The 122 structural isomers for the formula C_6_H_6_O_6_ computed using MOLGEN and the Morowitz rules. Structures are arranged in no particular order, and are shown as their simplest extended representations (*e.g*., many could also exist as one or more hydrate, lactone, hemiacetal/ketal, anhydride, *etc*., forms). The structure corresponding to rTCA cycle intermediate *cis*-aconitic acid is highlighted by a black cartouche.

The structure space defined by the Morowitz rules is found by structure enumeration to be ~6–7 times larger than was originally considered, 876 compared to 153. However, the set found by Morowitz *et al*. is somewhat redundant by our criteria, reducing from 153 to only 119 structures upon elimination of tautomers and hydrates. The formula C_6_H_6_O_6_ (representing *cis*-aconitic acid) itself has 122 structural isomers compliant with the Morowitz rules according to our enumeration (Fig. 3).

Similarly, the structure space of Zubarev *et al*.’s computations reduces from 221 structures to 204 after removing enols, and to 165 after further removing hydrates.

Lastly, with respect to database overlap, the number of structures compound databases contain changes over time as new compounds are synthesized or discovered. We thus asked the question what would the result be if we queried the present Reaxys database with the same rules used in the 2000 study. The result was a set of 167 unique structures, which is only ~8% more than the original 153 reported.

### Stereoisomers

**Figure 4:**
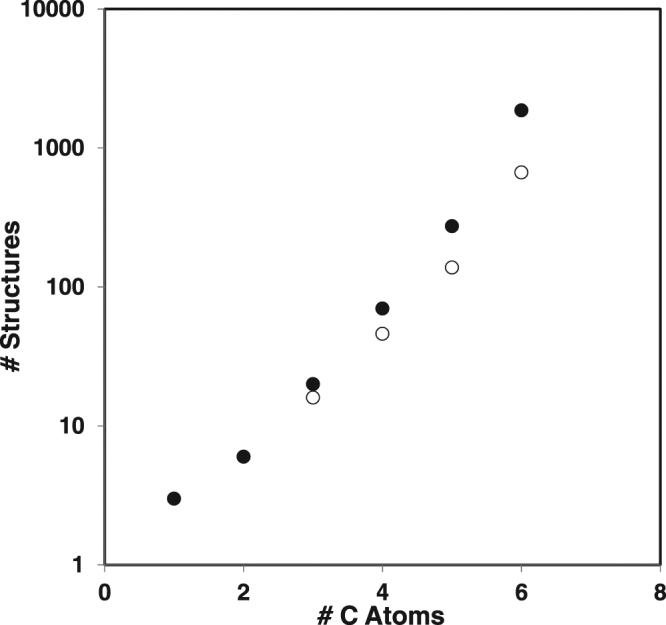
The number of structures when stereoisomerism is neglected (open circles) or taken into account (closed circles), as a function of carbon number.

The rTCA introduces a stereochemical bias to biochemistry. Three of the eleven cycle intermediates are chiral and enantiopure (isocitrate, oxalosuccinate and L-malate), and citric acid is achiral. The major feeds which directly contribute to amino acid synthesis are mostly achiral (*e.g*., pyruvate, oxaloacetate (OAA), fumarate, αKG), with the exceptions of malate, and fumarate and *cis*-aconitate which have E/Z stereoisomerism. Enumeration of the total stereoisomer space of the set of 876 compounds revealed 2236 stereoisomers, with there being approximately 3 stereoisomers per structure in this dataset (Fig. 4).

### Is the rTCA Set Unique?

In accompanying commentary, Orgel^22^ in critiquing Morowitz *et al*.’s^5^ work argued that the self-organization of the rTCA without the help of “informational” catalysts would have been a “near miracle,” and offered an alternative interpretation of their data.

The contents of the Beilstein handbook are not a selection of randomly chosen organic compounds from the set of all possible stable organic compounds up to some maximum complexity, but a collection of compounds studied by organic chemists. A compound appears in the handbook only if there was sufficient interest to justify the effort for its isolation or synthesis. Thus small natural products have a high *a priori* probability to appear in Beilstein.

Orgel suggested that Morowitz *et al*. (2000)’s pruning rules may have been devised, if only inadvertently, to allow the inclusion of these substances and exclude many other Beilstein entries. As Schuster^23^, and Orgel^22^ pointed out in accompanying commentary in the same issue, the restriction to compounds containing 6 or fewer C atoms, is an *ad hoc* assumption because the rTCA cycle contains none larger. Limiting the set to 5 carbon atoms would have excluded several rTCA intermediates, and allowing it to extend to 7 would have made the set significantly larger and made the rTCA set have seemed somewhat less special. There are both biological processes such as the Calvin-Benson cycle, and abiotic ones, such as the formose reaction^24^, that lead to larger organic compounds, which are still water soluble and have small heats of combustion. Orgel further pointed out that some of the compositional rules were somewhat arbitrary, for example the rule that for compounds with between 4 and 6 C atoms, the ratio of H/O must be ≤1.5, which excludes sugars (with general formulas C_x_H_2x_O_x_) such as ribose and glucose, while allowing trioses, which are important intermediates in several pathways in intermediary metabolism.

It should be noted that if the initial search was set to only include compounds with a maximum of 6 C atoms, the extension of the upper limit for H and O need not have been 99, as for H it could maximally have been 12, and for O maximally 2C + 1, *i.e*., 13 (if C-O-C and O-O bonding motifs are excluded; without the O-O exclusion there could be O-O-O…chains of arbitrary length). We assume the extension of these limits to 99 was due to constraints imposed by the search process at the time of the original publication.

A simple counting of possible rTCA cycle-like intermediates with respect to a finite set of isomers provides a fixed view of their relative abundance. However, as the number of possible comparison structures grows with increasing carbon number, or structural restrictions are removed, one could view the relative number of possible rTCA cycle-like intermediates as decreasing in relative uniqueness, though the actual set could be viewed as increasing in relative uniqueness. For example, the rTCA intermediates have a 7% overlap with Morowitz *et al*.’s originally enumerated set. If the redundant elements are removed from the set originally tallied by Morowitz, the representation of the biologically relevant isomers is only ~5%, and using our more extensive set of enumerated compounds which comply with the Morowitz rules, the overlap is only 1%.

However, this type of analysis belies the point that the rTCA is perhaps only one of many possible CO_2_-fixing cycles which can be constructed from such a set. For example, Zubarev *et al*.^16^ found scores of potential alternative CO_2_ fixing cycles using an iterative reaction methodology. As Schuster^23^ pointed out, the major disconnect between Morowitz *et al*.’s analysis and there being a multitude of autocatalytic sets in this structure space is the existence of suitable catalysts which make the interconversion of such sets feasible.

### Representation of Other Autotrophic Pathways in this Set

The enumerated set of 876 compounds also has significant overlap with all of the other known autotrophic carbon assimilation pathways, though complete overlap is impossible given some of the construction rules (Table 1). It is important to note that in this analysis we again exclude cofactors such as CoA and various pterins, and modifications such as phosphorylation, focusing on the carbon skeletons of these compounds.

**Table 1:** Representation of other C1 carbon fixation pathways in the enumerated set.

Pathway	rTCA Cycle	Calvin-Benson-Bassham Cycle	Wood-Ljungdahl Pathway	Dicarboxylate/4-Hydroxy butyrate Cycle	3-Hydroxy propionate Bicycle	3-Hydroxy propionate/ 4- Hydroxy butyrate Cycle
**Number of Intermediates**	11	9	4	12	13	12
**Intermediates Enumerated**	11	3	3	6	9	5
**Compounds Excluded**	None	Erythrose^1^	Methanol^1^	Enol pyruvate^3^	Propionic acid^1,4^	Acrylic acid^4^
		Ribose^1^		Succinic semialdehyde^1,4^	Methylmalic acid^1^	Propionic acid^1,4^
		Ribulose^1^		Crotonic acid^1,4^	Mesaconic acid^4^	Crotonic acid^1,4^
		Xylulose^1^		Acetoacetic acid^1,4^	Citramalic acid^1^	Acetoacetic acid^1,4^
		Fructose^1^		3-Hydroxy butyric acid^1,4^		Succinic semialdehyde^1,4^
		Sedoheptulose^1,2^		4-Hydroxy butyric acid^1,4^		4-Hydroxybutyric acid^1,4^
						4-Hydroxy butyric acid^1,4^

The reasons for their exclusion are given as superscripts in the table and annotated here: 1. H/O ratio violation; 2. C atom number violation; 3. disallowed enol tautomer; 4. C/O ratio violation.

**Figure 5:**
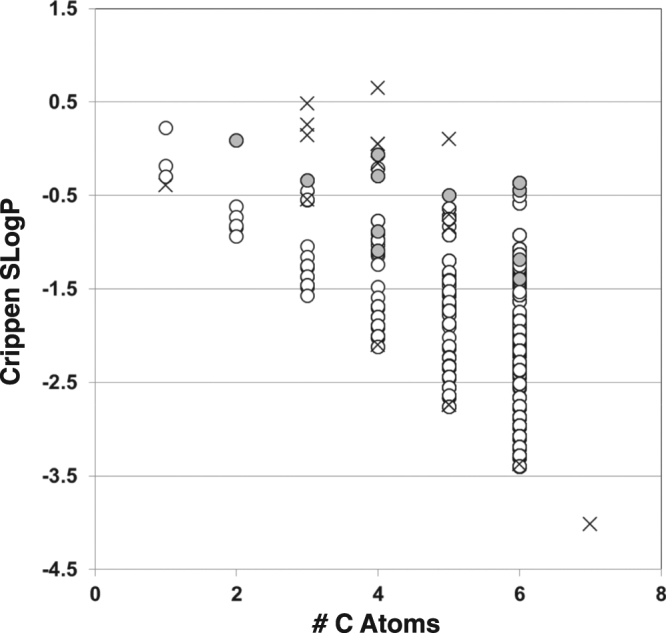
Plot of Crippen SlogP vs. carbon number for the enumerated set using the Morowitz rules (open circles), the intermediates of the rTCA (shaded circles) and the excluded intermediates of other known autotrophic CO_2_ assimilation pathways (crosses) (described in Table 1).

Morowitz *et al*.^5^ used oil-water partition coefficients to derive their rules, with the rationale that metabolic intermediates should be soluble, and unlikely to easily diffuse across cell membranes. Noting that some compounds very similar to rTCA intermediates were excluded by this enumeration, that this was due to seemingly arbitrary structural rules, and with the reasoning that one could equally start out trying to enumerate the minimum set of structure rules that would include the components of any one of these sets, we compared computed Crippen SLogP partition coefficients (see Methods section) for the enumerated set and the excluded compounds (Fig. 5).

Though a wide variety of computational solubility and partition coefficient algorithms are available, we used the Crippen SLogP calculator which comes embedded with MOLGEN-QSPR. Using other tools would likely deliver other distributions, and one could perhaps more accurately perform the calculations at a defined cellular pH value after appropriately ionizing and tautomerizing the structures. Since no methodology was provided in Morowitz *et al*.^5^ for computing partitioning, there is a significant distribution in intracellular pH values among microorganisms^25^, and it is not clear what the intracellular pH of the organism or organisms in which these pathways developed was, we performed a direct computation on the protonated compounds. However, refinements to this analysis could skew the results. As all of the compounds in these sets which could be deprotonated would then likely have more negative logP values than the SlogP rendered by this method, and selection for water solubility, which would discourage leakage of these compounds from protocells, was the stated purpose of including logP considerations in the original publication, should not have a qualitative impact on these comparisons. Figure 4 shows that the set enumerated using the Morowitz rules includes compounds exhibiting a wide range of mostly negative partition coefficient values, though the rTCA intermediates occupy a narrower range. Only four of the compounds excluded in the analysis shown in Table 1, xylulose, erythrose, ribulose and ribose, fall outside of the range of compounds allowed by the Morowitz rules. However, few if any compounds in cellular metabolism are near their solubility limits at steady state^26^, these calculations do not take into account nuances such as cyclization which can alter compounds’ partition coefficient values and propensity to cross membranes by passive diffusion^27^, and these analyses ignore the fact that many of the compounds are in fact present in cells as acyl derivatives of CoA.

### Is the rTCA Set “Optimal?”

Molecular properties are unlikely be the sole determinants of biological evolutionary optimization. “Optimality” generally connotes that something is as best as it could possibly be. How one interprets optimality in the context of biological systems, which may have multiple configurations and operational states, and which respond to environmental stresses in dynamic ways, is problematic. For some evolutionary adaptations this is fairly straightforward. For example, it is logical to expect biology to have developed methods for assimilating the most abundant forms of environmentally-available carbon, *e.g*. kinetically stable one-carbon species such as CO_2_ and CH_4_, pathways which access these sources are of obvious advantage. For more central biochemical components the reasoning becomes quickly tautological. Is life energetically optimal, materially optimal, or not optimal at all but trapped because of some other arbitrary optimality which cannot be easily over-written? A recent analysis by Noor *et al*.^28^ suggests that central metabolism is constructed in such a way that the pathways between compounds are catalytically minimal.

Finally, we consider other extant CO_2_ fixation pathways (see for example^1^). Though several CO_2_ fixation pathways are restricted to a few microbial clades, it may be arbitrary to restrict the search to the rTCA if the others might also comply with the selection rules.

The question of how autocatalytic sets can be constructed from such sets of molecules is perhaps more to the point. It is likely insufficient to consider such autocatalytic sets merely from the standpoint of their cycle characteristics (*e.g*., whether they are short or long, or what their ATP efficiency per CO_2_ molecule fixed is), because one of the principle functions of the TCA and rTCA, is the ability to shunt molecules efficiently between different compound classes, for example in moments of resource stress to interconvert sugars, amino acids and lipids^28^. The extent to which natural selection acts upon metabolic cycles which produce the molecules which are themselves the catalysts for those same cycles cannot be underestimated. For example, the shorter cycles discovered by Zubarev *et al*.^16^ which bypass OAA no longer have simple shunts into the synthesis of aspartate. If the “goal” of living organisms is to propagate themselves, then the minimal cycles which allow entry of carbon into a cycle must also allow for propagation of that system. Every cycle beyond the minimal one should be outcompeted by another one which can find a shunt through chemical space, provided the shunt does not squelch an offshoot which helps propagate the core cycle. We have recently provided evidence that, building on the work of Freeland and colleagues^29^., that not only is the genetic code optimal^30^, but also the encoded amino acids appear optimal with respect to the much larger amino acid chemical space^18^. If the amino acid set is optimal, then it can almost be considered a metabolic symbiont.

As has been pointed out, traditional methods of writing autocatalytic mechanisms in biology leads to an infinite regress problem (that there would need to be catalysts for making the intermediates which compose the catalysts), simply because it is often not recognized that the catalysts are also parts and products of such cycles^31,32^. The optimization of such cycles cannot be considered independently. Each cycle is not only nested, it is nested in a mutually dependent fashion^28^.

Biochemistry may appear optimized for various properties, but many of these are likely at levels higher than the solubility or reactivity of individual components of any given pathway. There may be higher order optimizations, which are reflected in the physical properties of the components of such systems, that are principally optimized with respect to the systems which produce them. Given the interconnected feedback nature of this sort of evolution, it may be unlikely much can conclusively be said about how these systems originated by analysis of their modern components. An important implication of this interpretation is that the use of “prebiotic chemistry” to justify life’s choice of components or the organization of metabolism may be misguided^33^.

Does the TCA cycle, running in either direction, contain an optimally minimal set of compounds? Ideally, rather than a database search, one would use the set of all possible molecules, *i.e*., with less restrictive constraints on the formula space, unbiased by whether they have ever been prepared or reported as prepared, and attempt to construct complete biochemical networks which allow for the synthesis of all cellular components. We believe this sort of analysis, while computationally daunting, is now possible and will be accomplished in the near future. It may be that there are certain catalytic steps which are especially difficult, or specific regions of enzyme “space” which were simply never explored during evolution, and some aspects of biochemical organization reflect frozen accidents.

Research to date has turned up numerous pieces of evidence of various parts of the rTCA to function non-enzymatically, both photochemically^34,35^ and/or in the presence of inorganic catalysts^36,37^. It remains unknown, however, whether the rTCA or any other cyclic or linear process derivable from the intermediates determined from a given set of rules will fully function in the absence of the catalysts or various cofactors that the modern pathways require, and there may be other CO_2_-fixing pathways which warrant experimental investigation.

## Conclusions

It is not obvious that the rTCA components are minimal, optimal or unique from the standpoint of the criteria originally argued. We suggest based on our analysis and recent work that in fact the components of the rTCA cycle are not especially unusual in the context of chemical space and there may be other higher order arguments for the structure of central metabolism.

Attempts to offer evolutionary reasons for the occurrences of various structures and pathways in biochemistry have a long history^38,39^. Given the variety of known biosynthetic mechanisms, biology often appears to use almost arbitrary sequences of transformations. Perhaps the earliest explanation that there might be an underlying chemical logic to the structure of biochemistry is the one that the pathways were derived backwards, using compounds made available from the environment^40^. This presupposed, perhaps correctly, that the evolutionary potential of enzymes was sufficient to render all necessary chemical transformations possible.

It has been suggested that the pathways developed in the forward direction, and thus that some of the extant pathways are recapitulations of geochemical processes^37,41^, in other words, that while many pathways are possible, biochemistry is largely a recapitulation of geochemistry. This has been attributed variously to the optimality of these transformations with respect to further requirements of evolvable biochemical networks, as well as frozen accident justifications^42^, the serendipitous discovery of catalytic mechanisms during evolution^43^, in addition to intrinsic bottlenecks potentially posed by catalysis before or after the origin of biology. At the molecular level, depending on how hard-wired central metabolism is, one might expect central metabolism to be more optimized relative to other more peripheral pathways.

We used graph-theory based structure enumeration techniques to explore combinatorially exhaustive the compounds which would have complied with the Morowitz rules. We found that the compound set determined in that paper is redundant: there are multiple structures which represent the same substance. This would tend to make the presence of the rTCA intermediates appear more “special” than they are by such analysis. Structure enumeration suggests the set of stable, non-redundant compounds described by the criteria used in the original study is more than 6 times larger than originally estimated, making the biological set seem somewhat more “special.” It can be argued this is an inevitable outcome of this sort of study. The enumerated set also includes a large proportion of intermediates of other CO_2_ assimilation pathways.

The number of compounds present in chemical databases has grown considerably since the original analysis was published, which also contributes to the perception of how “special” this set seems. While it is surprising that two thirds of the compounds found by structure enumeration have not been reported in chemical databases, we do not believe this is entirely due to the difficulty of their synthesis or their instability, but is also partially due to lack of motivation for their synthesis, and due to the fact that these were not encountered in chemical analysis of living matter.

Among the numerous known C1 assimilation pathways, the rTCA cycle is widely distributed among some of the most deeply branching organisms^44–47^. However, it has now been pointed out there may be a great number of potentially realizable autocatalytic cycles, some of which may involve more “prebiotically plausible” or simply different components. An example of one of these which has received considerable attention is Eschenmoser’s so-called “glyoxylate cycle”^48–51^ which could convert HCN, long-believed to be a key synthon for prebiotic synthesis, into biosynthetic intermediates via mechanisms distinct from those of modern biochemistry. Such cycles would inherently be excluded from the present analysis which excludes N-containing compounds. While there has been some recent progress in experimentally reconstituting aspects of the rTCA cycle under geochemically plausible conditions^36,37^, there may be many others left to be explored.

Considerations to date of the sizes of chemical space with respect to what biology has had to explore based merely on chemical structures lead to two conclusions: 1. It could perhaps have been otherwise and 2. It appears that there are higher order selection rules which have not been considered, *i.e*., no aspect of modern biochemistry can be considered without considering the potential impacts on other aspects of the system. Structure enumeration methods are close to exhausting small molecule structure space (less than or equal to ~150 amu), though it is very large, and the degree to which one or another set of compounds can be considered “optimal” depends on assuming the existence of catalysts derived by unspecified means during evolution. The structures of amino acids derived from central metabolism which compose the enzyme catalysts which themselves mediate that metabolism suggest that amino acid biosynthesis would have been an early selection pressure in constructing the metabolic pathways. Once mechanisms existed for making amino acids, they would have strongly influenced the course of metabolic evolution.

The aromatic amino acids and histidine derive from biochemical pathways which do not directly branch from the rTCA cycle, and the presence of these molecules in the enzymes which catalyze the contemporary cycle could be interpreted as providing evidence for the operation of metabolic pathways which allowed for their synthesis in the organism in which the rTCA developed^52^.

Given the complexities of inorganic nitrogen assimilation, and the fact that N can be introduced into many organic compounds via a very small number of enzymatic steps (*e.g*., reductive amination and transamination), we think it is reasonable that the components of intermediate metabolism should be composed of only C, H and O. Whether S or P are also required is debatable, certainly the question of whether the modern pathways emerged from an “RNA”^53^ or “Thioester”^54^ world has some bearing on this discussion^55^. Regardless of these possibilities, an exploration of a more comprehensive structure space, vetted by an *in silico* reaction exploration can be used to great effect in exploring the origin of the metabolic pathways. We are currently in the process of exploring this set construction using these methods.

## Methods

### Set Construction

We used MOLGEN 5^21^ to explore the total chemical space defined by the Morowitz rules. MOLGEN applies the principle of orderly generation^56–58^ to molecular graphs^59,60^, which are graphs representing structural formulas where vertices correspond to atoms and edges represent covalent bonds. Orderly generation is a widely used methodology for exhaustive and non-redundant enumeration of discrete structures, a molecular graph is a mathematical representation of a chemical compound on the topological level (i.e. without stereo information) as a discrete structure. For more information on orderly generation applied to molecular graphs see also references^61–64^, for an open source implementation see^65^.

MOLGEN is able to account for various types of constraints during the structure generation process. The Morowitz rules can be grouped into two categories, concerning the compositional and the structural level.

Rules on the compositional level are used to define the molecular formulas to be considered. For a general formula C_x_H_y_O_z_ the numbers of atoms per element are limited to be 1 ≤ x ≤ 6, 1 ≤ y ≤ 99 and 1 ≤ z ≤ 99. Ratios of atoms are restricted as follows: x/z ≤ 1 and y/z ≤ 2 for 1 ≤ x ≤ 3, and x/z ≤ 1 and y/z ≤ 1.5 for 4 ≤ x ≤ 6. Transformed into equivalent expressions z-x ≥ 0 and 2z-y ≥ 0 for 1 ≤ x ≤ 3, and z-x ≥ 0 and 3z-2y ≥ 0, these can directly be entered to MOLGEN. These rules obviously define a finite set of molecular formulas that can be enumerated exhaustively by combinatorial means, e.g. by using a simple backtracking algorithm as described in reference^63^. Such an algorithm is also implemented in MOLGEN 5.

For speeding up computations we narrowed down the number of oxygen atoms to be at most 2x + 1. This tighter boundary can be derived using the structural constraints as follows: Absence of C-O-C and O-O limits the number of O atoms to a maximum of 2x + 2. Keeping in mind that there must be a carbonyl group present reduces the number of O atoms by one, resulting y ≤ 2x + 1.

Rules on the structural level can be input to MOLGEN as so-called goodlist and badlist items. Beyond C-O-C and O-O we added C=C=A to this badlist (where A denotes any non-hydrogen atom), as well as two variations of OH-C-OH (A_2_C(OH)_2_ and ACH(OH)_2_) in order to exclude hydrates of carbonyls (for example ACH(OH)_2_ excludes CH_3_CH(OH)_2_, which is the hydrate of acetaldehyde CH_3_CHO). Since these compounds exist in equilibrium in aqueous solution, they are not considered unique compounds for our purposes. Maximal bond multiplicity of two was used to exclude C≡C, and cyclic compounds were excluded by setting the number of cycles to zero for MOLGEN’s input. We further excluded enols. Though keto-enol tautomerism equilibria could be near unity under some conditions, we considered tautomers here to be non-unique compounds, and favored the keto-forms over the enol forms.

Again these rules define a finite set of molecular graphs that can be completely enumerated by orderly generation. The correctness of the principle of orderly generation is mathematically proven in the original publications^56–58^. The correctness of MOLGEN 5 is tested by comparison with preceding versions MOLGEN 4^66^ and MOLGEN 3.5^67,68^, which both had different code bases. Agreement in results with these preceding versions on a large number of test cases that cover all features of the structure generator provides a high level of confidence that the algorithms are implemented free of errors. The working principle of MOLGEN 5 and the exact commands used for operating MOLGEN 5 are described in the SI.

### Stereoisomer Generation

Though Morowitz *et al*. (2000) excluded stereoisomers from their tally of compounds, we thought it of interest to record the number of stereoisomers per molecular and constitutional formula. Stereoisomerism contributes to the chemical complexity of the compounds involved in a cycle and the amount of chemical information which must be conserved or transformed to allow a cycle to operate efficiently, *e.g*., a different and likely higher degree of substrate specificity is required to facilitate stereospecific reactions. We defined the entire stereochemical space of this set using Instant JChem’s command line cxcalc function (Calculator Plugins were used for structure property prediction and calculation, Marvin 15.10.26.0, 2015, provided by ChemAxon (www.chemaxon.com)). To do so, the MOLGEN output was written in SD file format, which was then processed by cxcalc to generate stereoisomers.

### Database Searching

Finally, we explored the degree to which the compounds defined by these composition rules and generated by our methods have been reported in the literature, as a metric of the degree to which their real-world chemistry has been explored. To do so, we converted the set to InChi format and searched the Reaxys database (www.reaxys.com) in batch search mode. Reaxys is essentially the up-to-date version of the former Beilstein online database; it also cross-references the PubMed and eMolecules databases.

The databases differ in important aspects, for example, Reaxys comprises a large set of peer-reviewed collated and curated structures, which are redundant in some respects, while PubMed is publicly curated and eMolecules represents only compounds which are commercially available (though there may be commercial sources of compounds not indexed by this database, and there may be compounds listed which are synthesized on demand, thus stocks of these compounds are not maintained, and in some cases there may be no literature precedent for their synthesis). To some extent, the presence of a compound in the eMolecules database represents its stability (as it can be prepared, stored and sold) and synthetic accessibility, it also represents the commercial and research utility of a given compound.

### Descriptor Computation

For estimating the octanol/water partitioning coefficient, a proxy for solubility in water, we used Crippen SLogP values^69^ computed by MOLGEN-QSPR^70^.

## Electronic supplementary material

Supplementary Information
