# Supplementary material for: Computational exploration of the chemical structure space of possible reverse tricarboxylic acid cycle constituents
Source: Sci Rep. 2017 Dec 13;7:17540. doi: 10.1038/s41598-017-17345-7 (PMC5727506; doi:10.1038/s41598-017-17345-7)
Supplement: Supplementary file 1 — Supplementary Information [file 41598_2017_17345_MOESM1_ESM.docx]

**Supplementary Information for Computational Exploration of the Chemical Structure Space of Possible Reverse Tricarboxylic Acid Cycle Constituents**

**Markus Meringer & H. James Cleaves II**

|  | Structure | Morowitz | Zubaev | MOLGEN | eMolecules | PubChem | Reaxys | rTCA |
| --- | --- | --- | --- | --- | --- | --- | --- | --- |
| 1 | InChI=1S/CH2O/c1-2/h1H2 | 1 | 1 | 1 | 1 | 1 | 1 | 0 |
| 2 | InChI=1S/CH2O2/c2-1-3/h1H,(H,2,3) | 1 | 1 | 1 | 1 | 1 | 1 | 0 |
| 3 | InChI=1S/CH2O3/c2-1(3)4/h(H2,2,3,4) | 0 | 0 | 1 | 1 | 1 | 1 | 0 |
| 4 | InChI=1S/C2H2O2/c3-1-2-4/h1-2H | 1 | 1 | 1 | 1 | 1 | 1 | 0 |
| 5 | InChI=1S/C2H2O3/c3-1-2(4)5/h1H,(H,4,5) | 1 | 1 | 1 | 1 | 1 | 1 | 0 |
| 6 | InChI=1S/C2H2O4/c3-1(4)2(5)6/h(H,3,4)(H,5,6) | 1 | 1 | 1 | 1 | 1 | 1 | 0 |
| 7 | InChI=1S/C2H4O2/c3-1-2-4/h1,4H,2H2 | 1 | 1 | 1 | 1 | 1 | 1 | 0 |
| 8 | InChI=1S/C2H4O2/c1-2(3)4/h1H3,(H,3,4) | 1 | 1 | 1 | 1 | 1 | 1 | 1 |
| 9 | InChI=1S/C2H4O3/c3-1-2(4)5/h3H,1H2,(H,4,5) | 1 | 1 | 1 | 1 | 1 | 1 | 0 |
| 10 | InChI=1S/C3H2O3/c4-1-3(6)2-5/h1-2H | 1 | 0 | 1 | 0 | 1 | 1 | 0 |
| 11 | InChI=1S/C3H2O4/c4-1-2(5)3(6)7/h1H,(H,6,7) | 1 | 1 | 1 | 0 | 1 | 1 | 0 |
| 12 | InChI=1S/C3H2O5/c4-1(2(5)6)3(7)8/h(H,5,6)(H,7,8) | 1 | 1 | 1 | 1 | 1 | 1 | 0 |
| 13 | InChI=1S/C3H4O3/c4-1-3(6)2-5/h1-3,6H | 1 | 0 | 1 | 1 | 1 | 1 | 0 |
| 14 | InChI=1S/C3H4O3/c4-1-3(6)2-5/h1,5H,2H2 | 1 | 0 | 1 | 1 | 1 | 1 | 0 |
| 15 | InChI=1S/C3H4O3/c4-2-1-3(5)6/h2H,1H2,(H,5,6) | 1 | 1 | 1 | 0 | 1 | 1 | 0 |
| 16 | InChI=1S/C3H4O3/c1-2(4)3(5)6/h1H3,(H,5,6) | 1 | 1 | 1 | 1 | 1 | 1 | 1 |
| 17 | InChI=1S/C3H4O4/c4-1-2(5)3(6)7/h1-2,5H,(H,6,7) | 1 | 1 | 1 | 0 | 1 | 1 | 0 |
| 18 | InChI=1S/C3H4O4/c4-1-2(5)3(6)7/h4H,1H2,(H,6,7) | 1 | 1 | 1 | 0 | 1 | 1 | 0 |
| 19 | InChI=1S/C3H4O4/c4-2(5)1-3(6)7/h1H2,(H,4,5)(H,6,7) | 1 | 1 | 1 | 1 | 1 | 1 | 0 |
| 20 | InChI=1S/C3H4O5/c4-1(2(5)6)3(7)8/h1,4H,(H,5,6)(H,7,8) | 1 | 1 | 1 | 1 | 1 | 1 | 0 |
| 21 | InChI=1S/C3H6O3/c4-1-3(6)2-5/h1,3,5-6H,2H2 | 1 | 0 | 1 | 1 | 1 | 1 | 0 |
| 22 | InChI=1S/C3H6O3/c1-2(4)3(5)6/h2,4H,1H3,(H,5,6) | 1 | 1 | 1 | 1 | 1 | 1 | 0 |
| 23 | InChI=1S/C3H6O3/c4-1-3(6)2-5/h4-5H,1-2H2 | 1 | 0 | 1 | 1 | 1 | 1 | 0 |
| 24 | InChI=1S/C3H6O3/c4-2-1-3(5)6/h4H,1-2H2,(H,5,6) | 1 | 1 | 1 | 1 | 1 | 1 | 0 |
| 25 | InChI=1S/C3H6O4/c4-1-2(5)3(6)7/h2,4-5H,1H2,(H,6,7) | 1 | 1 | 1 | 1 | 1 | 1 | 0 |
| 26 | InChI=1S/C4H2O4/c5-1-3(7)4(8)2-6/h1-2H | 1 | 0 | 1 | 0 | 1 | 1 | 0 |
| 27 | InChI=1S/C4H2O5/c5-1-2(6)3(7)4(8)9/h1H,(H,8,9) | 0 | 0 | 1 | 0 | 0 | 0 | 0 |
| 28 | InChI=1S/C4H2O6/c5-1(3(7)8)2(6)4(9)10/h(H,7,8)(H,9,10) | 1 | 1 | 1 | 1 | 1 | 1 | 0 |
| 29 | InChI=1S/C4H4O4/c5-3(6)1-2-4(7)8/h1-2H,(H,5,6)(H,7,8) | 1 | 1 | 1 | 1 | 1 | 1 | 1 |
| 30 | InChI=1S/C4H4O4/c1-2(3(5)6)4(7)8/h1H2,(H,5,6)(H,7,8) | 1 | 1 | 1 | 0 | 1 | 1 | 0 |
| 31 | InChI=1S/C4H4O4/c1-2(5)3(6)4(7)8/h1H3,(H,7,8) | 0 | 1 | 1 | 0 | 1 | 1 | 0 |
| 32 | InChI=1S/C4H4O4/c5-1-3(7)4(8)2-6/h1-3,7H | 0 | 0 | 1 | 0 | 1 | 0 | 0 |
| 33 | InChI=1S/C4H4O4/c5-1-3(2-6)4(7)8/h1-3H,(H,7,8) | 0 | 0 | 1 | 0 | 1 | 0 | 0 |
| 34 | InChI=1S/C4H4O4/c5-1-4(8,2-6)3-7/h1-3,8H | 0 | 0 | 1 | 0 | 0 | 0 | 0 |
| 35 | InChI=1S/C4H4O4/c5-1-3(7)4(8)2-6/h1,6H,2H2 | 0 | 0 | 1 | 0 | 1 | 0 | 0 |
| 36 | InChI=1S/C4H4O4/c5-2-1-3(6)4(7)8/h2H,1H2,(H,7,8) | 1 | 1 | 1 | 0 | 1 | 1 | 0 |
| 37 | InChI=1S/C4H4O4/c5-2-3(6)1-4(7)8/h2H,1H2,(H,7,8) | 1 | 0 | 1 | 0 | 1 | 1 | 0 |
| 38 | InChI=1S/C4H4O5/c5-1-2(6)3(7)4(8)9/h5H,1H2,(H,8,9) | 0 | 0 | 1 | 0 | 0 | 0 | 0 |
| 39 | InChI=1S/C4H4O5/c5-2(4(8)9)1-3(6)7/h1H2,(H,6,7)(H,8,9) | 1 | 1 | 1 | 1 | 1 | 1 | 1 |
| 40 | InChI=1S/C4H4O5/c5-1-2(6)3(7)4(8)9/h1-2,6H,(H,8,9) | 0 | 0 | 1 | 0 | 0 | 0 | 0 |
| 41 | InChI=1S/C4H4O5/c5-1-2(6)3(7)4(8)9/h1,3,7H,(H,8,9) | 0 | 0 | 1 | 0 | 0 | 0 | 0 |
| 42 | InChI=1S/C4H4O5/c5-1-2(3(6)7)4(8)9/h1-2H,(H,6,7)(H,8,9) | 0 | 0 | 1 | 0 | 1 | 1 | 0 |
| 43 | InChI=1S/C4H4O5/c5-1-4(9,2-6)3(7)8/h1-2,9H,(H,7,8) | 0 | 0 | 1 | 0 | 0 | 0 | 0 |
| 44 | InChI=1S/C4H4O6/c5-1-4(10,2(6)7)3(8)9/h1,10H,(H,6,7)(H,8,9) | 0 | 0 | 1 | 0 | 0 | 0 | 0 |
| 45 | InChI=1S/C4H4O6/c5-1(3(7)8)2(6)4(9)10/h1,5H,(H,7,8)(H,9,10) | 1 | 1 | 1 | 0 | 1 | 1 | 0 |
| 46 | InChI=1S/C4H4O6/c5-2(6)1(3(7)8)4(9)10/h1H,(H,5,6)(H,7,8)(H,9,10) | 0 | 1 | 1 | 0 | 1 | 0 | 0 |
| 47 | InChI=1S/C4H4O7/c5-1(6)4(11,2(7)8)3(9)10/h11H,(H,5,6)(H,7,8)(H,9,10) | 1 | 1 | 1 | 0 | 1 | 1 | 0 |
| 48 | InChI=1S/C4H6O4/c5-1-3(7)4(8)2-6/h1,4,6,8H,2H2 | 1 | 0 | 1 | 0 | 1 | 1 | 0 |
| 49 | InChI=1S/C4H6O4/c5-1-3(7)4(8)2-6/h1,3,6-7H,2H2 | 0 | 0 | 1 | 0 | 1 | 0 | 0 |
| 50 | InChI=1S/C4H6O4/c5-1-3(2-6)4(7)8/h1,3,6H,2H2,(H,7,8) | 0 | 0 | 1 | 0 | 1 | 0 | 0 |
| 51 | InChI=1S/C4H6O4/c5-2-1-3(6)4(7)8/h2-3,6H,1H2,(H,7,8) | 1 | 1 | 1 | 0 | 1 | 1 | 0 |
| 52 | InChI=1S/C4H6O4/c5-2-3(6)1-4(7)8/h2-3,6H,1H2,(H,7,8) | 1 | 1 | 1 | 0 | 1 | 1 | 0 |
| 53 | InChI=1S/C4H6O4/c5-1-3(7)4(8)2-6/h1-4,7-8H | 1 | 0 | 1 | 0 | 1 | 1 | 0 |
| 54 | InChI=1S/C4H6O4/c1-2(5)3(6)4(7)8/h2,5H,1H3,(H,7,8) | 1 | 1 | 1 | 0 | 1 | 1 | 0 |
| 55 | InChI=1S/C4H6O4/c1-2(5)3(6)4(7)8/h3,6H,1H3,(H,7,8) | 1 | 1 | 1 | 0 | 1 | 1 | 0 |
| 56 | InChI=1S/C4H6O4/c1-2(3(5)6)4(7)8/h2H,1H3,(H,5,6)(H,7,8) | 1 | 1 | 1 | 1 | 1 | 1 | 0 |
| 57 | InChI=1S/C4H6O4/c5-1-4(8,2-6)3-7/h1-2,7-8H,3H2 | 0 | 0 | 1 | 0 | 0 | 0 | 0 |
| 58 | InChI=1S/C4H6O4/c1-4(8,2-5)3(6)7/h2,8H,1H3,(H,6,7) | 0 | 0 | 1 | 0 | 1 | 0 | 0 |
| 59 | InChI=1S/C4H6O4/c5-1-3(7)4(8)2-6/h5-6H,1-2H2 | 1 | 0 | 1 | 0 | 1 | 1 | 0 |
| 60 | InChI=1S/C4H6O4/c5-2-1-3(6)4(7)8/h5H,1-2H2,(H,7,8) | 1 | 1 | 1 | 0 | 1 | 1 | 0 |
| 61 | InChI=1S/C4H6O4/c5-2-3(6)1-4(7)8/h5H,1-2H2,(H,7,8) | 0 | 0 | 1 | 0 | 1 | 0 | 0 |
| 62 | InChI=1S/C4H6O4/c5-3(6)1-2-4(7)8/h1-2H2,(H,5,6)(H,7,8) | 1 | 1 | 1 | 1 | 1 | 1 | 1 |
| 63 | InChI=1S/C4H6O5/c5-1-2(6)3(7)4(8)9/h1-3,6-7H,(H,8,9) | 1 | 0 | 1 | 0 | 1 | 1 | 0 |
| 64 | InChI=1S/C4H6O5/c5-1-2(6)3(7)4(8)9/h2,5-6H,1H2,(H,8,9) | 1 | 0 | 1 | 0 | 1 | 1 | 0 |
| 65 | InChI=1S/C4H6O5/c5-1-2(6)3(7)4(8)9/h3,5,7H,1H2,(H,8,9) | 0 | 0 | 1 | 0 | 1 | 0 | 0 |
| 66 | InChI=1S/C4H6O5/c5-1-2(3(6)7)4(8)9/h2,5H,1H2,(H,6,7)(H,8,9) | 1 | 1 | 1 | 0 | 1 | 1 | 0 |
| 67 | InChI=1S/C4H6O5/c5-2(4(8)9)1-3(6)7/h2,5H,1H2,(H,6,7)(H,8,9) | 1 | 1 | 1 | 1 | 1 | 1 | 1 |
| 68 | InChI=1S/C4H6O5/c5-1-4(9,2-6)3(7)8/h1,6,9H,2H2,(H,7,8) | 0 | 0 | 1 | 0 | 1 | 0 | 0 |
| 69 | InChI=1S/C4H6O5/c1-4(9,2(5)6)3(7)8/h9H,1H3,(H,5,6)(H,7,8) | 1 | 1 | 1 | 0 | 1 | 1 | 0 |
| 70 | InChI=1S/C4H6O6/c5-1-4(10,2(6)7)3(8)9/h5,10H,1H2,(H,6,7)(H,8,9) | 1 | 0 | 1 | 0 | 1 | 1 | 0 |
| 71 | InChI=1S/C4H6O6/c5-1(3(7)8)2(6)4(9)10/h1-2,5-6H,(H,7,8)(H,9,10) | 1 | 1 | 1 | 1 | 1 | 1 | 0 |
| 72 | InChI=1S/C5H2O5/c6-1-3(8)5(10)4(9)2-7/h1-2H | 1 | 0 | 1 | 0 | 0 | 0 | 0 |
| 73 | InChI=1S/C5H2O6/c6-1-2(7)3(8)4(9)5(10)11/h1H,(H,10,11) | 0 | 0 | 1 | 0 | 0 | 0 | 0 |
| 74 | InChI=1S/C5H2O7/c6-1(2(7)4(9)10)3(8)5(11)12/h(H,9,10)(H,11,12) | 0 | 0 | 1 | 0 | 0 | 0 | 0 |
| 75 | InChI=1S/C5H4O5/c6-2-1-3(4(7)8)5(9)10/h1-2H,(H,7,8)(H,9,10) | 0 | 0 | 1 | 0 | 0 | 0 | 0 |
| 76 | InChI=1S/C5H4O5/c6-2-3(5(9)10)1-4(7)8/h1-2H,(H,7,8)(H,9,10) | 1 | 1 | 1 | 0 | 1 | 1 | 0 |
| 77 | InChI=1S/C5H4O5/c6-3(5(9)10)1-2-4(7)8/h1-2H,(H,7,8)(H,9,10) | 1 | 0 | 1 | 0 | 1 | 1 | 0 |
| 78 | InChI=1S/C5H4O5/c1-2(4(7)8)3(6)5(9)10/h1H2,(H,7,8)(H,9,10) | 0 | 1 | 1 | 0 | 0 | 0 | 0 |
| 79 | InChI=1S/C5H4O5/c1-2(6)3(7)4(8)5(9)10/h1H3,(H,9,10) | 0 | 0 | 1 | 0 | 0 | 0 | 0 |
| 80 | InChI=1S/C5H4O5/c6-1-3(8)5(10)4(9)2-7/h1-3,8H | 0 | 0 | 1 | 0 | 0 | 0 | 0 |
| 81 | InChI=1S/C5H4O5/c6-1-3(2-7)4(8)5(9)10/h1-3H,(H,9,10) | 0 | 0 | 1 | 0 | 0 | 0 | 0 |
| 82 | InChI=1S/C5H4O5/c6-1-3(8)5(10)4(9)2-7/h1-2,5,10H | 0 | 0 | 1 | 0 | 0 | 0 | 0 |
| 83 | InChI=1S/C5H4O5/c6-1-3(5(9)10)4(8)2-7/h1-3H,(H,9,10) | 0 | 0 | 1 | 0 | 0 | 0 | 0 |
| 84 | InChI=1S/C5H4O5/c6-1-3(8)5(10)4(9)2-7/h1,7H,2H2 | 0 | 0 | 1 | 0 | 0 | 0 | 0 |
| 85 | InChI=1S/C5H4O5/c6-2-1-3(7)4(8)5(9)10/h2H,1H2,(H,9,10) | 0 | 0 | 1 | 0 | 0 | 0 | 0 |
| 86 | InChI=1S/C5H4O5/c6-2-4(8)3(7)1-5(9)10/h2H,1H2,(H,9,10) | 0 | 0 | 1 | 0 | 0 | 0 | 0 |
| 87 | InChI=1S/C5H4O5/c6-2-3(7)1-4(8)5(9)10/h2H,1H2,(H,9,10) | 0 | 0 | 1 | 0 | 0 | 0 | 0 |
| 88 | InChI=1S/C5H4O5/c6-1-4(9)5(10,2-7)3-8/h1-3,10H | 0 | 0 | 1 | 0 | 0 | 0 | 0 |
| 89 | InChI=1S/C5H4O5/c6-1-5(2-7,3-8)4(9)10/h1-3H,(H,9,10) | 0 | 0 | 1 | 0 | 0 | 0 | 0 |
| 90 | InChI=1S/C5H4O6/c6-3(7)1-2(4(8)9)5(10)11/h1H,(H,6,7)(H,8,9)(H,10,11) | 1 | 0 | 1 | 0 | 1 | 1 | 0 |
| 91 | InChI=1S/C5H4O6/c6-1-2(7)3(8)4(9)5(10)11/h6H,1H2,(H,10,11) | 0 | 0 | 1 | 0 | 0 | 0 | 0 |
| 92 | InChI=1S/C5H4O6/c6-2(1-3(7)8)4(9)5(10)11/h1H2,(H,7,8)(H,10,11) | 0 | 0 | 1 | 0 | 0 | 0 | 0 |
| 93 | InChI=1S/C5H4O6/c6-2(4(8)9)1-3(7)5(10)11/h1H2,(H,8,9)(H,10,11) | 0 | 1 | 1 | 0 | 0 | 0 | 0 |
| 94 | InChI=1S/C5H4O6/c6-1-2(7)3(8)4(9)5(10)11/h1-2,7H,(H,10,11) | 0 | 0 | 1 | 0 | 0 | 0 | 0 |
| 95 | InChI=1S/C5H4O6/c6-1-2(7)3(8)4(9)5(10)11/h1,4,9H,(H,10,11) | 0 | 0 | 1 | 0 | 0 | 0 | 0 |
| 96 | InChI=1S/C5H4O6/c6-1-2(7)3(8)4(9)5(10)11/h1,3,8H,(H,10,11) | 0 | 0 | 1 | 0 | 0 | 0 | 0 |
| 97 | InChI=1S/C5H4O6/c6-1-2(4(8)9)3(7)5(10)11/h1-2H,(H,8,9)(H,10,11) | 0 | 0 | 1 | 0 | 0 | 0 | 0 |
| 98 | InChI=1S/C5H4O6/c6-1-2(7)3(4(8)9)5(10)11/h1,3H,(H,8,9)(H,10,11) | 0 | 0 | 1 | 0 | 0 | 0 | 0 |
| 99 | InChI=1S/C5H4O6/c6-1-5(11,2-7)3(8)4(9)10/h1-2,11H,(H,9,10) | 0 | 0 | 1 | 0 | 0 | 0 | 0 |
| 100 | InChI=1S/C5H4O6/c6-1-3(8)5(11,2-7)4(9)10/h1-2,11H,(H,9,10) | 0 | 0 | 1 | 0 | 0 | 0 | 0 |
| 101 | InChI=1S/C5H4O6/c6-1-5(2-7,3(8)9)4(10)11/h1-2H,(H,8,9)(H,10,11) | 0 | 0 | 1 | 0 | 0 | 0 | 0 |
| 102 | InChI=1S/C5H4O7/c6-1-5(12,4(10)11)2(7)3(8)9/h1,12H,(H,8,9)(H,10,11) | 0 | 0 | 1 | 0 | 0 | 0 | 0 |
| 103 | InChI=1S/C5H4O7/c6-1-2(7)5(12,3(8)9)4(10)11/h1,12H,(H,8,9)(H,10,11) | 0 | 0 | 1 | 0 | 0 | 0 | 0 |
| 104 | InChI=1S/C5H4O7/c6-1-5(2(7)8,3(9)10)4(11)12/h1H,(H,7,8)(H,9,10)(H,11,12) | 0 | 0 | 1 | 0 | 0 | 0 | 0 |
| 105 | InChI=1S/C5H4O7/c6-1(2(7)4(9)10)3(8)5(11)12/h2,7H,(H,9,10)(H,11,12) | 1 | 0 | 1 | 0 | 1 | 1 | 0 |
| 106 | InChI=1S/C5H4O7/c6-1(2(7)4(9)10)3(8)5(11)12/h1,6H,(H,9,10)(H,11,12) | 0 | 0 | 1 | 0 | 0 | 0 | 0 |
| 107 | InChI=1S/C5H4O7/c6-2(5(11)12)1(3(7)8)4(9)10/h1H,(H,7,8)(H,9,10)(H,11,12) | 1 | 0 | 1 | 0 | 1 | 1 | 0 |
| 108 | InChI=1S/C5H4O8/c6-1(2(7)8)5(13,3(9)10)4(11)12/h13H,(H,7,8)(H,9,10)(H,11,12) | 0 | 0 | 1 | 0 | 0 | 0 | 0 |
| 109 | InChI=1S/C5H4O8/c6-1(7)5(2(8)9,3(10)11)4(12)13/h(H,6,7)(H,8,9)(H,10,11)(H,12,13) | 1 | 0 | 1 | 0 | 1 | 1 | 0 |
| 110 | InChI=1S/C5H6O5/c6-3(5(9)10)1-2-4(7)8/h1-3,6H,(H,7,8)(H,9,10) | 0 | 0 | 1 | 0 | 1 | 1 | 0 |
| 111 | InChI=1S/C5H6O5/c6-2-1-3(4(7)8)5(9)10/h1,6H,2H2,(H,7,8)(H,9,10) | 0 | 0 | 1 | 0 | 0 | 0 | 0 |
| 112 | InChI=1S/C5H6O5/c6-2-3(5(9)10)1-4(7)8/h1,6H,2H2,(H,7,8)(H,9,10) | 0 | 0 | 1 | 0 | 1 | 0 | 0 |
| 113 | InChI=1S/C5H6O5/c1-2-5(10,3(6)7)4(8)9/h2,10H,1H2,(H,6,7)(H,8,9) | 0 | 0 | 1 | 0 | 1 | 1 | 0 |
| 114 | InChI=1S/C5H6O5/c1-2(4(7)8)3(6)5(9)10/h3,6H,1H2,(H,7,8)(H,9,10) | 0 | 1 | 1 | 0 | 1 | 0 | 0 |
| 115 | InChI=1S/C5H6O5/c6-1-3(8)5(10)4(9)2-7/h6-7H,1-2H2 | 0 | 0 | 1 | 0 | 1 | 0 | 0 |
| 116 | InChI=1S/C5H6O5/c6-2-1-3(7)4(8)5(9)10/h6H,1-2H2,(H,9,10) | 0 | 0 | 1 | 0 | 1 | 0 | 0 |
| 117 | InChI=1S/C5H6O5/c6-2-4(8)3(7)1-5(9)10/h6H,1-2H2,(H,9,10) | 0 | 0 | 1 | 0 | 0 | 0 | 0 |
| 118 | InChI=1S/C5H6O5/c6-2-3(7)1-4(8)5(9)10/h6H,1-2H2,(H,9,10) | 0 | 0 | 1 | 0 | 1 | 0 | 0 |
| 119 | InChI=1S/C5H6O5/c6-3(5(9)10)1-2-4(7)8/h1-2H2,(H,7,8)(H,9,10) | 1 | 1 | 1 | 1 | 1 | 1 | 1 |
| 120 | InChI=1S/C5H6O5/c6-3(1-4(7)8)2-5(9)10/h1-2H2,(H,7,8)(H,9,10) | 1 | 0 | 1 | 1 | 1 | 1 | 0 |
| 121 | InChI=1S/C5H6O5/c6-1-3(8)5(10)4(9)2-7/h1,4,7,9H,2H2 | 0 | 0 | 1 | 0 | 0 | 0 | 0 |
| 122 | InChI=1S/C5H6O5/c6-1-3(8)5(10)4(9)2-7/h1,3,7-8H,2H2 | 0 | 0 | 1 | 0 | 1 | 0 | 0 |
| 123 | InChI=1S/C5H6O5/c6-1-3(2-7)4(8)5(9)10/h1,3,7H,2H2,(H,9,10) | 0 | 0 | 1 | 0 | 0 | 0 | 0 |
| 124 | InChI=1S/C5H6O5/c6-2-1-3(7)4(8)5(9)10/h2-3,7H,1H2,(H,9,10) | 0 | 0 | 1 | 0 | 0 | 0 | 0 |
| 125 | InChI=1S/C5H6O5/c6-2-3(7)1-4(8)5(9)10/h2-3,7H,1H2,(H,9,10) | 0 | 0 | 1 | 0 | 1 | 0 | 0 |
| 126 | InChI=1S/C5H6O5/c6-1-3(8)5(10)4(9)2-7/h1,5,7,10H,2H2 | 0 | 0 | 1 | 0 | 0 | 0 | 0 |
| 127 | InChI=1S/C5H6O5/c6-1-3(5(9)10)4(8)2-7/h2-3,6H,1H2,(H,9,10) | 0 | 0 | 1 | 0 | 0 | 0 | 0 |
| 128 | InChI=1S/C5H6O5/c6-2-4(8)3(7)1-5(9)10/h2-3,7H,1H2,(H,9,10) | 0 | 0 | 1 | 0 | 0 | 0 | 0 |
| 129 | InChI=1S/C5H6O5/c6-2-3(7)1-4(8)5(9)10/h2,4,8H,1H2,(H,9,10) | 0 | 0 | 1 | 0 | 0 | 0 | 0 |
| 130 | InChI=1S/C5H6O5/c6-1-3(5(9)10)4(8)2-7/h1,3,7H,2H2,(H,9,10) | 0 | 0 | 1 | 0 | 0 | 0 | 0 |
| 131 | InChI=1S/C5H6O5/c6-2-1-3(7)4(8)5(9)10/h2,4,8H,1H2,(H,9,10) | 0 | 0 | 1 | 0 | 0 | 0 | 0 |
| 132 | InChI=1S/C5H6O5/c6-2-4(8)3(7)1-5(9)10/h2,4,8H,1H2,(H,9,10) | 0 | 0 | 1 | 0 | 0 | 0 | 0 |
| 133 | InChI=1S/C5H6O5/c6-2-1-3(4(7)8)5(9)10/h2-3H,1H2,(H,7,8)(H,9,10) | 0 | 0 | 1 | 0 | 1 | 0 | 0 |
| 134 | InChI=1S/C5H6O5/c6-2-3(5(9)10)1-4(7)8/h2-3H,1H2,(H,7,8)(H,9,10) | 1 | 0 | 1 | 0 | 1 | 1 | 0 |
| 135 | InChI=1S/C5H6O5/c1-2(6)3(7)4(8)5(9)10/h2,6H,1H3,(H,9,10) | 0 | 0 | 1 | 0 | 1 | 0 | 0 |
| 136 | InChI=1S/C5H6O5/c1-2(6)3(7)4(8)5(9)10/h4,8H,1H3,(H,9,10) | 0 | 0 | 1 | 0 | 0 | 0 | 0 |
| 137 | InChI=1S/C5H6O5/c1-2(6)3(7)4(8)5(9)10/h3,7H,1H3,(H,9,10) | 0 | 0 | 1 | 0 | 1 | 0 | 0 |
| 138 | InChI=1S/C5H6O5/c1-2(4(7)8)3(6)5(9)10/h2H,1H3,(H,7,8)(H,9,10) | 1 | 1 | 1 | 0 | 1 | 1 | 0 |
| 139 | InChI=1S/C5H6O5/c1-2(6)3(4(7)8)5(9)10/h3H,1H3,(H,7,8)(H,9,10) | 0 | 0 | 1 | 0 | 1 | 1 | 0 |
| 140 | InChI=1S/C5H6O5/c6-1-3(8)5(10)4(9)2-7/h1-3,5,8,10H | 0 | 0 | 1 | 0 | 0 | 0 | 0 |
| 141 | InChI=1S/C5H6O5/c6-1-3(8)5(10)4(9)2-7/h1-4,8-9H | 0 | 0 | 1 | 0 | 0 | 0 | 0 |
| 142 | InChI=1S/C5H6O5/c6-1-3(5(9)10)4(8)2-7/h1-4,8H,(H,9,10) | 0 | 0 | 1 | 0 | 0 | 0 | 0 |
| 143 | InChI=1S/C5H6O5/c6-1-3(2-7)4(8)5(9)10/h1-4,8H,(H,9,10) | 0 | 0 | 1 | 0 | 0 | 0 | 0 |
| 144 | InChI=1S/C5H6O5/c6-1-4(9)5(10,2-7)3-8/h1-2,8,10H,3H2 | 0 | 0 | 1 | 0 | 0 | 0 | 0 |
| 145 | InChI=1S/C5H6O5/c6-1-4(9)5(10,2-7)3-8/h2-3,6,10H,1H2 | 0 | 0 | 1 | 0 | 0 | 0 | 0 |
| 146 | InChI=1S/C5H6O5/c6-1-5(2-7,3-8)4(9)10/h1-2,8H,3H2,(H,9,10) | 0 | 0 | 1 | 0 | 0 | 0 | 0 |
| 147 | InChI=1S/C5H6O5/c6-2-1-5(10,3-7)4(8)9/h2-3,10H,1H2,(H,8,9) | 0 | 0 | 1 | 0 | 0 | 0 | 0 |
| 148 | InChI=1S/C5H6O5/c6-2-5(10,3-7)1-4(8)9/h2-3,10H,1H2,(H,8,9) | 0 | 0 | 1 | 0 | 0 | 0 | 0 |
| 149 | InChI=1S/C5H6O5/c6-1-4(9)5(10,2-7)3-8/h1-4,9-10H | 0 | 0 | 1 | 0 | 0 | 0 | 0 |
| 150 | InChI=1S/C5H6O5/c1-5(10,2-6)3(7)4(8)9/h2,10H,1H3,(H,8,9) | 0 | 0 | 1 | 0 | 0 | 0 | 0 |
| 151 | InChI=1S/C5H6O5/c1-5(10,4(8)9)3(7)2-6/h2,10H,1H3,(H,8,9) | 0 | 0 | 1 | 0 | 0 | 0 | 0 |
| 152 | InChI=1S/C5H6O5/c1-3(7)5(10,2-6)4(8)9/h2,10H,1H3,(H,8,9) | 0 | 0 | 1 | 0 | 0 | 0 | 0 |
| 153 | InChI=1S/C5H6O5/c1-5(2-6,3(7)8)4(9)10/h2H,1H3,(H,7,8)(H,9,10) | 0 | 0 | 1 | 0 | 1 | 0 | 0 |
| 154 | InChI=1S/C5H6O6/c6-1-3(8)5(11,2-7)4(9)10/h1-3,8,11H,(H,9,10) | 0 | 0 | 1 | 0 | 0 | 0 | 0 |
| 155 | InChI=1S/C5H6O6/c6-1-5(11,2-7)3(8)4(9)10/h1-3,8,11H,(H,9,10) | 0 | 0 | 1 | 0 | 0 | 0 | 0 |
| 156 | InChI=1S/C5H6O6/c6-1-5(11,2-7)3(8)4(9)10/h1,7,11H,2H2,(H,9,10) | 0 | 0 | 1 | 0 | 1 | 0 | 0 |
| 157 | InChI=1S/C5H6O6/c6-1-3(8)5(11,2-7)4(9)10/h1,7,11H,2H2,(H,9,10) | 0 | 0 | 1 | 0 | 0 | 0 | 0 |
| 158 | InChI=1S/C5H6O6/c6-1-3(8)5(11,2-7)4(9)10/h2,6,11H,1H2,(H,9,10) | 0 | 0 | 1 | 0 | 0 | 0 | 0 |
| 159 | InChI=1S/C5H6O6/c6-1-5(2-7,3(8)9)4(10)11/h1,7H,2H2,(H,8,9)(H,10,11) | 0 | 0 | 1 | 0 | 0 | 0 | 0 |
| 160 | InChI=1S/C5H6O6/c6-2-1-5(11,3(7)8)4(9)10/h2,11H,1H2,(H,7,8)(H,9,10) | 0 | 0 | 1 | 0 | 0 | 0 | 0 |
| 161 | InChI=1S/C5H6O6/c6-2-5(11,4(9)10)1-3(7)8/h2,11H,1H2,(H,7,8)(H,9,10) | 0 | 0 | 1 | 0 | 1 | 0 | 0 |
| 162 | InChI=1S/C5H6O6/c1-5(11,4(9)10)2(6)3(7)8/h11H,1H3,(H,7,8)(H,9,10) | 1 | 0 | 1 | 0 | 0 | 1 | 0 |
| 163 | InChI=1S/C5H6O6/c1-2(6)5(11,3(7)8)4(9)10/h11H,1H3,(H,7,8)(H,9,10) | 0 | 0 | 1 | 0 | 0 | 0 | 0 |
| 164 | InChI=1S/C5H6O6/c1-5(2(6)7,3(8)9)4(10)11/h1H3,(H,6,7)(H,8,9)(H,10,11) | 0 | 1 | 1 | 0 | 1 | 0 | 0 |
| 165 | InChI=1S/C5H6O6/c6-1-2(7)3(8)4(9)5(10)11/h1-3,7-8H,(H,10,11) | 0 | 0 | 1 | 0 | 0 | 0 | 0 |
| 166 | InChI=1S/C5H6O6/c6-1-2(7)3(8)4(9)5(10)11/h1,3-4,8-9H,(H,10,11) | 0 | 0 | 1 | 0 | 0 | 0 | 0 |
| 167 | InChI=1S/C5H6O6/c6-1-2(7)3(8)4(9)5(10)11/h1-2,4,7,9H,(H,10,11) | 0 | 0 | 1 | 0 | 0 | 0 | 0 |
| 168 | InChI=1S/C5H6O6/c6-1-2(7)3(4(8)9)5(10)11/h1-3,7H,(H,8,9)(H,10,11) | 0 | 0 | 1 | 0 | 0 | 0 | 0 |
| 169 | InChI=1S/C5H6O6/c6-1-2(4(8)9)3(7)5(10)11/h1-3,7H,(H,8,9)(H,10,11) | 0 | 0 | 1 | 0 | 1 | 0 | 0 |
| 170 | InChI=1S/C5H6O6/c6-1-2(7)3(8)4(9)5(10)11/h2,6-7H,1H2,(H,10,11) | 0 | 0 | 1 | 0 | 0 | 0 | 0 |
| 171 | InChI=1S/C5H6O6/c6-1-2(7)3(8)4(9)5(10)11/h4,6,9H,1H2,(H,10,11) | 0 | 0 | 1 | 0 | 0 | 0 | 0 |
| 172 | InChI=1S/C5H6O6/c6-1-2(7)3(8)4(9)5(10)11/h3,6,8H,1H2,(H,10,11) | 0 | 0 | 1 | 0 | 0 | 0 | 0 |
| 173 | InChI=1S/C5H6O6/c6-1-2(4(8)9)3(7)5(10)11/h2,6H,1H2,(H,8,9)(H,10,11) | 1 | 0 | 1 | 0 | 0 | 1 | 0 |
| 174 | InChI=1S/C5H6O6/c6-2(1-3(7)8)4(9)5(10)11/h2,6H,1H2,(H,7,8)(H,10,11) | 0 | 0 | 1 | 0 | 1 | 1 | 0 |
| 175 | InChI=1S/C5H6O6/c6-2(4(8)9)1-3(7)5(10)11/h2,6H,1H2,(H,8,9)(H,10,11) | 1 | 1 | 1 | 0 | 1 | 1 | 0 |
| 176 | InChI=1S/C5H6O6/c6-1-2(7)3(4(8)9)5(10)11/h3,6H,1H2,(H,8,9)(H,10,11) | 0 | 0 | 1 | 0 | 0 | 0 | 0 |
| 177 | InChI=1S/C5H6O6/c6-2(1-3(7)8)4(9)5(10)11/h4,9H,1H2,(H,7,8)(H,10,11) | 0 | 0 | 1 | 0 | 1 | 0 | 0 |
| 178 | InChI=1S/C5H6O6/c6-3(7)1-2(4(8)9)5(10)11/h2H,1H2,(H,6,7)(H,8,9)(H,10,11) | 1 | 1 | 1 | 0 | 1 | 1 | 0 |
| 179 | InChI=1S/C5H6O7/c6-1-2(7)5(12,3(8)9)4(10)11/h1-2,7,12H,(H,8,9)(H,10,11) | 0 | 0 | 1 | 0 | 0 | 0 | 0 |
| 180 | InChI=1S/C5H6O7/c6-1-5(12,4(10)11)2(7)3(8)9/h1-2,7,12H,(H,8,9)(H,10,11) | 0 | 0 | 1 | 0 | 1 | 0 | 0 |
| 181 | InChI=1S/C5H6O7/c6-1-5(12,4(10)11)2(7)3(8)9/h6,12H,1H2,(H,8,9)(H,10,11) | 0 | 0 | 1 | 0 | 0 | 0 | 0 |
| 182 | InChI=1S/C5H6O7/c6-1-2(7)5(12,3(8)9)4(10)11/h6,12H,1H2,(H,8,9)(H,10,11) | 0 | 0 | 1 | 0 | 0 | 0 | 0 |
| 183 | InChI=1S/C5H6O7/c6-1-5(2(7)8,3(9)10)4(11)12/h6H,1H2,(H,7,8)(H,9,10)(H,11,12) | 0 | 0 | 1 | 0 | 1 | 1 | 0 |
| 184 | InChI=1S/C5H6O7/c6-2(7)1-5(12,3(8)9)4(10)11/h12H,1H2,(H,6,7)(H,8,9)(H,10,11) | 1 | 1 | 1 | 0 | 1 | 1 | 0 |
| 185 | InChI=1S/C5H6O7/c6-1(2(7)4(9)10)3(8)5(11)12/h1-2,6-7H,(H,9,10)(H,11,12) | 1 | 0 | 1 | 0 | 1 | 1 | 0 |
| 186 | InChI=1S/C5H6O7/c6-1(2(7)4(9)10)3(8)5(11)12/h2-3,7-8H,(H,9,10)(H,11,12) | 0 | 0 | 1 | 0 | 1 | 0 | 0 |
| 187 | InChI=1S/C5H6O7/c6-2(5(11)12)1(3(7)8)4(9)10/h1-2,6H,(H,7,8)(H,9,10)(H,11,12) | 1 | 1 | 1 | 0 | 1 | 1 | 0 |
| 188 | InChI=1S/C5H6O8/c6-1(2(7)8)5(13,3(9)10)4(11)12/h1,6,13H,(H,7,8)(H,9,10)(H,11,12) | 1 | 0 | 1 | 0 | 1 | 1 | 0 |
| 189 | InChI=1S/C5H8O6/c6-1-3(8)5(11,2-7)4(9)10/h2-3,6,8,11H,1H2,(H,9,10) | 0 | 0 | 1 | 0 | 0 | 0 | 0 |
| 190 | InChI=1S/C5H8O6/c6-1-3(8)5(11,2-7)4(9)10/h1,3,7-8,11H,2H2,(H,9,10) | 0 | 0 | 1 | 0 | 0 | 0 | 0 |
| 191 | InChI=1S/C5H8O6/c6-1-5(11,2-7)3(8)4(9)10/h1,3,7-8,11H,2H2,(H,9,10) | 0 | 0 | 1 | 0 | 0 | 0 | 0 |
| 192 | InChI=1S/C5H8O6/c6-1-2(7)3(8)4(9)5(10)11/h1-4,7-9H,(H,10,11) | 1 | 0 | 1 | 0 | 1 | 1 | 0 |
| 193 | InChI=1S/C5H8O6/c6-1-2(7)3(8)4(9)5(10)11/h2-3,6-8H,1H2,(H,10,11) | 1 | 0 | 1 | 0 | 1 | 1 | 0 |
| 194 | InChI=1S/C5H8O6/c6-1-2(7)3(8)4(9)5(10)11/h2,4,6-7,9H,1H2,(H,10,11) | 0 | 0 | 1 | 0 | 0 | 0 | 0 |
| 195 | InChI=1S/C5H8O6/c6-1-2(7)3(8)4(9)5(10)11/h3-4,6,8-9H,1H2,(H,10,11) | 1 | 0 | 1 | 0 | 1 | 1 | 0 |
| 196 | InChI=1S/C5H8O6/c6-1-2(7)3(4(8)9)5(10)11/h2-3,6-7H,1H2,(H,8,9)(H,10,11) | 0 | 0 | 1 | 0 | 1 | 0 | 0 |
| 197 | InChI=1S/C5H8O6/c6-1-2(4(8)9)3(7)5(10)11/h2-3,6-7H,1H2,(H,8,9)(H,10,11) | 0 | 1 | 1 | 0 | 1 | 0 | 0 |
| 198 | InChI=1S/C5H8O6/c6-2(4(8)9)1-3(7)5(10)11/h2-3,6-7H,1H2,(H,8,9)(H,10,11) | 1 | 1 | 1 | 0 | 1 | 1 | 0 |
| 199 | InChI=1S/C5H8O6/c6-2(1-3(7)8)4(9)5(10)11/h2,4,6,9H,1H2,(H,7,8)(H,10,11) | 1 | 0 | 1 | 0 | 1 | 1 | 0 |
| 200 | InChI=1S/C5H8O6/c1-2(6)5(11,3(7)8)4(9)10/h2,6,11H,1H3,(H,7,8)(H,9,10) | 0 | 0 | 1 | 0 | 0 | 0 | 0 |
| 201 | InChI=1S/C5H8O6/c1-5(11,4(9)10)2(6)3(7)8/h2,6,11H,1H3,(H,7,8)(H,9,10) | 1 | 1 | 1 | 0 | 1 | 1 | 0 |
| 202 | InChI=1S/C5H8O6/c6-1-5(11,2-7)3(8)4(9)10/h6-7,11H,1-2H2,(H,9,10) | 0 | 0 | 1 | 0 | 0 | 0 | 0 |
| 203 | InChI=1S/C5H8O6/c6-1-3(8)5(11,2-7)4(9)10/h6-7,11H,1-2H2,(H,9,10) | 0 | 0 | 1 | 0 | 1 | 1 | 0 |
| 204 | InChI=1S/C5H8O6/c6-1-5(2-7,3(8)9)4(10)11/h6-7H,1-2H2,(H,8,9)(H,10,11) | 1 | 0 | 1 | 1 | 1 | 1 | 0 |
| 205 | InChI=1S/C5H8O6/c6-2-1-5(11,3(7)8)4(9)10/h6,11H,1-2H2,(H,7,8)(H,9,10) | 0 | 0 | 1 | 0 | 1 | 0 | 0 |
| 206 | InChI=1S/C5H8O6/c6-2-5(11,4(9)10)1-3(7)8/h6,11H,1-2H2,(H,7,8)(H,9,10) | 1 | 0 | 1 | 0 | 1 | 1 | 0 |
| 207 | InChI=1S/C5H8O7/c6-1-2(7)5(12,3(8)9)4(10)11/h2,6-7,12H,1H2,(H,8,9)(H,10,11) | 0 | 0 | 1 | 0 | 1 | 0 | 0 |
| 208 | InChI=1S/C5H8O7/c6-1-5(12,4(10)11)2(7)3(8)9/h2,6-7,12H,1H2,(H,8,9)(H,10,11) | 1 | 0 | 1 | 0 | 1 | 1 | 0 |
| 209 | InChI=1S/C5H8O7/c6-1(2(7)4(9)10)3(8)5(11)12/h1-3,6-8H,(H,9,10)(H,11,12) | 1 | 0 | 1 | 1 | 1 | 1 | 0 |
| 210 | InChI=1S/C6H2O6/c7-1-3(9)5(11)6(12)4(10)2-8/h1-2H | 0 | 0 | 1 | 0 | 0 | 0 | 0 |
| 211 | InChI=1S/C6H2O7/c7-1-2(8)3(9)4(10)5(11)6(12)13/h1H,(H,12,13) | 0 | 0 | 1 | 0 | 0 | 0 | 0 |
| 212 | InChI=1S/C6H2O8/c7-1(3(9)5(11)12)2(8)4(10)6(13)14/h(H,11,12)(H,13,14) | 0 | 0 | 1 | 0 | 0 | 0 | 0 |
| 213 | InChI=1S/C6H4O6/c1-2(3(7)5(9)10)4(8)6(11)12/h1H2,(H,9,10)(H,11,12) | 0 | 0 | 1 | 0 | 0 | 0 | 0 |
| 214 | InChI=1S/C6H4O6/c1-2(5(9)10)3(7)4(8)6(11)12/h1H2,(H,9,10)(H,11,12) | 0 | 0 | 1 | 0 | 0 | 0 | 0 |
| 215 | InChI=1S/C6H4O6/c7-1-3(2-8)4(5(9)10)6(11)12/h1-2H,(H,9,10)(H,11,12) | 0 | 0 | 1 | 0 | 0 | 0 | 0 |
| 216 | InChI=1S/C6H4O6/c7-1-3(5(9)10)4(2-8)6(11)12/h1-2H,(H,9,10)(H,11,12) | 0 | 0 | 1 | 0 | 0 | 0 | 0 |
| 217 | InChI=1S/C6H4O6/c7-2-1-3(5(9)10)4(8)6(11)12/h1-2H,(H,9,10)(H,11,12) | 0 | 0 | 1 | 0 | 0 | 0 | 0 |
| 218 | InChI=1S/C6H4O6/c7-2-4(8)3(6(11)12)1-5(9)10/h1-2H,(H,9,10)(H,11,12) | 0 | 0 | 1 | 0 | 0 | 0 | 0 |
| 219 | InChI=1S/C6H4O6/c7-2-3(8)1-4(5(9)10)6(11)12/h1-2H,(H,9,10)(H,11,12) | 0 | 0 | 1 | 0 | 0 | 0 | 0 |
| 220 | InChI=1S/C6H4O6/c7-2-3(1-4(8)9)5(10)6(11)12/h1-2H,(H,8,9)(H,11,12) | 0 | 0 | 1 | 0 | 0 | 0 | 0 |
| 221 | InChI=1S/C6H4O6/c7-3(1-2-4(8)9)5(10)6(11)12/h1-2H,(H,8,9)(H,11,12) | 1 | 0 | 1 | 0 | 1 | 1 | 0 |
| 222 | InChI=1S/C6H4O6/c7-2-3(5(9)10)1-4(8)6(11)12/h1-2H,(H,9,10)(H,11,12) | 0 | 0 | 1 | 0 | 0 | 0 | 0 |
| 223 | InChI=1S/C6H4O6/c7-3(5(9)10)1-2-4(8)6(11)12/h1-2H,(H,9,10)(H,11,12) | 0 | 0 | 1 | 0 | 0 | 0 | 0 |
| 224 | InChI=1S/C6H4O6/c1-2(7)3(8)4(9)5(10)6(11)12/h1H3,(H,11,12) | 0 | 0 | 1 | 0 | 0 | 0 | 0 |
| 225 | InChI=1S/C6H4O6/c7-1-3(9)5(11)6(12)4(10)2-8/h1-3,9H | 0 | 0 | 1 | 0 | 0 | 0 | 0 |
| 226 | InChI=1S/C6H4O6/c7-1-3(2-8)4(9)5(10)6(11)12/h1-3H,(H,11,12) | 0 | 0 | 1 | 0 | 0 | 0 | 0 |
| 227 | InChI=1S/C6H4O6/c7-1-3(9)5(11)6(12)4(10)2-8/h1-2,5,11H | 0 | 0 | 1 | 0 | 0 | 0 | 0 |
| 228 | InChI=1S/C6H4O6/c7-1-3(6(11)12)5(10)4(9)2-8/h1-3H,(H,11,12) | 0 | 0 | 1 | 0 | 0 | 0 | 0 |
| 229 | InChI=1S/C6H4O6/c7-1-3(4(9)2-8)5(10)6(11)12/h1-3H,(H,11,12) | 0 | 0 | 1 | 0 | 0 | 0 | 0 |
| 230 | InChI=1S/C6H4O6/c7-1-3(9)5(6(11)12)4(10)2-8/h1-2,5H,(H,11,12) | 0 | 0 | 1 | 0 | 0 | 0 | 0 |
| 231 | InChI=1S/C6H4O6/c7-1-4(10)5(11)6(12,2-8)3-9/h1-3,12H | 0 | 0 | 1 | 0 | 0 | 0 | 0 |
| 232 | InChI=1S/C6H4O6/c7-1-6(2-8,3-9)4(10)5(11)12/h1-3H,(H,11,12) | 0 | 0 | 1 | 0 | 0 | 0 | 0 |
| 233 | InChI=1S/C6H4O6/c7-1-4(10)6(12,3-9)5(11)2-8/h1-3,12H | 0 | 0 | 1 | 0 | 0 | 0 | 0 |
| 234 | InChI=1S/C6H4O6/c7-1-4(10)6(2-8,3-9)5(11)12/h1-3H,(H,11,12) | 0 | 0 | 1 | 0 | 0 | 0 | 0 |
| 235 | InChI=1S/C6H4O6/c7-1-3(9)5(11)6(12)4(10)2-8/h1,8H,2H2 | 0 | 0 | 1 | 0 | 0 | 0 | 0 |
| 236 | InChI=1S/C6H4O6/c7-2-1-3(8)4(9)5(10)6(11)12/h2H,1H2,(H,11,12) | 0 | 0 | 1 | 0 | 0 | 0 | 0 |
| 237 | InChI=1S/C6H4O6/c7-2-4(9)6(12)3(8)1-5(10)11/h2H,1H2,(H,10,11) | 0 | 0 | 1 | 0 | 0 | 0 | 0 |
| 238 | InChI=1S/C6H4O6/c7-2-3(8)1-4(9)5(10)6(11)12/h2H,1H2,(H,11,12) | 0 | 0 | 1 | 0 | 0 | 0 | 0 |
| 239 | InChI=1S/C6H4O6/c7-2-5(10)3(8)1-4(9)6(11)12/h2H,1H2,(H,11,12) | 0 | 0 | 1 | 0 | 0 | 0 | 0 |
| 240 | InChI=1S/C6H4O7/c7-1-2(4(8)9)3(5(10)11)6(12)13/h1H,(H,8,9)(H,10,11)(H,12,13) | 0 | 0 | 1 | 0 | 0 | 0 | 0 |
| 241 | InChI=1S/C6H4O7/c7-3(8)1-2(5(10)11)4(9)6(12)13/h1H,(H,7,8)(H,10,11)(H,12,13) | 0 | 0 | 1 | 0 | 0 | 0 | 0 |
| 242 | InChI=1S/C6H4O7/c7-3(6(12)13)1-2(4(8)9)5(10)11/h1H,(H,8,9)(H,10,11)(H,12,13) | 0 | 0 | 1 | 0 | 0 | 0 | 0 |
| 243 | InChI=1S/C6H4O7/c7-1-2(8)3(9)4(10)5(11)6(12)13/h7H,1H2,(H,12,13) | 0 | 0 | 1 | 0 | 0 | 0 | 0 |
| 244 | InChI=1S/C6H4O7/c7-2(1-3(8)9)4(10)5(11)6(12)13/h1H2,(H,8,9)(H,12,13) | 0 | 0 | 1 | 0 | 0 | 0 | 0 |
| 245 | InChI=1S/C6H4O7/c7-2(4(9)6(12)13)1-3(8)5(10)11/h1H2,(H,10,11)(H,12,13) | 0 | 0 | 1 | 0 | 0 | 0 | 0 |
| 246 | InChI=1S/C6H4O7/c7-1-2(8)3(9)4(10)5(11)6(12)13/h1-2,8H,(H,12,13) | 0 | 0 | 1 | 0 | 0 | 0 | 0 |
| 247 | InChI=1S/C6H4O7/c7-1-2(8)3(9)4(10)5(11)6(12)13/h1,5,11H,(H,12,13) | 0 | 0 | 1 | 0 | 0 | 0 | 0 |
| 248 | InChI=1S/C6H4O7/c7-1-2(8)3(9)4(10)5(11)6(12)13/h1,3,9H,(H,12,13) | 0 | 0 | 1 | 0 | 0 | 0 | 0 |
| 249 | InChI=1S/C6H4O7/c7-1-2(5(10)11)3(8)4(9)6(12)13/h1-2H,(H,10,11)(H,12,13) | 0 | 0 | 1 | 0 | 0 | 0 | 0 |
| 250 | InChI=1S/C6H4O7/c7-1-2(8)3(9)4(10)5(11)6(12)13/h1,4,10H,(H,12,13) | 0 | 0 | 1 | 0 | 0 | 0 | 0 |
| 251 | InChI=1S/C6H4O7/c7-1-2(8)4(9)3(5(10)11)6(12)13/h1,3H,(H,10,11)(H,12,13) | 0 | 0 | 1 | 0 | 0 | 0 | 0 |
| 252 | InChI=1S/C6H4O7/c7-1-2(3(8)5(10)11)4(9)6(12)13/h1-2H,(H,10,11)(H,12,13) | 0 | 0 | 1 | 0 | 0 | 0 | 0 |
| 253 | InChI=1S/C6H4O7/c7-1-2(8)3(5(10)11)4(9)6(12)13/h1,3H,(H,10,11)(H,12,13) | 0 | 0 | 1 | 0 | 0 | 0 | 0 |
| 254 | InChI=1S/C6H4O7/c7-1-6(13,2-8)4(10)3(9)5(11)12/h1-2,13H,(H,11,12) | 0 | 0 | 1 | 0 | 0 | 0 | 0 |
| 255 | InChI=1S/C6H4O7/c7-1-3(9)4(10)6(13,2-8)5(11)12/h1-2,13H,(H,11,12) | 0 | 0 | 1 | 0 | 0 | 0 | 0 |
| 256 | InChI=1S/C6H4O7/c7-1-3(9)6(13,2-8)4(10)5(11)12/h1-2,13H,(H,11,12) | 0 | 0 | 1 | 0 | 0 | 0 | 0 |
| 257 | InChI=1S/C6H4O7/c7-1-6(2-8,5(12)13)3(9)4(10)11/h1-2H,(H,10,11)(H,12,13) | 0 | 0 | 1 | 0 | 0 | 0 | 0 |
| 258 | InChI=1S/C6H4O7/c7-1-3(9)6(13,5(11)12)4(10)2-8/h1-2,13H,(H,11,12) | 0 | 0 | 1 | 0 | 0 | 0 | 0 |
| 259 | InChI=1S/C6H4O7/c7-1-3(9)6(2-8,4(10)11)5(12)13/h1-2H,(H,10,11)(H,12,13) | 0 | 0 | 1 | 0 | 0 | 0 | 0 |
| 260 | InChI=1S/C6H4O8/c7-3(8)1(4(9)10)2(5(11)12)6(13)14/h(H,7,8)(H,9,10)(H,11,12)(H,13,14) | 1 | 0 | 1 | 0 | 1 | 1 | 0 |
| 261 | InChI=1S/C6H4O8/c7-1-6(14,5(12)13)3(9)2(8)4(10)11/h1,14H,(H,10,11)(H,12,13) | 0 | 0 | 1 | 0 | 0 | 0 | 0 |
| 262 | InChI=1S/C6H4O8/c7-1-2(8)3(9)6(14,4(10)11)5(12)13/h1,14H,(H,10,11)(H,12,13) | 0 | 0 | 1 | 0 | 0 | 0 | 0 |
| 263 | InChI=1S/C6H4O8/c7-1-6(14,2(8)4(10)11)3(9)5(12)13/h1,14H,(H,10,11)(H,12,13) | 0 | 0 | 1 | 0 | 0 | 0 | 0 |
| 264 | InChI=1S/C6H4O8/c7-1-2(8)6(14,5(12)13)3(9)4(10)11/h1,14H,(H,10,11)(H,12,13) | 0 | 0 | 1 | 0 | 0 | 0 | 0 |
| 265 | InChI=1S/C6H4O8/c7-1-6(4(11)12,5(13)14)2(8)3(9)10/h1H,(H,9,10)(H,11,12)(H,13,14) | 0 | 0 | 1 | 0 | 0 | 0 | 0 |
| 266 | InChI=1S/C6H4O8/c7-1-2(8)6(3(9)10,4(11)12)5(13)14/h1H,(H,9,10)(H,11,12)(H,13,14) | 0 | 0 | 1 | 0 | 0 | 0 | 0 |
| 267 | InChI=1S/C6H4O8/c7-1(3(9)5(11)12)2(8)4(10)6(13)14/h3,9H,(H,11,12)(H,13,14) | 0 | 0 | 1 | 0 | 0 | 0 | 0 |
| 268 | InChI=1S/C6H4O8/c7-1(3(9)5(11)12)2(8)4(10)6(13)14/h1,7H,(H,11,12)(H,13,14) | 0 | 0 | 1 | 0 | 0 | 0 | 0 |
| 269 | InChI=1S/C6H4O8/c7-2(3(8)6(13)14)1(4(9)10)5(11)12/h1H,(H,9,10)(H,11,12)(H,13,14) | 0 | 0 | 1 | 0 | 0 | 0 | 0 |
| 270 | InChI=1S/C6H4O8/c7-2(5(11)12)1(4(9)10)3(8)6(13)14/h1H,(H,9,10)(H,11,12)(H,13,14) | 0 | 0 | 1 | 0 | 0 | 0 | 0 |
| 271 | InChI=1S/C6H4O9/c7-1(3(9)10)2(8)6(15,4(11)12)5(13)14/h15H,(H,9,10)(H,11,12)(H,13,14) | 0 | 0 | 1 | 0 | 0 | 0 | 0 |
| 272 | InChI=1S/C6H4O9/c7-1(3(9)10)6(15,5(13)14)2(8)4(11)12/h15H,(H,9,10)(H,11,12)(H,13,14) | 0 | 0 | 1 | 0 | 0 | 0 | 0 |
| 273 | InChI=1S/C6H4O9/c7-1(2(8)9)6(3(10)11,4(12)13)5(14)15/h(H,8,9)(H,10,11)(H,12,13)(H,14,15) | 0 | 0 | 1 | 0 | 0 | 0 | 0 |
| 274 | InChI=1S/C6H6O6/c1-2(4(7)8)3(5(9)10)6(11)12/h1H3,(H,7,8)(H,9,10)(H,11,12) | 1 | 0 | 1 | 0 | 1 | 1 | 0 |
| 275 | InChI=1S/C6H6O6/c7-2-3(8)1-4(5(9)10)6(11)12/h1-3,8H,(H,9,10)(H,11,12) | 0 | 0 | 1 | 0 | 0 | 0 | 0 |
| 276 | InChI=1S/C6H6O6/c7-2-3(5(9)10)1-4(8)6(11)12/h1-2,4,8H,(H,9,10)(H,11,12) | 0 | 0 | 1 | 0 | 0 | 0 | 0 |
| 277 | InChI=1S/C6H6O6/c7-2-4(8)3(6(11)12)1-5(9)10/h1-2,4,8H,(H,9,10)(H,11,12) | 0 | 0 | 1 | 0 | 0 | 0 | 0 |
| 278 | InChI=1S/C6H6O6/c7-2-1-3(5(9)10)4(8)6(11)12/h1-2,4,8H,(H,9,10)(H,11,12) | 0 | 0 | 1 | 0 | 0 | 0 | 0 |
| 279 | InChI=1S/C6H6O6/c7-3(5(9)10)1-2-4(8)6(11)12/h1-3,7H,(H,9,10)(H,11,12) | 0 | 0 | 1 | 0 | 0 | 0 | 0 |
| 280 | InChI=1S/C6H6O6/c7-3(1-2-4(8)9)5(10)6(11)12/h1-3,7H,(H,8,9)(H,11,12) | 0 | 0 | 1 | 0 | 0 | 0 | 0 |
| 281 | InChI=1S/C6H6O6/c7-2-3(1-4(8)9)5(10)6(11)12/h1-2,5,10H,(H,8,9)(H,11,12) | 0 | 0 | 1 | 0 | 0 | 0 | 0 |
| 282 | InChI=1S/C6H6O6/c7-3(1-2-4(8)9)5(10)6(11)12/h1-2,5,10H,(H,8,9)(H,11,12) | 0 | 0 | 1 | 0 | 1 | 0 | 0 |
| 283 | InChI=1S/C6H6O6/c7-4(8)2-1-3(5(9)10)6(11)12/h1-3H,(H,7,8)(H,9,10)(H,11,12) | 0 | 0 | 1 | 0 | 1 | 0 | 0 |
| 284 | InChI=1S/C6H6O6/c7-3-1-2-6(12,4(8)9)5(10)11/h1-3,12H,(H,8,9)(H,10,11) | 0 | 0 | 1 | 0 | 0 | 0 | 0 |
| 285 | InChI=1S/C6H6O6/c7-3-6(12,5(10)11)2-1-4(8)9/h1-3,12H,(H,8,9)(H,10,11) | 0 | 0 | 1 | 0 | 0 | 0 | 0 |
| 286 | InChI=1S/C6H6O6/c7-1-3(2-8)4(5(9)10)6(11)12/h1,8H,2H2,(H,9,10)(H,11,12) | 0 | 0 | 1 | 0 | 0 | 0 | 0 |
| 287 | InChI=1S/C6H6O6/c7-1-3(5(9)10)4(2-8)6(11)12/h1,8H,2H2,(H,9,10)(H,11,12) | 0 | 0 | 1 | 0 | 0 | 0 | 0 |
| 288 | InChI=1S/C6H6O6/c7-2-1-3(5(9)10)4(8)6(11)12/h1,7H,2H2,(H,9,10)(H,11,12) | 0 | 0 | 1 | 0 | 0 | 0 | 0 |
| 289 | InChI=1S/C6H6O6/c7-2-4(8)3(6(11)12)1-5(9)10/h1,7H,2H2,(H,9,10)(H,11,12) | 0 | 0 | 1 | 0 | 0 | 0 | 0 |
| 290 | InChI=1S/C6H6O6/c7-2-3(8)1-4(5(9)10)6(11)12/h1,7H,2H2,(H,9,10)(H,11,12) | 0 | 0 | 1 | 0 | 0 | 0 | 0 |
| 291 | InChI=1S/C6H6O6/c7-4(8)2-1-3(5(9)10)6(11)12/h1H,2H2,(H,7,8)(H,9,10)(H,11,12) | 0 | 0 | 1 | 0 | 1 | 0 | 0 |
| 292 | InChI=1S/C6H6O6/c7-2-3(1-4(8)9)5(10)6(11)12/h1,7H,2H2,(H,8,9)(H,11,12) | 0 | 0 | 1 | 0 | 0 | 0 | 0 |
| 293 | InChI=1S/C6H6O6/c7-2-3(5(9)10)1-4(8)6(11)12/h1,7H,2H2,(H,9,10)(H,11,12) | 0 | 0 | 1 | 0 | 0 | 0 | 0 |
| 294 | InChI=1S/C6H6O6/c7-4(8)1-3(6(11)12)2-5(9)10/h1H,2H2,(H,7,8)(H,9,10)(H,11,12) | 1 | 1 | 1 | 1 | 1 | 1 | 1 |
| 295 | InChI=1S/C6H6O6/c1-2(5(9)10)3(7)4(8)6(11)12/h4,8H,1H2,(H,9,10)(H,11,12) | 0 | 0 | 1 | 0 | 0 | 0 | 0 |
| 296 | InChI=1S/C6H6O6/c1-2(3(7)5(9)10)4(8)6(11)12/h3,7H,1H2,(H,9,10)(H,11,12) | 0 | 0 | 1 | 0 | 0 | 0 | 0 |
| 297 | InChI=1S/C6H6O6/c1-2(5(9)10)3(7)4(8)6(11)12/h3,7H,1H2,(H,9,10)(H,11,12) | 0 | 0 | 1 | 0 | 0 | 0 | 0 |
| 298 | InChI=1S/C6H6O6/c1-2(4(7)8)3(5(9)10)6(11)12/h3H,1H2,(H,7,8)(H,9,10)(H,11,12) | 1 | 0 | 1 | 0 | 1 | 1 | 0 |
| 299 | InChI=1S/C6H6O6/c1-3(4(8)9)6(12,2-7)5(10)11/h2,12H,1H2,(H,8,9)(H,10,11) | 0 | 0 | 1 | 0 | 0 | 0 | 0 |
| 300 | InChI=1S/C6H6O6/c1-2-6(12,5(10)11)3(7)4(8)9/h2,12H,1H2,(H,8,9)(H,10,11) | 0 | 0 | 1 | 0 | 0 | 0 | 0 |
| 301 | InChI=1S/C6H6O6/c1-3(2-7)6(12,4(8)9)5(10)11/h2,12H,1H2,(H,8,9)(H,10,11) | 0 | 0 | 1 | 0 | 0 | 0 | 0 |
| 302 | InChI=1S/C6H6O6/c1-2-3(7)6(12,4(8)9)5(10)11/h2,12H,1H2,(H,8,9)(H,10,11) | 0 | 0 | 1 | 0 | 0 | 0 | 0 |
| 303 | InChI=1S/C6H6O6/c1-2-6(3(7)8,4(9)10)5(11)12/h2H,1H2,(H,7,8)(H,9,10)(H,11,12) | 0 | 0 | 1 | 0 | 1 | 0 | 0 |
| 304 | InChI=1S/C6H6O6/c7-1-3(9)5(11)6(12)4(10)2-8/h7-8H,1-2H2 | 0 | 0 | 1 | 0 | 0 | 0 | 0 |
| 305 | InChI=1S/C6H6O6/c7-2-1-3(8)4(9)5(10)6(11)12/h7H,1-2H2,(H,11,12) | 0 | 0 | 1 | 0 | 0 | 0 | 0 |
| 306 | InChI=1S/C6H6O6/c7-2-4(9)6(12)3(8)1-5(10)11/h7H,1-2H2,(H,10,11) | 0 | 0 | 1 | 0 | 0 | 0 | 0 |
| 307 | InChI=1S/C6H6O6/c7-2-3(8)1-4(9)5(10)6(11)12/h7H,1-2H2,(H,11,12) | 0 | 0 | 1 | 0 | 0 | 0 | 0 |
| 308 | InChI=1S/C6H6O6/c7-3(1-2-4(8)9)5(10)6(11)12/h1-2H2,(H,8,9)(H,11,12) | 0 | 1 | 1 | 0 | 1 | 1 | 0 |
| 309 | InChI=1S/C6H6O6/c7-2-5(10)3(8)1-4(9)6(11)12/h7H,1-2H2,(H,11,12) | 0 | 0 | 1 | 0 | 0 | 0 | 0 |
| 310 | InChI=1S/C6H6O6/c7-3(1-5(9)10)4(8)2-6(11)12/h1-2H2,(H,9,10)(H,11,12) | 1 | 0 | 1 | 0 | 1 | 1 | 0 |
| 311 | InChI=1S/C6H6O6/c7-3(5(9)10)1-2-4(8)6(11)12/h1-2H2,(H,9,10)(H,11,12) | 1 | 0 | 1 | 0 | 1 | 1 | 0 |
| 312 | InChI=1S/C6H6O6/c7-3(2-5(9)10)1-4(8)6(11)12/h1-2H2,(H,9,10)(H,11,12) | 0 | 0 | 1 | 0 | 1 | 0 | 0 |
| 313 | InChI=1S/C6H6O6/c7-1-3(9)5(11)6(12)4(10)2-8/h1,4,8,10H,2H2 | 0 | 0 | 1 | 0 | 0 | 0 | 0 |
| 314 | InChI=1S/C6H6O6/c7-1-3(9)5(11)6(12)4(10)2-8/h1,3,8-9H,2H2 | 0 | 0 | 1 | 0 | 1 | 0 | 0 |
| 315 | InChI=1S/C6H6O6/c7-1-3(2-8)4(9)5(10)6(11)12/h1,3,8H,2H2,(H,11,12) | 0 | 0 | 1 | 0 | 0 | 0 | 0 |
| 316 | InChI=1S/C6H6O6/c7-2-1-3(8)4(9)5(10)6(11)12/h2-3,8H,1H2,(H,11,12) | 0 | 0 | 1 | 0 | 0 | 0 | 0 |
| 317 | InChI=1S/C6H6O6/c7-2-3(8)1-4(9)5(10)6(11)12/h2-3,8H,1H2,(H,11,12) | 0 | 0 | 1 | 0 | 0 | 0 | 0 |
| 318 | InChI=1S/C6H6O6/c7-1-3(9)5(11)6(12)4(10)2-8/h1,6,8,12H,2H2 | 0 | 0 | 1 | 0 | 0 | 0 | 0 |
| 319 | InChI=1S/C6H6O6/c7-1-3(6(11)12)5(10)4(9)2-8/h2-3,7H,1H2,(H,11,12) | 0 | 0 | 1 | 0 | 0 | 0 | 0 |
| 320 | InChI=1S/C6H6O6/c7-2-4(9)6(12)3(8)1-5(10)11/h2-3,8H,1H2,(H,10,11) | 0 | 0 | 1 | 0 | 0 | 0 | 0 |
| 321 | InChI=1S/C6H6O6/c7-2-5(10)3(8)1-4(9)6(11)12/h2,4,9H,1H2,(H,11,12) | 0 | 0 | 1 | 0 | 0 | 0 | 0 |
| 322 | InChI=1S/C6H6O6/c7-1-3(9)5(11)6(12)4(10)2-8/h1,5,8,11H,2H2 | 0 | 0 | 1 | 0 | 0 | 0 | 0 |
| 323 | InChI=1S/C6H6O6/c7-1-3(6(11)12)5(10)4(9)2-8/h1,3,8H,2H2,(H,11,12) | 0 | 0 | 1 | 0 | 0 | 0 | 0 |
| 324 | InChI=1S/C6H6O6/c7-2-1-3(8)4(9)5(10)6(11)12/h2,5,10H,1H2,(H,11,12) | 0 | 0 | 1 | 0 | 0 | 0 | 0 |
| 325 | InChI=1S/C6H6O6/c7-2-4(9)6(12)3(8)1-5(10)11/h2,4,9H,1H2,(H,10,11) | 0 | 0 | 1 | 0 | 0 | 0 | 0 |
| 326 | InChI=1S/C6H6O6/c7-1-3(4(9)2-8)5(10)6(11)12/h2-3,7H,1H2,(H,11,12) | 0 | 0 | 1 | 0 | 0 | 0 | 0 |
| 327 | InChI=1S/C6H6O6/c7-2-3(8)1-4(9)5(10)6(11)12/h2,4,9H,1H2,(H,11,12) | 0 | 0 | 1 | 0 | 0 | 0 | 0 |
| 328 | InChI=1S/C6H6O6/c7-1-3(4(9)2-8)5(10)6(11)12/h1,3,8H,2H2,(H,11,12) | 0 | 0 | 1 | 0 | 0 | 0 | 0 |
| 329 | InChI=1S/C6H6O6/c7-2-1-3(8)4(9)5(10)6(11)12/h2,4,9H,1H2,(H,11,12) | 0 | 0 | 1 | 0 | 0 | 0 | 0 |
| 330 | InChI=1S/C6H6O6/c7-2-1-3(5(9)10)4(8)6(11)12/h2-3H,1H2,(H,9,10)(H,11,12) | 0 | 0 | 1 | 0 | 0 | 0 | 0 |
| 331 | InChI=1S/C6H6O6/c7-2-3(1-4(8)9)5(10)6(11)12/h2-3H,1H2,(H,8,9)(H,11,12) | 0 | 0 | 1 | 0 | 0 | 0 | 0 |
| 332 | InChI=1S/C6H6O6/c7-2-5(10)3(8)1-4(9)6(11)12/h2-3,8H,1H2,(H,11,12) | 0 | 0 | 1 | 0 | 0 | 0 | 0 |
| 333 | InChI=1S/C6H6O6/c7-2-5(10)3(8)1-4(9)6(11)12/h2,5,10H,1H2,(H,11,12) | 0 | 0 | 1 | 0 | 0 | 0 | 0 |
| 334 | InChI=1S/C6H6O6/c7-2-3(5(9)10)1-4(8)6(11)12/h2-3H,1H2,(H,9,10)(H,11,12) | 0 | 0 | 1 | 0 | 0 | 0 | 0 |
| 335 | InChI=1S/C6H6O6/c7-1-3(9)5(6(11)12)4(10)2-8/h1,5,8H,2H2,(H,11,12) | 0 | 0 | 1 | 0 | 0 | 0 | 0 |
| 336 | InChI=1S/C6H6O6/c7-2-4(9)6(12)3(8)1-5(10)11/h2,6,12H,1H2,(H,10,11) | 0 | 0 | 1 | 0 | 0 | 0 | 0 |
| 337 | InChI=1S/C6H6O6/c7-2-4(8)3(6(11)12)1-5(9)10/h2-3H,1H2,(H,9,10)(H,11,12) | 0 | 0 | 1 | 0 | 0 | 1 | 0 |
| 338 | InChI=1S/C6H6O6/c7-2-3(8)1-4(9)5(10)6(11)12/h2,5,10H,1H2,(H,11,12) | 0 | 0 | 1 | 0 | 0 | 0 | 0 |
| 339 | InChI=1S/C6H6O6/c7-2-3(8)1-4(5(9)10)6(11)12/h2,4H,1H2,(H,9,10)(H,11,12) | 0 | 0 | 1 | 0 | 0 | 0 | 0 |
| 340 | InChI=1S/C6H6O6/c7-2-1-3(8)4(5(9)10)6(11)12/h2,4H,1H2,(H,9,10)(H,11,12) | 0 | 0 | 1 | 0 | 0 | 0 | 0 |
| 341 | InChI=1S/C6H6O6/c7-2-3(6(11)12)4(8)1-5(9)10/h2-3H,1H2,(H,9,10)(H,11,12) | 0 | 0 | 1 | 0 | 0 | 0 | 0 |
| 342 | InChI=1S/C6H6O6/c7-1-3(9)5(11)6(12)4(10)2-8/h1-3,5,9,11H | 0 | 0 | 1 | 0 | 0 | 0 | 0 |
| 343 | InChI=1S/C6H6O6/c7-1-3(9)5(11)6(12)4(10)2-8/h1-4,9-10H | 0 | 0 | 1 | 0 | 0 | 0 | 0 |
| 344 | InChI=1S/C6H6O6/c7-1-3(4(9)2-8)5(10)6(11)12/h1-4,9H,(H,11,12) | 0 | 0 | 1 | 0 | 0 | 0 | 0 |
| 345 | InChI=1S/C6H6O6/c7-1-3(2-8)4(9)5(10)6(11)12/h1-4,9H,(H,11,12) | 0 | 0 | 1 | 0 | 0 | 0 | 0 |
| 346 | InChI=1S/C6H6O6/c7-1-3(9)5(11)6(12)4(10)2-8/h1-2,5-6,11-12H | 0 | 0 | 1 | 0 | 0 | 0 | 0 |
| 347 | InChI=1S/C6H6O6/c7-1-3(9)5(11)6(12)4(10)2-8/h1-3,6,9,12H | 0 | 0 | 1 | 0 | 0 | 0 | 0 |
| 348 | InChI=1S/C6H6O6/c7-1-3(9)5(6(11)12)4(10)2-8/h1-3,5,9H,(H,11,12) | 0 | 0 | 1 | 0 | 0 | 0 | 0 |
| 349 | InChI=1S/C6H6O6/c7-1-3(4(9)2-8)5(10)6(11)12/h1-3,5,10H,(H,11,12) | 0 | 0 | 1 | 0 | 0 | 0 | 0 |
| 350 | InChI=1S/C6H6O6/c7-1-3(6(11)12)5(10)4(9)2-8/h1-3,5,10H,(H,11,12) | 0 | 0 | 1 | 0 | 0 | 0 | 0 |
| 351 | InChI=1S/C6H6O6/c7-1-3(6(11)12)5(10)4(9)2-8/h1-4,9H,(H,11,12) | 0 | 0 | 1 | 0 | 0 | 0 | 0 |
| 352 | InChI=1S/C6H6O6/c7-1-3(2-8)4(9)5(10)6(11)12/h1-3,5,10H,(H,11,12) | 0 | 0 | 1 | 0 | 0 | 0 | 0 |
| 353 | InChI=1S/C6H6O6/c7-1-3(2-8)4(5(9)10)6(11)12/h1-4H,(H,9,10)(H,11,12) | 0 | 0 | 1 | 0 | 0 | 0 | 0 |
| 354 | InChI=1S/C6H6O6/c7-1-3(5(9)10)4(2-8)6(11)12/h1-4H,(H,9,10)(H,11,12) | 0 | 0 | 1 | 0 | 1 | 0 | 0 |
| 355 | InChI=1S/C6H6O6/c1-2(7)3(8)4(9)5(10)6(11)12/h2,7H,1H3,(H,11,12) | 0 | 0 | 1 | 0 | 0 | 0 | 0 |
| 356 | InChI=1S/C6H6O6/c1-2(7)3(8)4(9)5(10)6(11)12/h5,10H,1H3,(H,11,12) | 0 | 0 | 1 | 0 | 0 | 0 | 0 |
| 357 | InChI=1S/C6H6O6/c1-2(7)3(8)4(9)5(10)6(11)12/h3,8H,1H3,(H,11,12) | 0 | 0 | 1 | 0 | 0 | 0 | 0 |
| 358 | InChI=1S/C6H6O6/c1-2(5(9)10)3(7)4(8)6(11)12/h2H,1H3,(H,9,10)(H,11,12) | 0 | 1 | 1 | 0 | 1 | 0 | 0 |
| 359 | InChI=1S/C6H6O6/c1-2(7)3(8)4(9)5(10)6(11)12/h4,9H,1H3,(H,11,12) | 0 | 0 | 1 | 0 | 0 | 0 | 0 |
| 360 | InChI=1S/C6H6O6/c1-2(7)4(8)3(5(9)10)6(11)12/h3H,1H3,(H,9,10)(H,11,12) | 0 | 0 | 1 | 0 | 0 | 0 | 0 |
| 361 | InChI=1S/C6H6O6/c1-2(3(7)5(9)10)4(8)6(11)12/h2H,1H3,(H,9,10)(H,11,12) | 0 | 0 | 1 | 0 | 1 | 0 | 0 |
| 362 | InChI=1S/C6H6O6/c1-2(7)3(5(9)10)4(8)6(11)12/h3H,1H3,(H,9,10)(H,11,12) | 0 | 0 | 1 | 0 | 1 | 0 | 0 |
| 363 | InChI=1S/C6H6O6/c7-1-4(10)6(12,3-9)5(11)2-8/h1-4,10,12H | 0 | 0 | 1 | 0 | 0 | 0 | 0 |
| 364 | InChI=1S/C6H6O6/c7-1-4(10)5(11)6(12,2-8)3-9/h1-4,10,12H | 0 | 0 | 1 | 0 | 0 | 0 | 0 |
| 365 | InChI=1S/C6H6O6/c7-1-4(10)6(2-8,3-9)5(11)12/h1-4,10H,(H,11,12) | 0 | 0 | 1 | 0 | 0 | 0 | 0 |
| 366 | InChI=1S/C6H6O6/c7-1-4(2-8)6(12,3-9)5(10)11/h1-4,12H,(H,10,11) | 0 | 0 | 1 | 0 | 0 | 0 | 0 |
| 367 | InChI=1S/C6H6O6/c7-1-4(10)5(11)6(12,2-8)3-9/h1-3,5,11-12H | 0 | 0 | 1 | 0 | 0 | 0 | 0 |
| 368 | InChI=1S/C6H6O6/c7-1-6(2-8,3-9)4(10)5(11)12/h1-4,10H,(H,11,12) | 0 | 0 | 1 | 0 | 0 | 0 | 0 |
| 369 | InChI=1S/C6H6O6/c7-1-4(5(10)11)6(12,2-8)3-9/h1-4,12H,(H,10,11) | 0 | 0 | 1 | 0 | 0 | 0 | 0 |
| 370 | InChI=1S/C6H6O6/c7-1-5(11,2-8)6(12,3-9)4-10/h1-4,11-12H | 0 | 0 | 1 | 0 | 0 | 0 | 0 |
| 371 | InChI=1S/C6H6O6/c7-1-4(10)5(11)6(12,2-8)3-9/h1-2,9,12H,3H2 | 0 | 0 | 1 | 0 | 0 | 0 | 0 |
| 372 | InChI=1S/C6H6O6/c7-1-4(10)5(11)6(12,2-8)3-9/h2-3,7,12H,1H2 | 0 | 0 | 1 | 0 | 0 | 0 | 0 |
| 373 | InChI=1S/C6H6O6/c7-1-6(2-8,3-9)4(10)5(11)12/h1-2,9H,3H2,(H,11,12) | 0 | 0 | 1 | 0 | 0 | 0 | 0 |
| 374 | InChI=1S/C6H6O6/c7-2-1-6(12,3-8)4(9)5(10)11/h2-3,12H,1H2,(H,10,11) | 0 | 0 | 1 | 0 | 0 | 0 | 0 |
| 375 | InChI=1S/C6H6O6/c7-2-6(12,3-8)1-4(9)5(10)11/h2-3,12H,1H2,(H,10,11) | 0 | 0 | 1 | 0 | 0 | 0 | 0 |
| 376 | InChI=1S/C6H6O6/c7-1-4(10)6(12,3-9)5(11)2-8/h1-2,9,12H,3H2 | 0 | 0 | 1 | 0 | 0 | 0 | 0 |
| 377 | InChI=1S/C6H6O6/c7-1-4(10)6(12,3-9)5(11)2-8/h1,3,8,12H,2H2 | 0 | 0 | 1 | 0 | 0 | 0 | 0 |
| 378 | InChI=1S/C6H6O6/c7-1-4(10)6(2-8,3-9)5(11)12/h1-2,9H,3H2,(H,11,12) | 0 | 0 | 1 | 0 | 0 | 0 | 0 |
| 379 | InChI=1S/C6H6O6/c7-2-1-6(12,5(10)11)4(9)3-8/h2-3,12H,1H2,(H,10,11) | 0 | 0 | 1 | 0 | 0 | 0 | 0 |
| 380 | InChI=1S/C6H6O6/c7-2-4(9)6(12,3-8)1-5(10)11/h2-3,12H,1H2,(H,10,11) | 0 | 0 | 1 | 0 | 0 | 0 | 0 |
| 381 | InChI=1S/C6H6O6/c7-1-4(10)6(2-8,3-9)5(11)12/h2-3,7H,1H2,(H,11,12) | 0 | 0 | 1 | 0 | 0 | 0 | 0 |
| 382 | InChI=1S/C6H6O6/c7-2-1-4(9)6(12,3-8)5(10)11/h2-3,12H,1H2,(H,10,11) | 0 | 0 | 1 | 0 | 0 | 0 | 0 |
| 383 | InChI=1S/C6H6O6/c7-2-6(12,3-8)4(9)1-5(10)11/h2-3,12H,1H2,(H,10,11) | 0 | 0 | 1 | 0 | 0 | 0 | 0 |
| 384 | InChI=1S/C6H6O6/c7-2-1-6(3-8,4(9)10)5(11)12/h2-3H,1H2,(H,9,10)(H,11,12) | 0 | 0 | 1 | 0 | 0 | 0 | 0 |
| 385 | InChI=1S/C6H6O6/c7-2-4(9)1-6(12,3-8)5(10)11/h2-3,12H,1H2,(H,10,11) | 0 | 0 | 1 | 0 | 0 | 0 | 0 |
| 386 | InChI=1S/C6H6O6/c7-2-6(3-8,5(11)12)1-4(9)10/h2-3H,1H2,(H,9,10)(H,11,12) | 0 | 0 | 1 | 0 | 0 | 0 | 0 |
| 387 | InChI=1S/C6H6O6/c1-6(12,2-7)4(9)3(8)5(10)11/h2,12H,1H3,(H,10,11) | 0 | 0 | 1 | 0 | 0 | 0 | 0 |
| 388 | InChI=1S/C6H6O6/c1-6(12,5(10)11)4(9)3(8)2-7/h2,12H,1H3,(H,10,11) | 0 | 0 | 1 | 0 | 0 | 0 | 0 |
| 389 | InChI=1S/C6H6O6/c1-3(8)4(9)6(12,2-7)5(10)11/h2,12H,1H3,(H,10,11) | 0 | 0 | 1 | 0 | 0 | 0 | 0 |
| 390 | InChI=1S/C6H6O6/c1-6(12,3(8)2-7)4(9)5(10)11/h2,12H,1H3,(H,10,11) | 0 | 0 | 1 | 0 | 0 | 0 | 0 |
| 391 | InChI=1S/C6H6O6/c1-3(8)6(12,2-7)4(9)5(10)11/h2,12H,1H3,(H,10,11) | 0 | 0 | 1 | 0 | 0 | 0 | 0 |
| 392 | InChI=1S/C6H6O6/c1-6(2-7,5(11)12)3(8)4(9)10/h2H,1H3,(H,9,10)(H,11,12) | 0 | 0 | 1 | 0 | 0 | 0 | 0 |
| 393 | InChI=1S/C6H6O6/c1-3(8)6(12,5(10)11)4(9)2-7/h2,12H,1H3,(H,10,11) | 0 | 0 | 1 | 0 | 0 | 0 | 0 |
| 394 | InChI=1S/C6H6O6/c1-6(4(9)10,5(11)12)3(8)2-7/h2H,1H3,(H,9,10)(H,11,12) | 0 | 0 | 1 | 0 | 0 | 0 | 0 |
| 395 | InChI=1S/C6H6O6/c1-3(8)6(2-7,4(9)10)5(11)12/h2H,1H3,(H,9,10)(H,11,12) | 0 | 0 | 1 | 0 | 0 | 0 | 0 |
| 396 | InChI=1S/C6H6O7/c7-1-2(4(8)9)3(5(10)11)6(12)13/h7H,1H2,(H,8,9)(H,10,11)(H,12,13) | 0 | 0 | 1 | 0 | 0 | 0 | 0 |
| 397 | InChI=1S/C6H6O7/c7-3(6(12)13)1-2(4(8)9)5(10)11/h1,3,7H,(H,8,9)(H,10,11)(H,12,13) | 0 | 0 | 1 | 0 | 0 | 0 | 0 |
| 398 | InChI=1S/C6H6O7/c7-3(8)1-2(5(10)11)4(9)6(12)13/h1,4,9H,(H,7,8)(H,10,11)(H,12,13) | 0 | 1 | 1 | 0 | 1 | 0 | 0 |
| 399 | InChI=1S/C6H6O7/c7-3(8)1-2-6(13,4(9)10)5(11)12/h1-2,13H,(H,7,8)(H,9,10)(H,11,12) | 0 | 0 | 1 | 0 | 0 | 0 | 0 |
| 400 | InChI=1S/C6H6O7/c1-2(3(7)8)6(13,4(9)10)5(11)12/h13H,1H2,(H,7,8)(H,9,10)(H,11,12) | 0 | 0 | 1 | 0 | 0 | 0 | 0 |
| 401 | InChI=1S/C6H6O7/c7-1-3(9)6(13,2-8)4(10)5(11)12/h1-3,9,13H,(H,11,12) | 0 | 0 | 1 | 0 | 0 | 0 | 0 |
| 402 | InChI=1S/C6H6O7/c7-1-6(13,2-8)4(10)3(9)5(11)12/h1-2,4,10,13H,(H,11,12) | 0 | 0 | 1 | 0 | 0 | 0 | 0 |
| 403 | InChI=1S/C6H6O7/c7-1-3(9)6(13,5(11)12)4(10)2-8/h1-3,9,13H,(H,11,12) | 0 | 0 | 1 | 0 | 0 | 0 | 0 |
| 404 | InChI=1S/C6H6O7/c7-1-3(9)6(13,2-8)4(10)5(11)12/h1-2,4,10,13H,(H,11,12) | 0 | 0 | 1 | 0 | 0 | 0 | 0 |
| 405 | InChI=1S/C6H6O7/c7-1-3(9)4(10)6(13,2-8)5(11)12/h1-3,9,13H,(H,11,12) | 0 | 0 | 1 | 0 | 0 | 0 | 0 |
| 406 | InChI=1S/C6H6O7/c7-1-6(13,2-8)4(10)3(9)5(11)12/h1-3,9,13H,(H,11,12) | 0 | 0 | 1 | 0 | 0 | 0 | 0 |
| 407 | InChI=1S/C6H6O7/c7-1-3(9)6(2-8,4(10)11)5(12)13/h1-3,9H,(H,10,11)(H,12,13) | 0 | 0 | 1 | 0 | 0 | 0 | 0 |
| 408 | InChI=1S/C6H6O7/c7-1-3(2-8)6(13,4(9)10)5(11)12/h1-3,13H,(H,9,10)(H,11,12) | 0 | 0 | 1 | 0 | 0 | 0 | 0 |
| 409 | InChI=1S/C6H6O7/c7-1-3(9)4(10)6(13,2-8)5(11)12/h1-2,4,10,13H,(H,11,12) | 0 | 0 | 1 | 0 | 0 | 0 | 0 |
| 410 | InChI=1S/C6H6O7/c7-1-6(2-8,5(12)13)3(9)4(10)11/h1-3,9H,(H,10,11)(H,12,13) | 0 | 0 | 1 | 0 | 0 | 0 | 0 |
| 411 | InChI=1S/C6H6O7/c7-1-3(4(9)10)6(13,2-8)5(11)12/h1-3,13H,(H,9,10)(H,11,12) | 0 | 0 | 1 | 0 | 0 | 0 | 0 |
| 412 | InChI=1S/C6H6O7/c7-1-6(13,2-8)3(4(9)10)5(11)12/h1-3,13H,(H,9,10)(H,11,12) | 0 | 0 | 1 | 0 | 0 | 0 | 0 |
| 413 | InChI=1S/C6H6O7/c7-1-6(13,2-8)4(10)3(9)5(11)12/h1,8,13H,2H2,(H,11,12) | 0 | 0 | 1 | 0 | 0 | 0 | 0 |
| 414 | InChI=1S/C6H6O7/c7-1-3(9)4(10)6(13,2-8)5(11)12/h1,8,13H,2H2,(H,11,12) | 0 | 0 | 1 | 0 | 0 | 0 | 0 |
| 415 | InChI=1S/C6H6O7/c7-1-3(9)4(10)6(13,2-8)5(11)12/h2,7,13H,1H2,(H,11,12) | 0 | 0 | 1 | 0 | 0 | 0 | 0 |
| 416 | InChI=1S/C6H6O7/c7-1-3(9)6(13,2-8)4(10)5(11)12/h1,8,13H,2H2,(H,11,12) | 0 | 0 | 1 | 0 | 0 | 0 | 0 |
| 417 | InChI=1S/C6H6O7/c7-1-3(9)6(13,2-8)4(10)5(11)12/h2,7,13H,1H2,(H,11,12) | 0 | 0 | 1 | 0 | 0 | 0 | 0 |
| 418 | InChI=1S/C6H6O7/c7-1-6(2-8,5(12)13)3(9)4(10)11/h1,8H,2H2,(H,10,11)(H,12,13) | 0 | 0 | 1 | 0 | 0 | 0 | 0 |
| 419 | InChI=1S/C6H6O7/c7-2-1-6(13,5(11)12)3(8)4(9)10/h2,13H,1H2,(H,9,10)(H,11,12) | 0 | 0 | 1 | 0 | 0 | 0 | 0 |
| 420 | InChI=1S/C6H6O7/c7-2-6(13,1-3(8)9)4(10)5(11)12/h2,13H,1H2,(H,8,9)(H,11,12) | 0 | 0 | 1 | 0 | 0 | 0 | 0 |
| 421 | InChI=1S/C6H6O7/c7-2-6(13,5(11)12)1-3(8)4(9)10/h2,13H,1H2,(H,9,10)(H,11,12) | 0 | 0 | 1 | 0 | 0 | 0 | 0 |
| 422 | InChI=1S/C6H6O7/c7-1-3(9)6(13,5(11)12)4(10)2-8/h1,8,13H,2H2,(H,11,12) | 0 | 0 | 1 | 0 | 0 | 0 | 0 |
| 423 | InChI=1S/C6H6O7/c7-1-3(9)6(2-8,4(10)11)5(12)13/h1,8H,2H2,(H,10,11)(H,12,13) | 0 | 0 | 1 | 0 | 0 | 0 | 0 |
| 424 | InChI=1S/C6H6O7/c7-2-3(8)6(13,5(11)12)1-4(9)10/h2,13H,1H2,(H,9,10)(H,11,12) | 0 | 0 | 1 | 0 | 0 | 0 | 0 |
| 425 | InChI=1S/C6H6O7/c7-1-3(9)6(2-8,4(10)11)5(12)13/h2,7H,1H2,(H,10,11)(H,12,13) | 0 | 0 | 1 | 0 | 0 | 0 | 0 |
| 426 | InChI=1S/C6H6O7/c7-2-1-3(8)6(13,4(9)10)5(11)12/h2,13H,1H2,(H,9,10)(H,11,12) | 0 | 0 | 1 | 0 | 0 | 0 | 0 |
| 427 | InChI=1S/C6H6O7/c7-2-6(13,5(11)12)3(8)1-4(9)10/h2,13H,1H2,(H,9,10)(H,11,12) | 0 | 0 | 1 | 0 | 0 | 0 | 0 |
| 428 | InChI=1S/C6H6O7/c7-2-1-6(3(8)9,4(10)11)5(12)13/h2H,1H2,(H,8,9)(H,10,11)(H,12,13) | 0 | 0 | 1 | 0 | 0 | 0 | 0 |
| 429 | InChI=1S/C6H6O7/c7-2-3(8)1-6(13,4(9)10)5(11)12/h2,13H,1H2,(H,9,10)(H,11,12) | 0 | 0 | 1 | 0 | 0 | 0 | 0 |
| 430 | InChI=1S/C6H6O7/c7-2-6(4(10)11,5(12)13)1-3(8)9/h2H,1H2,(H,8,9)(H,10,11)(H,12,13) | 0 | 0 | 1 | 0 | 0 | 0 | 0 |
| 431 | InChI=1S/C6H6O7/c1-6(13,5(11)12)3(8)2(7)4(9)10/h13H,1H3,(H,9,10)(H,11,12) | 0 | 0 | 1 | 0 | 0 | 0 | 0 |
| 432 | InChI=1S/C6H6O7/c1-2(7)3(8)6(13,4(9)10)5(11)12/h13H,1H3,(H,9,10)(H,11,12) | 0 | 0 | 1 | 0 | 0 | 0 | 0 |
| 433 | InChI=1S/C6H6O7/c1-6(13,2(7)4(9)10)3(8)5(11)12/h13H,1H3,(H,9,10)(H,11,12) | 0 | 0 | 1 | 0 | 0 | 0 | 0 |
| 434 | InChI=1S/C6H6O7/c1-2(7)6(13,5(11)12)3(8)4(9)10/h13H,1H3,(H,9,10)(H,11,12) | 0 | 0 | 1 | 0 | 0 | 0 | 0 |
| 435 | InChI=1S/C6H6O7/c1-6(4(10)11,5(12)13)2(7)3(8)9/h1H3,(H,8,9)(H,10,11)(H,12,13) | 0 | 0 | 1 | 0 | 1 | 0 | 0 |
| 436 | InChI=1S/C6H6O7/c1-2(7)6(3(8)9,4(10)11)5(12)13/h1H3,(H,8,9)(H,10,11)(H,12,13) | 0 | 0 | 1 | 0 | 1 | 0 | 0 |
| 437 | InChI=1S/C6H6O7/c7-1-5(12,2-8)6(13,3-9)4(10)11/h1-3,12-13H,(H,10,11) | 0 | 0 | 1 | 0 | 0 | 0 | 0 |
| 438 | InChI=1S/C6H6O7/c7-1-2(8)3(9)4(10)5(11)6(12)13/h1-3,8-9H,(H,12,13) | 0 | 0 | 1 | 0 | 0 | 0 | 0 |
| 439 | InChI=1S/C6H6O7/c7-1-2(8)3(9)4(10)5(11)6(12)13/h1,4-5,10-11H,(H,12,13) | 0 | 0 | 1 | 0 | 0 | 0 | 0 |
| 440 | InChI=1S/C6H6O7/c7-1-2(8)3(9)4(10)5(11)6(12)13/h1-2,5,8,11H,(H,12,13) | 0 | 0 | 1 | 0 | 0 | 0 | 0 |
| 441 | InChI=1S/C6H6O7/c7-1-2(8)3(9)4(10)5(11)6(12)13/h1,3-4,9-10H,(H,12,13) | 0 | 0 | 1 | 0 | 0 | 0 | 0 |
| 442 | InChI=1S/C6H6O7/c7-1-2(8)3(9)4(10)5(11)6(12)13/h1-2,4,8,10H,(H,12,13) | 0 | 0 | 1 | 0 | 0 | 0 | 0 |
| 443 | InChI=1S/C6H6O7/c7-1-2(8)3(5(10)11)4(9)6(12)13/h1-3,8H,(H,10,11)(H,12,13) | 0 | 0 | 1 | 0 | 0 | 0 | 0 |
| 444 | InChI=1S/C6H6O7/c7-1-2(3(8)5(10)11)4(9)6(12)13/h1-3,8H,(H,10,11)(H,12,13) | 0 | 0 | 1 | 0 | 0 | 0 | 0 |
| 445 | InChI=1S/C6H6O7/c7-1-2(5(10)11)3(8)4(9)6(12)13/h1-3,8H,(H,10,11)(H,12,13) | 0 | 0 | 1 | 0 | 0 | 0 | 0 |
| 446 | InChI=1S/C6H6O7/c7-1-2(8)3(9)4(10)5(11)6(12)13/h1,3,5,9,11H,(H,12,13) | 0 | 0 | 1 | 0 | 0 | 0 | 0 |
| 447 | InChI=1S/C6H6O7/c7-1-2(8)3(5(10)11)4(9)6(12)13/h1,3-4,9H,(H,10,11)(H,12,13) | 0 | 0 | 1 | 0 | 0 | 0 | 0 |
| 448 | InChI=1S/C6H6O7/c7-1-2(8)4(9)3(5(10)11)6(12)13/h1,3-4,9H,(H,10,11)(H,12,13) | 0 | 0 | 1 | 0 | 0 | 0 | 0 |
| 449 | InChI=1S/C6H6O7/c7-1-2(8)4(9)3(5(10)11)6(12)13/h1-3,8H,(H,10,11)(H,12,13) | 0 | 0 | 1 | 0 | 0 | 0 | 0 |
| 450 | InChI=1S/C6H6O7/c7-1-2(5(10)11)3(8)4(9)6(12)13/h1-2,4,9H,(H,10,11)(H,12,13) | 0 | 0 | 1 | 0 | 0 | 0 | 0 |
| 451 | InChI=1S/C6H6O7/c7-1-2(4(8)9)3(5(10)11)6(12)13/h1-3H,(H,8,9)(H,10,11)(H,12,13) | 0 | 0 | 1 | 0 | 1 | 0 | 0 |
| 452 | InChI=1S/C6H6O7/c7-1-2(8)3(9)4(10)5(11)6(12)13/h2,7-8H,1H2,(H,12,13) | 0 | 0 | 1 | 0 | 0 | 0 | 0 |
| 453 | InChI=1S/C6H6O7/c7-1-2(8)3(9)4(10)5(11)6(12)13/h5,7,11H,1H2,(H,12,13) | 0 | 0 | 1 | 0 | 0 | 0 | 0 |
| 454 | InChI=1S/C6H6O7/c7-1-2(8)3(9)4(10)5(11)6(12)13/h3,7,9H,1H2,(H,12,13) | 0 | 0 | 1 | 0 | 0 | 0 | 0 |
| 455 | InChI=1S/C6H6O7/c7-1-2(5(10)11)3(8)4(9)6(12)13/h2,7H,1H2,(H,10,11)(H,12,13) | 0 | 0 | 1 | 0 | 0 | 0 | 0 |
| 456 | InChI=1S/C6H6O7/c7-2(1-3(8)9)4(10)5(11)6(12)13/h2,7H,1H2,(H,8,9)(H,12,13) | 0 | 0 | 1 | 0 | 0 | 0 | 0 |
| 457 | InChI=1S/C6H6O7/c7-2(4(9)6(12)13)1-3(8)5(10)11/h3,8H,1H2,(H,10,11)(H,12,13) | 0 | 0 | 1 | 0 | 0 | 0 | 0 |
| 458 | InChI=1S/C6H6O7/c7-1-2(8)3(9)4(10)5(11)6(12)13/h4,7,10H,1H2,(H,12,13) | 0 | 0 | 1 | 0 | 0 | 0 | 0 |
| 459 | InChI=1S/C6H6O7/c7-1-2(8)4(9)3(5(10)11)6(12)13/h3,7H,1H2,(H,10,11)(H,12,13) | 0 | 0 | 1 | 0 | 0 | 0 | 0 |
| 460 | InChI=1S/C6H6O7/c7-2(1-3(8)9)4(10)5(11)6(12)13/h5,11H,1H2,(H,8,9)(H,12,13) | 0 | 0 | 1 | 0 | 0 | 0 | 0 |
| 461 | InChI=1S/C6H6O7/c7-1-2(3(8)5(10)11)4(9)6(12)13/h2,7H,1H2,(H,10,11)(H,12,13) | 0 | 0 | 1 | 0 | 0 | 0 | 0 |
| 462 | InChI=1S/C6H6O7/c7-2(4(9)6(12)13)1-3(8)5(10)11/h2,7H,1H2,(H,10,11)(H,12,13) | 0 | 0 | 1 | 0 | 0 | 0 | 0 |
| 463 | InChI=1S/C6H6O7/c7-1-2(8)3(5(10)11)4(9)6(12)13/h3,7H,1H2,(H,10,11)(H,12,13) | 0 | 0 | 1 | 0 | 0 | 0 | 0 |
| 464 | InChI=1S/C6H6O7/c7-2(1-3(8)9)4(10)5(11)6(12)13/h4,10H,1H2,(H,8,9)(H,12,13) | 0 | 0 | 1 | 0 | 0 | 0 | 0 |
| 465 | InChI=1S/C6H6O7/c7-3(8)1-2(5(10)11)4(9)6(12)13/h2H,1H2,(H,7,8)(H,10,11)(H,12,13) | 1 | 1 | 1 | 0 | 1 | 1 | 1 |
| 466 | InChI=1S/C6H6O7/c7-2(4(9)6(12)13)1-3(8)5(10)11/h4,9H,1H2,(H,10,11)(H,12,13) | 0 | 0 | 1 | 0 | 0 | 0 | 0 |
| 467 | InChI=1S/C6H6O7/c7-3(6(12)13)1-2(4(8)9)5(10)11/h2H,1H2,(H,8,9)(H,10,11)(H,12,13) | 0 | 0 | 1 | 0 | 0 | 0 | 0 |
| 468 | InChI=1S/C6H6O7/c7-2(1-3(8)9)4(5(10)11)6(12)13/h4H,1H2,(H,8,9)(H,10,11)(H,12,13) | 0 | 0 | 1 | 0 | 1 | 0 | 0 |
| 469 | InChI=1S/C6H6O8/c7-1-2(8)6(14,5(12)13)3(9)4(10)11/h1-2,8,14H,(H,10,11)(H,12,13) | 0 | 0 | 1 | 0 | 0 | 0 | 0 |
| 470 | InChI=1S/C6H6O8/c7-1-6(14,2(8)4(10)11)3(9)5(12)13/h1-2,8,14H,(H,10,11)(H,12,13) | 0 | 0 | 1 | 0 | 0 | 0 | 0 |
| 471 | InChI=1S/C6H6O8/c7-1-6(14,5(12)13)3(9)2(8)4(10)11/h1,3,9,14H,(H,10,11)(H,12,13) | 0 | 0 | 1 | 0 | 0 | 0 | 0 |
| 472 | InChI=1S/C6H6O8/c7-1-2(8)6(14,5(12)13)3(9)4(10)11/h1,3,9,14H,(H,10,11)(H,12,13) | 0 | 0 | 1 | 0 | 0 | 0 | 0 |
| 473 | InChI=1S/C6H6O8/c7-1-2(8)3(9)6(14,4(10)11)5(12)13/h1-2,8,14H,(H,10,11)(H,12,13) | 0 | 0 | 1 | 0 | 0 | 0 | 0 |
| 474 | InChI=1S/C6H6O8/c7-1-6(14,5(12)13)3(9)2(8)4(10)11/h1-2,8,14H,(H,10,11)(H,12,13) | 0 | 0 | 1 | 0 | 0 | 0 | 0 |
| 475 | InChI=1S/C6H6O8/c7-1-2(8)6(3(9)10,4(11)12)5(13)14/h1-2,8H,(H,9,10)(H,11,12)(H,13,14) | 0 | 0 | 1 | 0 | 0 | 0 | 0 |
| 476 | InChI=1S/C6H6O8/c7-1-2(8)3(9)6(14,4(10)11)5(12)13/h1,3,9,14H,(H,10,11)(H,12,13) | 0 | 0 | 1 | 0 | 0 | 0 | 0 |
| 477 | InChI=1S/C6H6O8/c7-1-6(4(11)12,5(13)14)2(8)3(9)10/h1-2,8H,(H,9,10)(H,11,12)(H,13,14) | 0 | 0 | 1 | 0 | 0 | 0 | 0 |
| 478 | InChI=1S/C6H6O8/c7-1-2(3(8)9)6(14,4(10)11)5(12)13/h1-2,14H,(H,8,9)(H,10,11)(H,12,13) | 0 | 0 | 1 | 0 | 0 | 0 | 0 |
| 479 | InChI=1S/C6H6O8/c7-1-6(14,5(12)13)2(3(8)9)4(10)11/h1-2,14H,(H,8,9)(H,10,11)(H,12,13) | 0 | 0 | 1 | 0 | 0 | 0 | 0 |
| 480 | InChI=1S/C6H6O8/c7-1-5(13,2-8)6(14,3(9)10)4(11)12/h1-2,13-14H,(H,9,10)(H,11,12) | 0 | 0 | 1 | 0 | 0 | 0 | 0 |
| 481 | InChI=1S/C6H6O8/c7-1-5(13,3(9)10)6(14,2-8)4(11)12/h1-2,13-14H,(H,9,10)(H,11,12) | 0 | 0 | 1 | 0 | 0 | 0 | 0 |
| 482 | InChI=1S/C6H6O8/c7-1-6(14,5(12)13)3(9)2(8)4(10)11/h7,14H,1H2,(H,10,11)(H,12,13) | 0 | 0 | 1 | 0 | 0 | 0 | 0 |
| 483 | InChI=1S/C6H6O8/c7-1-2(8)3(9)6(14,4(10)11)5(12)13/h7,14H,1H2,(H,10,11)(H,12,13) | 0 | 0 | 1 | 0 | 0 | 0 | 0 |
| 484 | InChI=1S/C6H6O8/c7-1-6(14,2(8)4(10)11)3(9)5(12)13/h7,14H,1H2,(H,10,11)(H,12,13) | 0 | 0 | 1 | 0 | 0 | 0 | 0 |
| 485 | InChI=1S/C6H6O8/c7-1-2(8)6(14,5(12)13)3(9)4(10)11/h7,14H,1H2,(H,10,11)(H,12,13) | 0 | 0 | 1 | 0 | 0 | 0 | 0 |
| 486 | InChI=1S/C6H6O8/c7-1-6(4(11)12,5(13)14)2(8)3(9)10/h7H,1H2,(H,9,10)(H,11,12)(H,13,14) | 0 | 0 | 1 | 0 | 0 | 0 | 0 |
| 487 | InChI=1S/C6H6O8/c7-2(8)1-6(14,5(12)13)3(9)4(10)11/h14H,1H2,(H,7,8)(H,10,11)(H,12,13) | 0 | 1 | 1 | 0 | 1 | 0 | 0 |
| 488 | InChI=1S/C6H6O8/c7-2(3(8)9)1-6(14,4(10)11)5(12)13/h14H,1H2,(H,8,9)(H,10,11)(H,12,13) | 0 | 0 | 1 | 0 | 0 | 0 | 0 |
| 489 | InChI=1S/C6H6O8/c7-1-2(8)6(3(9)10,4(11)12)5(13)14/h7H,1H2,(H,9,10)(H,11,12)(H,13,14) | 0 | 0 | 1 | 0 | 0 | 0 | 0 |
| 490 | InChI=1S/C6H6O8/c7-2(1-3(8)9)6(14,4(10)11)5(12)13/h14H,1H2,(H,8,9)(H,10,11)(H,12,13) | 0 | 0 | 1 | 0 | 0 | 0 | 0 |
| 491 | InChI=1S/C6H6O8/c7-2(8)1-6(3(9)10,4(11)12)5(13)14/h1H2,(H,7,8)(H,9,10)(H,11,12)(H,13,14) | 0 | 1 | 1 | 0 | 1 | 0 | 0 |
| 492 | InChI=1S/C6H6O8/c7-1(3(9)5(11)12)2(8)4(10)6(13)14/h1,3,7,9H,(H,11,12)(H,13,14) | 0 | 0 | 1 | 0 | 0 | 0 | 0 |
| 493 | InChI=1S/C6H6O8/c7-1(3(9)5(11)12)2(8)4(10)6(13)14/h3-4,9-10H,(H,11,12)(H,13,14) | 0 | 0 | 1 | 0 | 0 | 0 | 0 |
| 494 | InChI=1S/C6H6O8/c7-1(3(9)5(11)12)2(8)4(10)6(13)14/h1-2,7-8H,(H,11,12)(H,13,14) | 1 | 0 | 1 | 0 | 0 | 1 | 0 |
| 495 | InChI=1S/C6H6O8/c7-1(3(9)5(11)12)2(8)4(10)6(13)14/h1,4,7,10H,(H,11,12)(H,13,14) | 0 | 0 | 1 | 0 | 0 | 0 | 0 |
| 496 | InChI=1S/C6H6O8/c7-2(5(11)12)1(4(9)10)3(8)6(13)14/h1-2,7H,(H,9,10)(H,11,12)(H,13,14) | 1 | 1 | 1 | 1 | 1 | 1 | 0 |
| 497 | InChI=1S/C6H6O8/c7-2(3(8)6(13)14)1(4(9)10)5(11)12/h1-2,7H,(H,9,10)(H,11,12)(H,13,14) | 0 | 0 | 1 | 0 | 0 | 0 | 0 |
| 498 | InChI=1S/C6H6O8/c7-2(3(8)6(13)14)1(4(9)10)5(11)12/h1,3,8H,(H,9,10)(H,11,12)(H,13,14) | 0 | 0 | 1 | 0 | 0 | 0 | 0 |
| 499 | InChI=1S/C6H6O8/c7-3(8)1(4(9)10)2(5(11)12)6(13)14/h1-2H,(H,7,8)(H,9,10)(H,11,12)(H,13,14) | 1 | 1 | 1 | 0 | 1 | 1 | 0 |
| 500 | InChI=1S/C6H6O9/c7-1-5(14,2(8)9)6(15,3(10)11)4(12)13/h1,14-15H,(H,8,9)(H,10,11)(H,12,13) | 0 | 0 | 1 | 0 | 0 | 0 | 0 |
| 501 | InChI=1S/C6H6O9/c7-1(3(9)10)6(15,5(13)14)2(8)4(11)12/h1,7,15H,(H,9,10)(H,11,12)(H,13,14) | 0 | 0 | 1 | 0 | 0 | 0 | 0 |
| 502 | InChI=1S/C6H6O9/c7-1(3(9)10)2(8)6(15,4(11)12)5(13)14/h2,8,15H,(H,9,10)(H,11,12)(H,13,14) | 0 | 0 | 1 | 0 | 0 | 0 | 0 |
| 503 | InChI=1S/C6H6O9/c7-1(3(9)10)2(8)6(15,4(11)12)5(13)14/h1,7,15H,(H,9,10)(H,11,12)(H,13,14) | 0 | 0 | 1 | 0 | 0 | 0 | 0 |
| 504 | InChI=1S/C6H6O9/c7-1(2(8)9)6(3(10)11,4(12)13)5(14)15/h1,7H,(H,8,9)(H,10,11)(H,12,13)(H,14,15) | 0 | 0 | 1 | 0 | 0 | 0 | 0 |
| 505 | InChI=1S/C6H6O9/c7-2(8)1(3(9)10)6(15,4(11)12)5(13)14/h1,15H,(H,7,8)(H,9,10)(H,11,12)(H,13,14) | 0 | 0 | 1 | 0 | 1 | 0 | 0 |
| 506 | InChI=1S/C6H6O10/c7-1(8)5(15,2(9)10)6(16,3(11)12)4(13)14/h15-16H,(H,7,8)(H,9,10)(H,11,12)(H,13,14) | 0 | 0 | 1 | 0 | 0 | 0 | 0 |
| 507 | InChI=1S/C6H8O6/c7-1-3(2-8)4(5(9)10)6(11)12/h7-8H,1-2H2,(H,9,10)(H,11,12) | 0 | 0 | 1 | 0 | 0 | 0 | 0 |
| 508 | InChI=1S/C6H8O6/c7-1-3(5(9)10)4(2-8)6(11)12/h7-8H,1-2H2,(H,9,10)(H,11,12) | 0 | 0 | 1 | 0 | 1 | 0 | 0 |
| 509 | InChI=1S/C6H8O6/c7-2-3(8)1-4(5(9)10)6(11)12/h1,3,7-8H,2H2,(H,9,10)(H,11,12) | 0 | 0 | 1 | 0 | 0 | 0 | 0 |
| 510 | InChI=1S/C6H8O6/c7-2-4(8)3(6(11)12)1-5(9)10/h1,4,7-8H,2H2,(H,9,10)(H,11,12) | 0 | 0 | 1 | 0 | 0 | 0 | 0 |
| 511 | InChI=1S/C6H8O6/c7-2-1-3(5(9)10)4(8)6(11)12/h1,4,7-8H,2H2,(H,9,10)(H,11,12) | 0 | 0 | 1 | 0 | 0 | 0 | 0 |
| 512 | InChI=1S/C6H8O6/c7-2-3(5(9)10)1-4(8)6(11)12/h1,4,7-8H,2H2,(H,9,10)(H,11,12) | 0 | 0 | 1 | 0 | 0 | 0 | 0 |
| 513 | InChI=1S/C6H8O6/c7-2-3(1-4(8)9)5(10)6(11)12/h1,5,7,10H,2H2,(H,8,9)(H,11,12) | 0 | 0 | 1 | 0 | 0 | 0 | 0 |
| 514 | InChI=1S/C6H8O6/c7-3(1-2-4(8)9)5(10)6(11)12/h1-3,5,7,10H,(H,8,9)(H,11,12) | 0 | 0 | 1 | 0 | 0 | 0 | 0 |
| 515 | InChI=1S/C6H8O6/c7-3(5(9)10)1-2-4(8)6(11)12/h1-4,7-8H,(H,9,10)(H,11,12) | 0 | 0 | 1 | 0 | 1 | 0 | 0 |
| 516 | InChI=1S/C6H8O6/c1-2(5(9)10)3(7)4(8)6(11)12/h3-4,7-8H,1H2,(H,9,10)(H,11,12) | 0 | 0 | 1 | 0 | 0 | 0 | 0 |
| 517 | InChI=1S/C6H8O6/c1-2(3(7)5(9)10)4(8)6(11)12/h3-4,7-8H,1H2,(H,9,10)(H,11,12) | 0 | 0 | 1 | 0 | 1 | 0 | 0 |
| 518 | InChI=1S/C6H8O6/c7-3-1-2-6(12,4(8)9)5(10)11/h1-2,7,12H,3H2,(H,8,9)(H,10,11) | 0 | 0 | 1 | 0 | 1 | 0 | 0 |
| 519 | InChI=1S/C6H8O6/c7-3-6(12,5(10)11)2-1-4(8)9/h1-2,7,12H,3H2,(H,8,9)(H,10,11) | 0 | 0 | 1 | 0 | 1 | 0 | 0 |
| 520 | InChI=1S/C6H8O6/c1-2-3(7)6(12,4(8)9)5(10)11/h2-3,7,12H,1H2,(H,8,9)(H,10,11) | 0 | 0 | 1 | 0 | 0 | 0 | 0 |
| 521 | InChI=1S/C6H8O6/c1-2-6(12,5(10)11)3(7)4(8)9/h2-3,7,12H,1H2,(H,8,9)(H,10,11) | 0 | 0 | 1 | 0 | 0 | 0 | 0 |
| 522 | InChI=1S/C6H8O6/c1-3(4(8)9)6(12,2-7)5(10)11/h7,12H,1-2H2,(H,8,9)(H,10,11) | 0 | 0 | 1 | 0 | 0 | 0 | 0 |
| 523 | InChI=1S/C6H8O6/c1-3(2-7)6(12,4(8)9)5(10)11/h7,12H,1-2H2,(H,8,9)(H,10,11) | 0 | 0 | 1 | 0 | 1 | 0 | 0 |
| 524 | InChI=1S/C6H8O6/c7-1-4(10)6(12,3-9)5(11)2-8/h1,3,5,8,11-12H,2H2 | 0 | 0 | 1 | 0 | 0 | 0 | 0 |
| 525 | InChI=1S/C6H8O6/c7-1-4(10)6(12,3-9)5(11)2-8/h1-2,4,9-10,12H,3H2 | 0 | 0 | 1 | 0 | 0 | 0 | 0 |
| 526 | InChI=1S/C6H8O6/c7-1-4(10)5(11)6(12,2-8)3-9/h2-4,7,10,12H,1H2 | 0 | 0 | 1 | 0 | 0 | 0 | 0 |
| 527 | InChI=1S/C6H8O6/c7-1-4(10)5(11)6(12,2-8)3-9/h1-2,4,9-10,12H,3H2 | 0 | 0 | 1 | 0 | 0 | 0 | 0 |
| 528 | InChI=1S/C6H8O6/c7-1-4(10)6(12,3-9)5(11)2-8/h1,3-4,8,10,12H,2H2 | 0 | 0 | 1 | 0 | 0 | 0 | 0 |
| 529 | InChI=1S/C6H8O6/c7-1-4(10)6(2-8,3-9)5(11)12/h2-4,7,10H,1H2,(H,11,12) | 0 | 0 | 1 | 0 | 0 | 0 | 0 |
| 530 | InChI=1S/C6H8O6/c7-1-4(10)6(2-8,3-9)5(11)12/h1-2,4,9-10H,3H2,(H,11,12) | 0 | 0 | 1 | 0 | 0 | 0 | 0 |
| 531 | InChI=1S/C6H8O6/c7-1-4(2-8)6(12,3-9)5(10)11/h1,3-4,8,12H,2H2,(H,10,11) | 0 | 0 | 1 | 0 | 0 | 0 | 0 |
| 532 | InChI=1S/C6H8O6/c7-2-1-4(9)6(12,3-8)5(10)11/h2-4,9,12H,1H2,(H,10,11) | 0 | 0 | 1 | 0 | 0 | 0 | 0 |
| 533 | InChI=1S/C6H8O6/c7-2-4(9)1-6(12,3-8)5(10)11/h2-4,9,12H,1H2,(H,10,11) | 0 | 0 | 1 | 0 | 0 | 0 | 0 |
| 534 | InChI=1S/C6H8O6/c7-1-4(2-8)6(12,3-9)5(10)11/h1-2,4,9,12H,3H2,(H,10,11) | 0 | 0 | 1 | 0 | 0 | 0 | 0 |
| 535 | InChI=1S/C6H8O6/c7-2-1-6(12,5(10)11)4(9)3-8/h2-4,9,12H,1H2,(H,10,11) | 0 | 0 | 1 | 0 | 0 | 0 | 0 |
| 536 | InChI=1S/C6H8O6/c7-1-4(10)5(11)6(12,2-8)3-9/h1-2,5,9,11-12H,3H2 | 0 | 0 | 1 | 0 | 0 | 0 | 0 |
| 537 | InChI=1S/C6H8O6/c7-1-4(10)5(11)6(12,2-8)3-9/h2-3,5,7,11-12H,1H2 | 0 | 0 | 1 | 0 | 0 | 0 | 0 |
| 538 | InChI=1S/C6H8O6/c7-1-6(2-8,3-9)4(10)5(11)12/h1-2,4,9-10H,3H2,(H,11,12) | 0 | 0 | 1 | 0 | 1 | 0 | 0 |
| 539 | InChI=1S/C6H8O6/c7-1-4(5(10)11)6(12,2-8)3-9/h2-4,7,12H,1H2,(H,10,11) | 0 | 0 | 1 | 0 | 0 | 0 | 0 |
| 540 | InChI=1S/C6H8O6/c7-2-6(12,3-8)1-4(9)5(10)11/h2-4,9,12H,1H2,(H,10,11) | 0 | 0 | 1 | 0 | 0 | 0 | 0 |
| 541 | InChI=1S/C6H8O6/c7-1-4(5(10)11)6(12,2-8)3-9/h1-2,4,9,12H,3H2,(H,10,11) | 0 | 0 | 1 | 0 | 0 | 0 | 0 |
| 542 | InChI=1S/C6H8O6/c7-2-1-6(12,3-8)4(9)5(10)11/h2-4,9,12H,1H2,(H,10,11) | 0 | 0 | 1 | 0 | 0 | 0 | 0 |
| 543 | InChI=1S/C6H8O6/c7-2-6(12,3-8)4(9)1-5(10)11/h2-4,9,12H,1H2,(H,10,11) | 0 | 0 | 1 | 0 | 0 | 0 | 0 |
| 544 | InChI=1S/C6H8O6/c7-2-4(9)6(12,3-8)1-5(10)11/h2-4,9,12H,1H2,(H,10,11) | 0 | 0 | 1 | 0 | 0 | 0 | 0 |
| 545 | InChI=1S/C6H8O6/c7-1-4(10)5(11)6(12,2-8)3-9/h1-5,10-12H | 0 | 0 | 1 | 0 | 0 | 0 | 0 |
| 546 | InChI=1S/C6H8O6/c7-1-4(10)6(12,3-9)5(11)2-8/h1-5,10-12H | 0 | 0 | 1 | 0 | 0 | 0 | 0 |
| 547 | InChI=1S/C6H8O6/c7-1-3(9)5(11)6(12)4(10)2-8/h1-3,5-6,9,11-12H | 0 | 0 | 1 | 0 | 0 | 0 | 0 |
| 548 | InChI=1S/C6H8O6/c7-1-3(9)5(11)6(12)4(10)2-8/h1-5,9-11H | 0 | 0 | 1 | 0 | 0 | 0 | 0 |
| 549 | InChI=1S/C6H8O6/c7-1-3(6(11)12)5(10)4(9)2-8/h1-5,9-10H,(H,11,12) | 0 | 0 | 1 | 0 | 0 | 0 | 0 |
| 550 | InChI=1S/C6H8O6/c7-1-3(2-8)4(9)5(10)6(11)12/h1-5,9-10H,(H,11,12) | 0 | 0 | 1 | 0 | 0 | 0 | 0 |
| 551 | InChI=1S/C6H8O6/c7-1-3(9)5(6(11)12)4(10)2-8/h1-5,9-10H,(H,11,12) | 0 | 0 | 1 | 0 | 0 | 0 | 0 |
| 552 | InChI=1S/C6H8O6/c7-1-3(4(9)2-8)5(10)6(11)12/h1-5,9-10H,(H,11,12) | 0 | 0 | 1 | 0 | 0 | 0 | 0 |
| 553 | InChI=1S/C6H8O6/c7-1-3(9)5(11)6(12)4(10)2-8/h1,4,6,8,10,12H,2H2 | 0 | 0 | 1 | 0 | 1 | 1 | 0 |
| 554 | InChI=1S/C6H8O6/c7-1-3(9)5(11)6(12)4(10)2-8/h1,3-4,8-10H,2H2 | 0 | 0 | 1 | 0 | 0 | 0 | 0 |
| 555 | InChI=1S/C6H8O6/c7-1-3(9)5(11)6(12)4(10)2-8/h1,3,5,8-9,11H,2H2 | 0 | 0 | 1 | 0 | 1 | 0 | 0 |
| 556 | InChI=1S/C6H8O6/c7-1-3(4(9)2-8)5(10)6(11)12/h1,3-4,8-9H,2H2,(H,11,12) | 0 | 0 | 1 | 0 | 0 | 0 | 0 |
| 557 | InChI=1S/C6H8O6/c7-1-3(4(9)2-8)5(10)6(11)12/h2-4,7,9H,1H2,(H,11,12) | 0 | 0 | 1 | 0 | 0 | 0 | 0 |
| 558 | InChI=1S/C6H8O6/c7-1-3(2-8)4(9)5(10)6(11)12/h1,3-4,8-9H,2H2,(H,11,12) | 0 | 0 | 1 | 0 | 0 | 0 | 0 |
| 559 | InChI=1S/C6H8O6/c7-2-3(8)1-4(9)5(10)6(11)12/h2-4,8-9H,1H2,(H,11,12) | 0 | 0 | 1 | 0 | 0 | 0 | 0 |
| 560 | InChI=1S/C6H8O6/c7-2-1-3(8)4(9)5(10)6(11)12/h2-4,8-9H,1H2,(H,11,12) | 0 | 0 | 1 | 0 | 1 | 0 | 0 |
| 561 | InChI=1S/C6H8O6/c7-2-5(10)3(8)1-4(9)6(11)12/h2-3,5,8,10H,1H2,(H,11,12) | 0 | 0 | 1 | 0 | 1 | 1 | 0 |
| 562 | InChI=1S/C6H8O6/c7-1-3(9)5(11)6(12)4(10)2-8/h1,4-5,8,10-11H,2H2 | 0 | 0 | 1 | 0 | 0 | 0 | 0 |
| 563 | InChI=1S/C6H8O6/c7-1-3(9)5(6(11)12)4(10)2-8/h1,4-5,8,10H,2H2,(H,11,12) | 0 | 0 | 1 | 0 | 0 | 0 | 0 |
| 564 | InChI=1S/C6H8O6/c7-1-3(9)5(11)6(12)4(10)2-8/h1,5-6,8,11-12H,2H2 | 0 | 0 | 1 | 0 | 1 | 0 | 0 |
| 565 | InChI=1S/C6H8O6/c7-1-3(4(9)2-8)5(10)6(11)12/h2-3,5,7,10H,1H2,(H,11,12) | 0 | 0 | 1 | 0 | 0 | 0 | 0 |
| 566 | InChI=1S/C6H8O6/c7-1-3(6(11)12)5(10)4(9)2-8/h2-3,5,7,10H,1H2,(H,11,12) | 0 | 0 | 1 | 0 | 0 | 0 | 0 |
| 567 | InChI=1S/C6H8O6/c7-2-5(10)3(8)1-4(9)6(11)12/h2-4,8-9H,1H2,(H,11,12) | 0 | 0 | 1 | 0 | 0 | 0 | 0 |
| 568 | InChI=1S/C6H8O6/c7-2-4(9)6(12)3(8)1-5(10)11/h2-3,6,8,12H,1H2,(H,10,11) | 0 | 0 | 1 | 0 | 0 | 0 | 0 |
| 569 | InChI=1S/C6H8O6/c7-2-3(8)1-4(9)5(10)6(11)12/h2,4-5,9-10H,1H2,(H,11,12) | 0 | 0 | 1 | 0 | 0 | 0 | 0 |
| 570 | InChI=1S/C6H8O6/c7-1-3(9)5(11)6(12)4(10)2-8/h1,3,6,8-9,12H,2H2 | 0 | 0 | 1 | 0 | 0 | 0 | 0 |
| 571 | InChI=1S/C6H8O6/c7-1-3(6(11)12)5(10)4(9)2-8/h1,3-4,8-9H,2H2,(H,11,12) | 0 | 0 | 1 | 0 | 0 | 0 | 0 |
| 572 | InChI=1S/C6H8O6/c7-1-3(6(11)12)5(10)4(9)2-8/h2-4,7,9H,1H2,(H,11,12) | 0 | 0 | 1 | 0 | 0 | 0 | 0 |
| 573 | InChI=1S/C6H8O6/c7-1-3(2-8)4(9)5(10)6(11)12/h1,3,5,8,10H,2H2,(H,11,12) | 0 | 0 | 1 | 0 | 0 | 0 | 0 |
| 574 | InChI=1S/C6H8O6/c7-2-1-3(8)4(9)5(10)6(11)12/h2-3,5,8,10H,1H2,(H,11,12) | 0 | 0 | 1 | 0 | 0 | 0 | 0 |
| 575 | InChI=1S/C6H8O6/c7-2-4(9)6(12)3(8)1-5(10)11/h2-4,8-9H,1H2,(H,10,11) | 0 | 0 | 1 | 0 | 0 | 0 | 0 |
| 576 | InChI=1S/C6H8O6/c7-1-3(9)5(6(11)12)4(10)2-8/h1,3,5,8-9H,2H2,(H,11,12) | 0 | 0 | 1 | 0 | 0 | 0 | 0 |
| 577 | InChI=1S/C6H8O6/c7-2-3(8)1-4(9)5(10)6(11)12/h2-3,5,8,10H,1H2,(H,11,12) | 0 | 0 | 1 | 0 | 0 | 0 | 0 |
| 578 | InChI=1S/C6H8O6/c7-1-3(4(9)2-8)5(10)6(11)12/h1,3,5,8,10H,2H2,(H,11,12) | 0 | 0 | 1 | 0 | 0 | 0 | 0 |
| 579 | InChI=1S/C6H8O6/c7-2-5(10)3(8)1-4(9)6(11)12/h2,4-5,9-10H,1H2,(H,11,12) | 0 | 0 | 1 | 0 | 0 | 0 | 0 |
| 580 | InChI=1S/C6H8O6/c7-1-3(6(11)12)5(10)4(9)2-8/h1,3,5,8,10H,2H2,(H,11,12) | 0 | 0 | 1 | 0 | 0 | 0 | 0 |
| 581 | InChI=1S/C6H8O6/c7-2-1-3(8)4(9)5(10)6(11)12/h2,4-5,9-10H,1H2,(H,11,12) | 0 | 0 | 1 | 0 | 0 | 0 | 0 |
| 582 | InChI=1S/C6H8O6/c7-2-4(9)6(12)3(8)1-5(10)11/h2,4,6,9,12H,1H2,(H,10,11) | 0 | 0 | 1 | 0 | 0 | 0 | 0 |
| 583 | InChI=1S/C6H8O6/c7-1-3(2-8)4(5(9)10)6(11)12/h1,3-4,8H,2H2,(H,9,10)(H,11,12) | 0 | 0 | 1 | 0 | 0 | 0 | 0 |
| 584 | InChI=1S/C6H8O6/c7-2-3(8)1-4(5(9)10)6(11)12/h2-4,8H,1H2,(H,9,10)(H,11,12) | 0 | 0 | 1 | 0 | 0 | 0 | 0 |
| 585 | InChI=1S/C6H8O6/c7-2-1-3(8)4(5(9)10)6(11)12/h2-4,8H,1H2,(H,9,10)(H,11,12) | 0 | 0 | 1 | 0 | 0 | 0 | 0 |
| 586 | InChI=1S/C6H8O6/c7-1-3(5(9)10)4(2-8)6(11)12/h1,3-4,8H,2H2,(H,9,10)(H,11,12) | 0 | 0 | 1 | 0 | 0 | 0 | 0 |
| 587 | InChI=1S/C6H8O6/c7-2-3(5(9)10)1-4(8)6(11)12/h2-4,8H,1H2,(H,9,10)(H,11,12) | 0 | 0 | 1 | 0 | 0 | 0 | 0 |
| 588 | InChI=1S/C6H8O6/c7-2-1-3(5(9)10)4(8)6(11)12/h2-4,8H,1H2,(H,9,10)(H,11,12) | 0 | 0 | 1 | 0 | 1 | 0 | 0 |
| 589 | InChI=1S/C6H8O6/c7-2-3(6(11)12)4(8)1-5(9)10/h2-4,8H,1H2,(H,9,10)(H,11,12) | 0 | 0 | 1 | 0 | 0 | 0 | 0 |
| 590 | InChI=1S/C6H8O6/c7-2-4(8)3(6(11)12)1-5(9)10/h2-4,8H,1H2,(H,9,10)(H,11,12) | 0 | 0 | 1 | 0 | 1 | 0 | 0 |
| 591 | InChI=1S/C6H8O6/c7-2-3(1-4(8)9)5(10)6(11)12/h2-3,5,10H,1H2,(H,8,9)(H,11,12) | 0 | 0 | 1 | 0 | 0 | 0 | 0 |
| 592 | InChI=1S/C6H8O6/c1-2(7)3(8)4(9)5(10)6(11)12/h2-3,7-8H,1H3,(H,11,12) | 0 | 0 | 1 | 0 | 0 | 0 | 0 |
| 593 | InChI=1S/C6H8O6/c1-2(7)3(8)4(9)5(10)6(11)12/h2,5,7,10H,1H3,(H,11,12) | 0 | 0 | 1 | 0 | 0 | 0 | 0 |
| 594 | InChI=1S/C6H8O6/c1-2(7)3(8)4(9)5(10)6(11)12/h4-5,9-10H,1H3,(H,11,12) | 0 | 0 | 1 | 0 | 0 | 0 | 0 |
| 595 | InChI=1S/C6H8O6/c1-2(7)3(8)4(9)5(10)6(11)12/h2,4,7,9H,1H3,(H,11,12) | 0 | 0 | 1 | 0 | 0 | 0 | 0 |
| 596 | InChI=1S/C6H8O6/c1-2(7)3(5(9)10)4(8)6(11)12/h2-3,7H,1H3,(H,9,10)(H,11,12) | 0 | 0 | 1 | 0 | 0 | 0 | 0 |
| 597 | InChI=1S/C6H8O6/c1-2(7)3(8)4(9)5(10)6(11)12/h3-4,8-9H,1H3,(H,11,12) | 0 | 0 | 1 | 0 | 0 | 0 | 0 |
| 598 | InChI=1S/C6H8O6/c1-2(3(7)5(9)10)4(8)6(11)12/h2-3,7H,1H3,(H,9,10)(H,11,12) | 0 | 0 | 1 | 0 | 1 | 0 | 0 |
| 599 | InChI=1S/C6H8O6/c1-2(5(9)10)3(7)4(8)6(11)12/h2-3,7H,1H3,(H,9,10)(H,11,12) | 0 | 0 | 1 | 0 | 0 | 0 | 0 |
| 600 | InChI=1S/C6H8O6/c1-2(7)3(8)4(9)5(10)6(11)12/h3,5,8,10H,1H3,(H,11,12) | 0 | 0 | 1 | 0 | 0 | 0 | 0 |
| 601 | InChI=1S/C6H8O6/c1-2(7)4(8)3(5(9)10)6(11)12/h2-3,7H,1H3,(H,9,10)(H,11,12) | 0 | 0 | 1 | 0 | 0 | 0 | 0 |
| 602 | InChI=1S/C6H8O6/c1-2(5(9)10)3(7)4(8)6(11)12/h2,4,8H,1H3,(H,9,10)(H,11,12) | 0 | 0 | 1 | 0 | 0 | 0 | 0 |
| 603 | InChI=1S/C6H8O6/c1-2(7)3(5(9)10)4(8)6(11)12/h3-4,8H,1H3,(H,9,10)(H,11,12) | 0 | 0 | 1 | 0 | 1 | 0 | 0 |
| 604 | InChI=1S/C6H8O6/c1-2(7)4(8)3(5(9)10)6(11)12/h3-4,8H,1H3,(H,9,10)(H,11,12) | 0 | 0 | 1 | 0 | 0 | 0 | 0 |
| 605 | InChI=1S/C6H8O6/c1-2(4(7)8)3(5(9)10)6(11)12/h2-3H,1H3,(H,7,8)(H,9,10)(H,11,12) | 1 | 1 | 1 | 0 | 1 | 1 | 0 |
| 606 | InChI=1S/C6H8O6/c1-3(8)6(12,2-7)4(9)5(10)11/h2-3,8,12H,1H3,(H,10,11) | 0 | 0 | 1 | 0 | 0 | 0 | 0 |
| 607 | InChI=1S/C6H8O6/c1-6(12,3(8)2-7)4(9)5(10)11/h2-3,8,12H,1H3,(H,10,11) | 0 | 0 | 1 | 0 | 0 | 0 | 0 |
| 608 | InChI=1S/C6H8O6/c1-6(12,2-7)4(9)3(8)5(10)11/h2,4,9,12H,1H3,(H,10,11) | 0 | 0 | 1 | 0 | 0 | 0 | 0 |
| 609 | InChI=1S/C6H8O6/c1-3(8)6(12,5(10)11)4(9)2-7/h2-3,8,12H,1H3,(H,10,11) | 0 | 0 | 1 | 0 | 0 | 0 | 0 |
| 610 | InChI=1S/C6H8O6/c1-6(12,3(8)2-7)4(9)5(10)11/h2,4,9,12H,1H3,(H,10,11) | 0 | 0 | 1 | 0 | 0 | 0 | 0 |
| 611 | InChI=1S/C6H8O6/c1-3(8)4(9)6(12,2-7)5(10)11/h2-3,8,12H,1H3,(H,10,11) | 0 | 0 | 1 | 0 | 0 | 0 | 0 |
| 612 | InChI=1S/C6H8O6/c1-6(12,5(10)11)4(9)3(8)2-7/h2-3,8,12H,1H3,(H,10,11) | 0 | 0 | 1 | 0 | 0 | 0 | 0 |
| 613 | InChI=1S/C6H8O6/c1-6(12,2-7)4(9)3(8)5(10)11/h2-3,8,12H,1H3,(H,10,11) | 0 | 0 | 1 | 0 | 0 | 0 | 0 |
| 614 | InChI=1S/C6H8O6/c1-3(8)6(12,5(10)11)4(9)2-7/h2,4,9,12H,1H3,(H,10,11) | 0 | 0 | 1 | 0 | 0 | 0 | 0 |
| 615 | InChI=1S/C6H8O6/c1-3(8)6(12,2-7)4(9)5(10)11/h2,4,9,12H,1H3,(H,10,11) | 0 | 0 | 1 | 0 | 0 | 0 | 0 |
| 616 | InChI=1S/C6H8O6/c1-3(8)6(2-7,4(9)10)5(11)12/h2-3,8H,1H3,(H,9,10)(H,11,12) | 0 | 0 | 1 | 0 | 0 | 0 | 0 |
| 617 | InChI=1S/C6H8O6/c1-6(4(9)10,5(11)12)3(8)2-7/h2-3,8H,1H3,(H,9,10)(H,11,12) | 0 | 0 | 1 | 0 | 0 | 0 | 0 |
| 618 | InChI=1S/C6H8O6/c1-3(2-7)6(12,4(8)9)5(10)11/h2-3,12H,1H3,(H,8,9)(H,10,11) | 0 | 0 | 1 | 0 | 0 | 0 | 0 |
| 619 | InChI=1S/C6H8O6/c1-6(12,5(10)11)4(9)3(8)2-7/h2,4,9,12H,1H3,(H,10,11) | 0 | 0 | 1 | 0 | 0 | 0 | 0 |
| 620 | InChI=1S/C6H8O6/c1-3(8)4(9)6(12,2-7)5(10)11/h2,4,9,12H,1H3,(H,10,11) | 0 | 0 | 1 | 0 | 0 | 0 | 0 |
| 621 | InChI=1S/C6H8O6/c1-6(2-7,5(11)12)3(8)4(9)10/h2-3,8H,1H3,(H,9,10)(H,11,12) | 0 | 0 | 1 | 0 | 0 | 0 | 0 |
| 622 | InChI=1S/C6H8O6/c1-3(4(8)9)6(12,2-7)5(10)11/h2-3,12H,1H3,(H,8,9)(H,10,11) | 0 | 0 | 1 | 0 | 0 | 0 | 0 |
| 623 | InChI=1S/C6H8O6/c1-6(12,5(10)11)3(2-7)4(8)9/h2-3,12H,1H3,(H,8,9)(H,10,11) | 0 | 0 | 1 | 0 | 0 | 0 | 0 |
| 624 | InChI=1S/C6H8O6/c1-6(12,2-7)3(4(8)9)5(10)11/h2-3,12H,1H3,(H,8,9)(H,10,11) | 0 | 0 | 1 | 0 | 0 | 0 | 0 |
| 625 | InChI=1S/C6H8O6/c7-1-3(9)5(11)6(12)4(10)2-8/h3,7-9H,1-2H2 | 0 | 0 | 1 | 0 | 1 | 0 | 0 |
| 626 | InChI=1S/C6H8O6/c7-1-3(2-8)4(9)5(10)6(11)12/h3,7-8H,1-2H2,(H,11,12) | 0 | 0 | 1 | 0 | 0 | 0 | 0 |
| 627 | InChI=1S/C6H8O6/c7-2-1-3(8)4(9)5(10)6(11)12/h3,7-8H,1-2H2,(H,11,12) | 0 | 0 | 1 | 0 | 0 | 0 | 0 |
| 628 | InChI=1S/C6H8O6/c7-2-3(8)1-4(9)5(10)6(11)12/h3,7-8H,1-2H2,(H,11,12) | 0 | 0 | 1 | 0 | 0 | 0 | 0 |
| 629 | InChI=1S/C6H8O6/c7-1-3(9)5(11)6(12)4(10)2-8/h5,7-8,11H,1-2H2 | 0 | 0 | 1 | 0 | 0 | 0 | 0 |
| 630 | InChI=1S/C6H8O6/c7-1-3(6(11)12)5(10)4(9)2-8/h3,7-8H,1-2H2,(H,11,12) | 0 | 0 | 1 | 0 | 0 | 0 | 0 |
| 631 | InChI=1S/C6H8O6/c7-2-1-3(8)4(9)5(10)6(11)12/h5,7,10H,1-2H2,(H,11,12) | 0 | 0 | 1 | 0 | 0 | 0 | 0 |
| 632 | InChI=1S/C6H8O6/c7-2-4(9)6(12)3(8)1-5(10)11/h4,7,9H,1-2H2,(H,10,11) | 0 | 0 | 1 | 0 | 0 | 0 | 0 |
| 633 | InChI=1S/C6H8O6/c7-2-4(9)6(12)3(8)1-5(10)11/h3,7-8H,1-2H2,(H,10,11) | 0 | 0 | 1 | 0 | 0 | 0 | 0 |
| 634 | InChI=1S/C6H8O6/c7-1-3(4(9)2-8)5(10)6(11)12/h3,7-8H,1-2H2,(H,11,12) | 0 | 0 | 1 | 0 | 0 | 0 | 0 |
| 635 | InChI=1S/C6H8O6/c7-2-1-3(8)4(9)5(10)6(11)12/h4,7,9H,1-2H2,(H,11,12) | 0 | 0 | 1 | 0 | 0 | 0 | 0 |
| 636 | InChI=1S/C6H8O6/c7-2-1-3(5(9)10)4(8)6(11)12/h3,7H,1-2H2,(H,9,10)(H,11,12) | 0 | 0 | 1 | 0 | 0 | 0 | 0 |
| 637 | InChI=1S/C6H8O6/c7-2-3(8)1-4(9)5(10)6(11)12/h4,7,9H,1-2H2,(H,11,12) | 0 | 0 | 1 | 0 | 0 | 0 | 0 |
| 638 | InChI=1S/C6H8O6/c7-2-3(1-4(8)9)5(10)6(11)12/h3,7H,1-2H2,(H,8,9)(H,11,12) | 0 | 0 | 1 | 0 | 1 | 0 | 0 |
| 639 | InChI=1S/C6H8O6/c7-3(1-2-4(8)9)5(10)6(11)12/h3,7H,1-2H2,(H,8,9)(H,11,12) | 0 | 0 | 1 | 0 | 1 | 0 | 0 |
| 640 | InChI=1S/C6H8O6/c7-2-5(10)3(8)1-4(9)6(11)12/h4,7,9H,1-2H2,(H,11,12) | 0 | 0 | 1 | 0 | 0 | 0 | 0 |
| 641 | InChI=1S/C6H8O6/c7-2-5(10)3(8)1-4(9)6(11)12/h5,7,10H,1-2H2,(H,11,12) | 0 | 0 | 1 | 0 | 1 | 0 | 0 |
| 642 | InChI=1S/C6H8O6/c7-2-5(10)3(8)1-4(9)6(11)12/h3,7-8H,1-2H2,(H,11,12) | 0 | 0 | 1 | 0 | 1 | 1 | 0 |
| 643 | InChI=1S/C6H8O6/c7-2-3(5(9)10)1-4(8)6(11)12/h3,7H,1-2H2,(H,9,10)(H,11,12) | 0 | 0 | 1 | 0 | 1 | 1 | 0 |
| 644 | InChI=1S/C6H8O6/c7-3(5(9)10)1-2-4(8)6(11)12/h3,7H,1-2H2,(H,9,10)(H,11,12) | 1 | 0 | 1 | 0 | 1 | 1 | 0 |
| 645 | InChI=1S/C6H8O6/c7-3(2-5(9)10)1-4(8)6(11)12/h3,7H,1-2H2,(H,9,10)(H,11,12) | 0 | 0 | 1 | 0 | 1 | 0 | 0 |
| 646 | InChI=1S/C6H8O6/c7-1-3(9)5(6(11)12)4(10)2-8/h5,7-8H,1-2H2,(H,11,12) | 0 | 0 | 1 | 0 | 0 | 0 | 0 |
| 647 | InChI=1S/C6H8O6/c7-2-4(9)6(12)3(8)1-5(10)11/h6-7,12H,1-2H2,(H,10,11) | 0 | 0 | 1 | 0 | 0 | 0 | 0 |
| 648 | InChI=1S/C6H8O6/c7-2-1-3(8)4(5(9)10)6(11)12/h4,7H,1-2H2,(H,9,10)(H,11,12) | 0 | 0 | 1 | 0 | 0 | 0 | 0 |
| 649 | InChI=1S/C6H8O6/c7-2-3(8)1-4(9)5(10)6(11)12/h5,7,10H,1-2H2,(H,11,12) | 0 | 0 | 1 | 0 | 0 | 0 | 0 |
| 650 | InChI=1S/C6H8O6/c7-2-3(6(11)12)4(8)1-5(9)10/h3,7H,1-2H2,(H,9,10)(H,11,12) | 0 | 0 | 1 | 0 | 1 | 0 | 0 |
| 651 | InChI=1S/C6H8O6/c7-2-4(8)3(6(11)12)1-5(9)10/h3,7H,1-2H2,(H,9,10)(H,11,12) | 0 | 0 | 1 | 0 | 1 | 0 | 0 |
| 652 | InChI=1S/C6H8O6/c7-3(1-2-4(8)9)5(10)6(11)12/h5,10H,1-2H2,(H,8,9)(H,11,12) | 0 | 1 | 1 | 0 | 1 | 1 | 0 |
| 653 | InChI=1S/C6H8O6/c7-3(1-5(9)10)4(8)2-6(11)12/h3,7H,1-2H2,(H,9,10)(H,11,12) | 0 | 0 | 1 | 0 | 0 | 0 | 0 |
| 654 | InChI=1S/C6H8O6/c7-2-3(8)1-4(5(9)10)6(11)12/h4,7H,1-2H2,(H,9,10)(H,11,12) | 0 | 0 | 1 | 0 | 0 | 0 | 0 |
| 655 | InChI=1S/C6H8O6/c7-4(8)2-1-3(5(9)10)6(11)12/h3H,1-2H2,(H,7,8)(H,9,10)(H,11,12) | 1 | 0 | 1 | 0 | 1 | 1 | 0 |
| 656 | InChI=1S/C6H8O6/c7-3(2-5(9)10)1-4(8)6(11)12/h4,8H,1-2H2,(H,9,10)(H,11,12) | 0 | 0 | 1 | 0 | 1 | 0 | 0 |
| 657 | InChI=1S/C6H8O6/c7-4(8)1-3(6(11)12)2-5(9)10/h3H,1-2H2,(H,7,8)(H,9,10)(H,11,12) | 1 | 1 | 1 | 1 | 1 | 1 | 0 |
| 658 | InChI=1S/C6H8O6/c7-1-4(10)5(11)6(12,2-8)3-9/h1,8-9,12H,2-3H2 | 0 | 0 | 1 | 0 | 0 | 0 | 0 |
| 659 | InChI=1S/C6H8O6/c7-1-4(10)5(11)6(12,2-8)3-9/h2,7,9,12H,1,3H2 | 0 | 0 | 1 | 0 | 0 | 0 | 0 |
| 660 | InChI=1S/C6H8O6/c7-1-6(2-8,3-9)4(10)5(11)12/h1,8-9H,2-3H2,(H,11,12) | 0 | 0 | 1 | 0 | 0 | 0 | 0 |
| 661 | InChI=1S/C6H8O6/c7-2-1-6(12,3-8)4(9)5(10)11/h3,7,12H,1-2H2,(H,10,11) | 0 | 0 | 1 | 0 | 0 | 0 | 0 |
| 662 | InChI=1S/C6H8O6/c7-2-1-6(12,3-8)4(9)5(10)11/h2,8,12H,1,3H2,(H,10,11) | 0 | 0 | 1 | 0 | 0 | 0 | 0 |
| 663 | InChI=1S/C6H8O6/c7-2-6(12,3-8)1-4(9)5(10)11/h2,8,12H,1,3H2,(H,10,11) | 0 | 0 | 1 | 0 | 0 | 0 | 0 |
| 664 | InChI=1S/C6H8O6/c7-1-4(10)6(12,3-9)5(11)2-8/h1,8-9,12H,2-3H2 | 0 | 0 | 1 | 0 | 0 | 0 | 0 |
| 665 | InChI=1S/C6H8O6/c7-1-4(10)6(2-8,3-9)5(11)12/h1,8-9H,2-3H2,(H,11,12) | 0 | 0 | 1 | 0 | 0 | 0 | 0 |
| 666 | InChI=1S/C6H8O6/c7-2-1-6(12,5(10)11)4(9)3-8/h3,7,12H,1-2H2,(H,10,11) | 0 | 0 | 1 | 0 | 0 | 0 | 0 |
| 667 | InChI=1S/C6H8O6/c7-2-4(9)6(12,3-8)1-5(10)11/h2,8,12H,1,3H2,(H,10,11) | 0 | 0 | 1 | 0 | 0 | 0 | 0 |
| 668 | InChI=1S/C6H8O6/c7-1-4(10)6(12,3-9)5(11)2-8/h3,7-8,12H,1-2H2 | 0 | 0 | 1 | 0 | 0 | 0 | 0 |
| 669 | InChI=1S/C6H8O6/c7-1-4(10)6(2-8,3-9)5(11)12/h2,7,9H,1,3H2,(H,11,12) | 0 | 0 | 1 | 0 | 0 | 0 | 0 |
| 670 | InChI=1S/C6H8O6/c7-2-1-4(9)6(12,3-8)5(10)11/h3,7,12H,1-2H2,(H,10,11) | 0 | 0 | 1 | 0 | 0 | 0 | 0 |
| 671 | InChI=1S/C6H8O6/c7-2-1-4(9)6(12,3-8)5(10)11/h2,8,12H,1,3H2,(H,10,11) | 0 | 0 | 1 | 0 | 0 | 0 | 0 |
| 672 | InChI=1S/C6H8O6/c7-2-1-6(12,5(10)11)4(9)3-8/h2,8,12H,1,3H2,(H,10,11) | 0 | 0 | 1 | 0 | 0 | 0 | 0 |
| 673 | InChI=1S/C6H8O6/c7-2-6(12,3-8)4(9)1-5(10)11/h2,8,12H,1,3H2,(H,10,11) | 0 | 0 | 1 | 0 | 0 | 0 | 0 |
| 674 | InChI=1S/C6H8O6/c7-2-4(9)6(12,3-8)1-5(10)11/h3,7,12H,1-2H2,(H,10,11) | 0 | 0 | 1 | 0 | 0 | 0 | 0 |
| 675 | InChI=1S/C6H8O6/c7-2-1-6(3-8,4(9)10)5(11)12/h3,7H,1-2H2,(H,9,10)(H,11,12) | 0 | 0 | 1 | 0 | 1 | 0 | 0 |
| 676 | InChI=1S/C6H8O6/c7-2-1-6(3-8,4(9)10)5(11)12/h2,8H,1,3H2,(H,9,10)(H,11,12) | 0 | 0 | 1 | 0 | 0 | 0 | 0 |
| 677 | InChI=1S/C6H8O6/c7-3-1-2-6(12,4(8)9)5(10)11/h3,12H,1-2H2,(H,8,9)(H,10,11) | 0 | 0 | 1 | 0 | 0 | 0 | 0 |
| 678 | InChI=1S/C6H8O6/c7-2-4(9)1-6(12,3-8)5(10)11/h2,8,12H,1,3H2,(H,10,11) | 0 | 0 | 1 | 0 | 0 | 0 | 0 |
| 679 | InChI=1S/C6H8O6/c7-2-4(9)1-6(12,3-8)5(10)11/h3,7,12H,1-2H2,(H,10,11) | 0 | 0 | 1 | 0 | 0 | 0 | 0 |
| 680 | InChI=1S/C6H8O6/c7-2-6(3-8,5(11)12)1-4(9)10/h2,8H,1,3H2,(H,9,10)(H,11,12) | 0 | 0 | 1 | 0 | 0 | 0 | 0 |
| 681 | InChI=1S/C6H8O6/c7-3-6(12,5(10)11)2-1-4(8)9/h3,12H,1-2H2,(H,8,9)(H,10,11) | 0 | 0 | 1 | 0 | 0 | 0 | 0 |
| 682 | InChI=1S/C6H8O6/c7-2-1-6(12,5(10)11)3-4(8)9/h2,12H,1,3H2,(H,8,9)(H,10,11) | 0 | 0 | 1 | 0 | 1 | 0 | 0 |
| 683 | InChI=1S/C6H8O6/c7-3-6(12,1-4(8)9)2-5(10)11/h3,12H,1-2H2,(H,8,9)(H,10,11) | 0 | 0 | 1 | 0 | 1 | 0 | 0 |
| 684 | InChI=1S/C6H8O6/c1-6(12,2-7)4(9)3(8)5(10)11/h7,12H,2H2,1H3,(H,10,11) | 0 | 0 | 1 | 0 | 0 | 0 | 0 |
| 685 | InChI=1S/C6H8O6/c1-6(12,5(10)11)4(9)3(8)2-7/h7,12H,2H2,1H3,(H,10,11) | 0 | 0 | 1 | 0 | 0 | 0 | 0 |
| 686 | InChI=1S/C6H8O6/c1-3(8)4(9)6(12,2-7)5(10)11/h7,12H,2H2,1H3,(H,10,11) | 0 | 0 | 1 | 0 | 0 | 0 | 0 |
| 687 | InChI=1S/C6H8O6/c1-6(12,3(8)2-7)4(9)5(10)11/h7,12H,2H2,1H3,(H,10,11) | 0 | 0 | 1 | 0 | 0 | 0 | 0 |
| 688 | InChI=1S/C6H8O6/c1-3(8)6(12,2-7)4(9)5(10)11/h7,12H,2H2,1H3,(H,10,11) | 0 | 0 | 1 | 0 | 0 | 0 | 0 |
| 689 | InChI=1S/C6H8O6/c1-6(2-7,5(11)12)3(8)4(9)10/h7H,2H2,1H3,(H,9,10)(H,11,12) | 0 | 0 | 1 | 0 | 0 | 0 | 0 |
| 690 | InChI=1S/C6H8O6/c1-2-6(12,5(10)11)3(7)4(8)9/h12H,2H2,1H3,(H,8,9)(H,10,11) | 0 | 0 | 1 | 0 | 0 | 0 | 0 |
| 691 | InChI=1S/C6H8O6/c1-6(12,2-3(7)8)4(9)5(10)11/h12H,2H2,1H3,(H,7,8)(H,10,11) | 0 | 0 | 1 | 0 | 1 | 0 | 0 |
| 692 | InChI=1S/C6H8O6/c1-6(12,5(10)11)2-3(7)4(8)9/h12H,2H2,1H3,(H,8,9)(H,10,11) | 1 | 1 | 1 | 0 | 1 | 1 | 0 |
| 693 | InChI=1S/C6H8O6/c1-3(8)6(12,5(10)11)4(9)2-7/h7,12H,2H2,1H3,(H,10,11) | 0 | 0 | 1 | 0 | 0 | 0 | 0 |
| 694 | InChI=1S/C6H8O6/c1-6(4(9)10,5(11)12)3(8)2-7/h7H,2H2,1H3,(H,9,10)(H,11,12) | 0 | 0 | 1 | 0 | 0 | 0 | 0 |
| 695 | InChI=1S/C6H8O6/c1-2-3(7)6(12,4(8)9)5(10)11/h12H,2H2,1H3,(H,8,9)(H,10,11) | 0 | 0 | 1 | 0 | 0 | 0 | 0 |
| 696 | InChI=1S/C6H8O6/c1-6(12,5(10)11)3(7)2-4(8)9/h12H,2H2,1H3,(H,8,9)(H,10,11) | 0 | 0 | 1 | 0 | 0 | 0 | 0 |
| 697 | InChI=1S/C6H8O6/c1-3(8)6(2-7,4(9)10)5(11)12/h7H,2H2,1H3,(H,9,10)(H,11,12) | 0 | 0 | 1 | 0 | 0 | 0 | 0 |
| 698 | InChI=1S/C6H8O6/c1-3(7)6(12,5(10)11)2-4(8)9/h12H,2H2,1H3,(H,8,9)(H,10,11) | 0 | 0 | 1 | 0 | 1 | 0 | 0 |
| 699 | InChI=1S/C6H8O6/c1-2-6(3(7)8,4(9)10)5(11)12/h2H2,1H3,(H,7,8)(H,9,10)(H,11,12) | 0 | 1 | 1 | 0 | 1 | 1 | 0 |
| 700 | InChI=1S/C6H8O6/c1-3(7)2-6(12,4(8)9)5(10)11/h12H,2H2,1H3,(H,8,9)(H,10,11) | 0 | 0 | 1 | 0 | 0 | 0 | 0 |
| 701 | InChI=1S/C6H8O6/c1-6(4(9)10,5(11)12)2-3(7)8/h2H2,1H3,(H,7,8)(H,9,10)(H,11,12) | 1 | 0 | 1 | 0 | 1 | 1 | 0 |
| 702 | InChI=1S/C6H8O6/c7-1-5(11,2-8)6(12,3-9)4-10/h1-3,10-12H,4H2 | 0 | 0 | 1 | 0 | 0 | 0 | 0 |
| 703 | InChI=1S/C6H8O6/c1-5(11,2-7)6(12,3-8)4(9)10/h2-3,11-12H,1H3,(H,9,10) | 0 | 0 | 1 | 0 | 0 | 0 | 0 |
| 704 | InChI=1S/C6H8O6/c1-5(11,4(9)10)6(12,2-7)3-8/h2-3,11-12H,1H3,(H,9,10) | 0 | 0 | 1 | 0 | 0 | 0 | 0 |
| 705 | InChI=1S/C6H8O7/c7-1-3(9)6(13,2-8)4(10)5(11)12/h2-3,7,9,13H,1H2,(H,11,12) | 0 | 0 | 1 | 0 | 0 | 0 | 0 |
| 706 | InChI=1S/C6H8O7/c7-1-3(9)6(13,2-8)4(10)5(11)12/h1,3,8-9,13H,2H2,(H,11,12) | 0 | 0 | 1 | 0 | 0 | 0 | 0 |
| 707 | InChI=1S/C6H8O7/c7-1-6(13,2-8)4(10)3(9)5(11)12/h1,4,8,10,13H,2H2,(H,11,12) | 0 | 0 | 1 | 0 | 0 | 0 | 0 |
| 708 | InChI=1S/C6H8O7/c7-1-3(9)6(13,5(11)12)4(10)2-8/h1,4,8,10,13H,2H2,(H,11,12) | 0 | 0 | 1 | 0 | 0 | 0 | 0 |
| 709 | InChI=1S/C6H8O7/c7-1-3(9)6(13,2-8)4(10)5(11)12/h1,4,8,10,13H,2H2,(H,11,12) | 0 | 0 | 1 | 0 | 0 | 0 | 0 |
| 710 | InChI=1S/C6H8O7/c7-1-3(9)4(10)6(13,2-8)5(11)12/h2-3,7,9,13H,1H2,(H,11,12) | 0 | 0 | 1 | 0 | 0 | 0 | 0 |
| 711 | InChI=1S/C6H8O7/c7-1-3(9)4(10)6(13,2-8)5(11)12/h1,3,8-9,13H,2H2,(H,11,12) | 0 | 0 | 1 | 0 | 0 | 0 | 0 |
| 712 | InChI=1S/C6H8O7/c7-1-6(13,2-8)4(10)3(9)5(11)12/h1,3,8-9,13H,2H2,(H,11,12) | 0 | 0 | 1 | 0 | 0 | 0 | 0 |
| 713 | InChI=1S/C6H8O7/c7-1-3(9)6(13,5(11)12)4(10)2-8/h1,3,8-9,13H,2H2,(H,11,12) | 0 | 0 | 1 | 0 | 0 | 0 | 0 |
| 714 | InChI=1S/C6H8O7/c7-1-3(9)6(13,2-8)4(10)5(11)12/h2,4,7,10,13H,1H2,(H,11,12) | 0 | 0 | 1 | 0 | 0 | 0 | 0 |
| 715 | InChI=1S/C6H8O7/c7-1-3(9)6(2-8,4(10)11)5(12)13/h2-3,7,9H,1H2,(H,10,11)(H,12,13) | 0 | 0 | 1 | 0 | 0 | 0 | 0 |
| 716 | InChI=1S/C6H8O7/c7-1-3(9)6(2-8,4(10)11)5(12)13/h1,3,8-9H,2H2,(H,10,11)(H,12,13) | 0 | 0 | 1 | 0 | 0 | 0 | 0 |
| 717 | InChI=1S/C6H8O7/c7-1-3(2-8)6(13,4(9)10)5(11)12/h1,3,8,13H,2H2,(H,9,10)(H,11,12) | 0 | 0 | 1 | 0 | 0 | 0 | 0 |
| 718 | InChI=1S/C6H8O7/c7-2-1-3(8)6(13,4(9)10)5(11)12/h2-3,8,13H,1H2,(H,9,10)(H,11,12) | 0 | 0 | 1 | 0 | 0 | 0 | 0 |
| 719 | InChI=1S/C6H8O7/c7-2-3(8)1-6(13,4(9)10)5(11)12/h2-3,8,13H,1H2,(H,9,10)(H,11,12) | 0 | 0 | 1 | 0 | 0 | 0 | 0 |
| 720 | InChI=1S/C6H8O7/c7-1-3(9)4(10)6(13,2-8)5(11)12/h1,4,8,10,13H,2H2,(H,11,12) | 0 | 0 | 1 | 0 | 0 | 0 | 0 |
| 721 | InChI=1S/C6H8O7/c7-1-3(9)4(10)6(13,2-8)5(11)12/h2,4,7,10,13H,1H2,(H,11,12) | 0 | 0 | 1 | 0 | 0 | 0 | 0 |
| 722 | InChI=1S/C6H8O7/c7-1-6(2-8,5(12)13)3(9)4(10)11/h1,3,8-9H,2H2,(H,10,11)(H,12,13) | 0 | 0 | 1 | 0 | 0 | 0 | 0 |
| 723 | InChI=1S/C6H8O7/c7-1-3(4(9)10)6(13,2-8)5(11)12/h2-3,7,13H,1H2,(H,9,10)(H,11,12) | 0 | 0 | 1 | 0 | 0 | 0 | 0 |
| 724 | InChI=1S/C6H8O7/c7-2-6(13,5(11)12)1-3(8)4(9)10/h2-3,8,13H,1H2,(H,9,10)(H,11,12) | 0 | 0 | 1 | 0 | 0 | 0 | 0 |
| 725 | InChI=1S/C6H8O7/c7-1-3(4(9)10)6(13,2-8)5(11)12/h1,3,8,13H,2H2,(H,9,10)(H,11,12) | 0 | 0 | 1 | 0 | 0 | 0 | 0 |
| 726 | InChI=1S/C6H8O7/c7-2-1-6(13,5(11)12)3(8)4(9)10/h2-3,8,13H,1H2,(H,9,10)(H,11,12) | 0 | 0 | 1 | 0 | 0 | 0 | 0 |
| 727 | InChI=1S/C6H8O7/c7-2-6(13,5(11)12)3(8)1-4(9)10/h2-3,8,13H,1H2,(H,9,10)(H,11,12) | 0 | 0 | 1 | 0 | 0 | 0 | 0 |
| 728 | InChI=1S/C6H8O7/c7-2-3(8)6(13,5(11)12)1-4(9)10/h2-3,8,13H,1H2,(H,9,10)(H,11,12) | 0 | 0 | 1 | 0 | 0 | 0 | 0 |
| 729 | InChI=1S/C6H8O7/c7-1-6(13,2-8)3(4(9)10)5(11)12/h1,3,8,13H,2H2,(H,9,10)(H,11,12) | 0 | 0 | 1 | 0 | 0 | 0 | 0 |
| 730 | InChI=1S/C6H8O7/c7-2-6(13,1-3(8)9)4(10)5(11)12/h2,4,10,13H,1H2,(H,8,9)(H,11,12) | 0 | 0 | 1 | 0 | 0 | 0 | 0 |
| 731 | InChI=1S/C6H8O7/c1-2(7)6(13,5(11)12)3(8)4(9)10/h2,7,13H,1H3,(H,9,10)(H,11,12) | 0 | 0 | 1 | 0 | 0 | 0 | 0 |
| 732 | InChI=1S/C6H8O7/c1-6(13,2(7)4(9)10)3(8)5(11)12/h2,7,13H,1H3,(H,9,10)(H,11,12) | 0 | 0 | 1 | 0 | 0 | 0 | 0 |
| 733 | InChI=1S/C6H8O7/c1-6(13,5(11)12)3(8)2(7)4(9)10/h3,8,13H,1H3,(H,9,10)(H,11,12) | 0 | 0 | 1 | 0 | 0 | 0 | 0 |
| 734 | InChI=1S/C6H8O7/c1-2(7)3(8)6(13,4(9)10)5(11)12/h2,7,13H,1H3,(H,9,10)(H,11,12) | 0 | 0 | 1 | 0 | 0 | 0 | 0 |
| 735 | InChI=1S/C6H8O7/c1-6(13,5(11)12)3(8)2(7)4(9)10/h2,7,13H,1H3,(H,9,10)(H,11,12) | 0 | 0 | 1 | 0 | 0 | 0 | 0 |
| 736 | InChI=1S/C6H8O7/c1-2(7)6(13,5(11)12)3(8)4(9)10/h3,8,13H,1H3,(H,9,10)(H,11,12) | 0 | 0 | 1 | 0 | 1 | 0 | 0 |
| 737 | InChI=1S/C6H8O7/c1-2(7)6(3(8)9,4(10)11)5(12)13/h2,7H,1H3,(H,8,9)(H,10,11)(H,12,13) | 0 | 0 | 1 | 0 | 1 | 0 | 0 |
| 738 | InChI=1S/C6H8O7/c1-2(7)3(8)6(13,4(9)10)5(11)12/h3,8,13H,1H3,(H,9,10)(H,11,12) | 0 | 0 | 1 | 0 | 0 | 0 | 0 |
| 739 | InChI=1S/C6H8O7/c1-6(4(10)11,5(12)13)2(7)3(8)9/h2,7H,1H3,(H,8,9)(H,10,11)(H,12,13) | 0 | 0 | 1 | 0 | 0 | 0 | 0 |
| 740 | InChI=1S/C6H8O7/c1-2(3(7)8)6(13,4(9)10)5(11)12/h2,13H,1H3,(H,7,8)(H,9,10)(H,11,12) | 0 | 0 | 1 | 0 | 0 | 0 | 0 |
| 741 | InChI=1S/C6H8O7/c1-6(13,5(11)12)2(3(7)8)4(9)10/h2,13H,1H3,(H,7,8)(H,9,10)(H,11,12) | 0 | 0 | 1 | 0 | 1 | 0 | 0 |
| 742 | InChI=1S/C6H8O7/c7-1-2(8)3(9)4(10)5(11)6(12)13/h1-4,8-10H,(H,12,13) | 0 | 0 | 1 | 0 | 1 | 0 | 0 |
| 743 | InChI=1S/C6H8O7/c7-1-2(8)3(9)4(10)5(11)6(12)13/h1,3-5,9-11H,(H,12,13) | 0 | 0 | 1 | 0 | 1 | 0 | 0 |
| 744 | InChI=1S/C6H8O7/c7-1-2(8)3(9)4(10)5(11)6(12)13/h1-3,5,8-9,11H,(H,12,13) | 0 | 0 | 1 | 0 | 0 | 0 | 0 |
| 745 | InChI=1S/C6H8O7/c7-1-2(8)3(9)4(10)5(11)6(12)13/h1-2,4-5,8,10-11H,(H,12,13) | 1 | 0 | 1 | 0 | 0 | 1 | 0 |
| 746 | InChI=1S/C6H8O7/c7-1-2(8)4(9)3(5(10)11)6(12)13/h1-4,8-9H,(H,10,11)(H,12,13) | 0 | 0 | 1 | 0 | 0 | 0 | 0 |
| 747 | InChI=1S/C6H8O7/c7-1-2(5(10)11)3(8)4(9)6(12)13/h1-4,8-9H,(H,10,11)(H,12,13) | 0 | 0 | 1 | 0 | 0 | 0 | 0 |
| 748 | InChI=1S/C6H8O7/c7-1-2(8)3(5(10)11)4(9)6(12)13/h1-4,8-9H,(H,10,11)(H,12,13) | 0 | 0 | 1 | 0 | 0 | 0 | 0 |
| 749 | InChI=1S/C6H8O7/c7-1-2(3(8)5(10)11)4(9)6(12)13/h1-4,8-9H,(H,10,11)(H,12,13) | 0 | 0 | 1 | 0 | 0 | 0 | 0 |
| 750 | InChI=1S/C6H8O7/c7-1-2(8)3(9)4(10)5(11)6(12)13/h2-3,7-9H,1H2,(H,12,13) | 1 | 0 | 1 | 0 | 1 | 1 | 0 |
| 751 | InChI=1S/C6H8O7/c7-1-2(8)3(9)4(10)5(11)6(12)13/h2,5,7-8,11H,1H2,(H,12,13) | 0 | 0 | 1 | 0 | 0 | 0 | 0 |
| 752 | InChI=1S/C6H8O7/c7-1-2(8)3(9)4(10)5(11)6(12)13/h4-5,7,10-11H,1H2,(H,12,13) | 0 | 0 | 1 | 0 | 0 | 0 | 0 |
| 753 | InChI=1S/C6H8O7/c7-1-2(8)3(9)4(10)5(11)6(12)13/h2,4,7-8,10H,1H2,(H,12,13) | 0 | 0 | 1 | 0 | 1 | 0 | 0 |
| 754 | InChI=1S/C6H8O7/c7-1-2(8)3(5(10)11)4(9)6(12)13/h2-3,7-8H,1H2,(H,10,11)(H,12,13) | 0 | 0 | 1 | 0 | 0 | 0 | 0 |
| 755 | InChI=1S/C6H8O7/c7-1-2(8)3(9)4(10)5(11)6(12)13/h3-4,7,9-10H,1H2,(H,12,13) | 1 | 0 | 1 | 0 | 1 | 1 | 0 |
| 756 | InChI=1S/C6H8O7/c7-1-2(3(8)5(10)11)4(9)6(12)13/h2-3,7-8H,1H2,(H,10,11)(H,12,13) | 0 | 0 | 1 | 0 | 0 | 0 | 0 |
| 757 | InChI=1S/C6H8O7/c7-1-2(5(10)11)3(8)4(9)6(12)13/h2-3,7-8H,1H2,(H,10,11)(H,12,13) | 0 | 0 | 1 | 0 | 0 | 0 | 0 |
| 758 | InChI=1S/C6H8O7/c7-2(4(9)6(12)13)1-3(8)5(10)11/h2-3,7-8H,1H2,(H,10,11)(H,12,13) | 0 | 0 | 1 | 0 | 0 | 0 | 0 |
| 759 | InChI=1S/C6H8O7/c7-2(1-3(8)9)4(10)5(11)6(12)13/h2,4,7,10H,1H2,(H,8,9)(H,12,13) | 0 | 0 | 1 | 0 | 0 | 0 | 0 |
| 760 | InChI=1S/C6H8O7/c7-2(4(9)6(12)13)1-3(8)5(10)11/h2,4,7,9H,1H2,(H,10,11)(H,12,13) | 1 | 0 | 1 | 0 | 1 | 1 | 0 |
| 761 | InChI=1S/C6H8O7/c7-1-2(8)3(9)4(10)5(11)6(12)13/h3,5,7,9,11H,1H2,(H,12,13) | 0 | 0 | 1 | 0 | 0 | 0 | 0 |
| 762 | InChI=1S/C6H8O7/c7-1-2(8)4(9)3(5(10)11)6(12)13/h2-3,7-8H,1H2,(H,10,11)(H,12,13) | 0 | 0 | 1 | 0 | 0 | 0 | 0 |
| 763 | InChI=1S/C6H8O7/c7-1-2(5(10)11)3(8)4(9)6(12)13/h2,4,7,9H,1H2,(H,10,11)(H,12,13) | 0 | 0 | 1 | 0 | 0 | 0 | 0 |
| 764 | InChI=1S/C6H8O7/c7-2(1-3(8)9)4(10)5(11)6(12)13/h2,5,7,11H,1H2,(H,8,9)(H,12,13) | 0 | 0 | 1 | 0 | 0 | 0 | 0 |
| 765 | InChI=1S/C6H8O7/c7-1-2(8)3(5(10)11)4(9)6(12)13/h3-4,7,9H,1H2,(H,10,11)(H,12,13) | 0 | 0 | 1 | 0 | 0 | 0 | 0 |
| 766 | InChI=1S/C6H8O7/c7-2(4(9)6(12)13)1-3(8)5(10)11/h3-4,8-9H,1H2,(H,10,11)(H,12,13) | 0 | 0 | 1 | 0 | 0 | 0 | 0 |
| 767 | InChI=1S/C6H8O7/c7-1-2(8)4(9)3(5(10)11)6(12)13/h3-4,7,9H,1H2,(H,10,11)(H,12,13) | 0 | 0 | 1 | 0 | 0 | 0 | 0 |
| 768 | InChI=1S/C6H8O7/c7-2(1-3(8)9)4(10)5(11)6(12)13/h4-5,10-11H,1H2,(H,8,9)(H,12,13) | 0 | 0 | 1 | 0 | 0 | 0 | 0 |
| 769 | InChI=1S/C6H8O7/c7-1-2(4(8)9)3(5(10)11)6(12)13/h2-3,7H,1H2,(H,8,9)(H,10,11)(H,12,13) | 0 | 0 | 1 | 0 | 0 | 0 | 0 |
| 770 | InChI=1S/C6H8O7/c7-3(6(12)13)1-2(4(8)9)5(10)11/h2-3,7H,1H2,(H,8,9)(H,10,11)(H,12,13) | 0 | 0 | 1 | 0 | 0 | 1 | 0 |
| 771 | InChI=1S/C6H8O7/c7-2(1-3(8)9)4(5(10)11)6(12)13/h2,4,7H,1H2,(H,8,9)(H,10,11)(H,12,13) | 0 | 0 | 1 | 0 | 1 | 0 | 0 |
| 772 | InChI=1S/C6H8O7/c7-3(8)1-2(5(10)11)4(9)6(12)13/h2,4,9H,1H2,(H,7,8)(H,10,11)(H,12,13) | 1 | 1 | 1 | 1 | 1 | 1 | 1 |
| 773 | InChI=1S/C6H8O7/c7-1-3(9)4(10)6(13,2-8)5(11)12/h1-4,9-10,13H,(H,11,12) | 0 | 0 | 1 | 0 | 0 | 0 | 0 |
| 774 | InChI=1S/C6H8O7/c7-1-3(9)6(13,5(11)12)4(10)2-8/h1-4,9-10,13H,(H,11,12) | 0 | 0 | 1 | 0 | 0 | 0 | 0 |
| 775 | InChI=1S/C6H8O7/c7-1-6(13,2-8)4(10)3(9)5(11)12/h1-4,9-10,13H,(H,11,12) | 0 | 0 | 1 | 0 | 0 | 0 | 0 |
| 776 | InChI=1S/C6H8O7/c7-1-3(9)6(13,2-8)4(10)5(11)12/h1-4,9-10,13H,(H,11,12) | 0 | 0 | 1 | 0 | 0 | 0 | 0 |
| 777 | InChI=1S/C6H8O7/c7-1-6(13,2-8)4(10)3(9)5(11)12/h7-8,13H,1-2H2,(H,11,12) | 0 | 0 | 1 | 0 | 0 | 0 | 0 |
| 778 | InChI=1S/C6H8O7/c7-1-3(9)4(10)6(13,2-8)5(11)12/h7-8,13H,1-2H2,(H,11,12) | 0 | 0 | 1 | 0 | 0 | 0 | 0 |
| 779 | InChI=1S/C6H8O7/c7-1-3(9)6(13,2-8)4(10)5(11)12/h7-8,13H,1-2H2,(H,11,12) | 0 | 0 | 1 | 0 | 0 | 0 | 0 |
| 780 | InChI=1S/C6H8O7/c7-1-6(2-8,5(12)13)3(9)4(10)11/h7-8H,1-2H2,(H,10,11)(H,12,13) | 0 | 0 | 1 | 0 | 0 | 0 | 0 |
| 781 | InChI=1S/C6H8O7/c7-2-1-6(13,5(11)12)3(8)4(9)10/h7,13H,1-2H2,(H,9,10)(H,11,12) | 0 | 0 | 1 | 0 | 0 | 0 | 0 |
| 782 | InChI=1S/C6H8O7/c7-2-6(13,1-3(8)9)4(10)5(11)12/h7,13H,1-2H2,(H,8,9)(H,11,12) | 0 | 0 | 1 | 0 | 0 | 0 | 0 |
| 783 | InChI=1S/C6H8O7/c7-2-6(13,5(11)12)1-3(8)4(9)10/h7,13H,1-2H2,(H,9,10)(H,11,12) | 0 | 0 | 1 | 0 | 1 | 0 | 0 |
| 784 | InChI=1S/C6H8O7/c7-1-3(9)6(13,5(11)12)4(10)2-8/h7-8,13H,1-2H2,(H,11,12) | 0 | 0 | 1 | 0 | 0 | 0 | 0 |
| 785 | InChI=1S/C6H8O7/c7-1-3(9)6(2-8,4(10)11)5(12)13/h7-8H,1-2H2,(H,10,11)(H,12,13) | 0 | 0 | 1 | 0 | 0 | 0 | 0 |
| 786 | InChI=1S/C6H8O7/c7-2-1-3(8)6(13,4(9)10)5(11)12/h7,13H,1-2H2,(H,9,10)(H,11,12) | 0 | 0 | 1 | 0 | 0 | 0 | 0 |
| 787 | InChI=1S/C6H8O7/c7-2-6(13,5(11)12)3(8)1-4(9)10/h7,13H,1-2H2,(H,9,10)(H,11,12) | 0 | 0 | 1 | 0 | 0 | 0 | 0 |
| 788 | InChI=1S/C6H8O7/c7-2-3(8)6(13,5(11)12)1-4(9)10/h7,13H,1-2H2,(H,9,10)(H,11,12) | 0 | 0 | 1 | 0 | 0 | 0 | 0 |
| 789 | InChI=1S/C6H8O7/c7-2-1-6(3(8)9,4(10)11)5(12)13/h7H,1-2H2,(H,8,9)(H,10,11)(H,12,13) | 0 | 0 | 1 | 0 | 1 | 0 | 0 |
| 790 | InChI=1S/C6H8O7/c7-2-3(8)1-6(13,4(9)10)5(11)12/h7,13H,1-2H2,(H,9,10)(H,11,12) | 0 | 0 | 1 | 0 | 0 | 0 | 0 |
| 791 | InChI=1S/C6H8O7/c7-2-6(4(10)11,5(12)13)1-3(8)9/h7H,1-2H2,(H,8,9)(H,10,11)(H,12,13) | 0 | 0 | 1 | 0 | 1 | 0 | 0 |
| 792 | InChI=1S/C6H8O7/c7-3(8)1-2-6(13,4(9)10)5(11)12/h13H,1-2H2,(H,7,8)(H,9,10)(H,11,12) | 0 | 0 | 1 | 0 | 1 | 1 | 0 |
| 793 | InChI=1S/C6H8O7/c7-3(8)1-6(13,5(11)12)2-4(9)10/h13H,1-2H2,(H,7,8)(H,9,10)(H,11,12) | 1 | 1 | 1 | 1 | 1 | 1 | 1 |
| 794 | InChI=1S/C6H8O7/c7-1-5(12,2-8)6(13,3-9)4(10)11/h1,3,8,12-13H,2H2,(H,10,11) | 0 | 0 | 1 | 0 | 0 | 0 | 0 |
| 795 | InChI=1S/C6H8O7/c7-1-5(12,2-8)6(13,3-9)4(10)11/h1-2,9,12-13H,3H2,(H,10,11) | 0 | 0 | 1 | 0 | 0 | 0 | 0 |
| 796 | InChI=1S/C6H8O7/c1-5(12,2-7)6(13,3(8)9)4(10)11/h2,12-13H,1H3,(H,8,9)(H,10,11) | 0 | 0 | 1 | 0 | 0 | 0 | 0 |
| 797 | InChI=1S/C6H8O7/c1-5(12,3(8)9)6(13,2-7)4(10)11/h2,12-13H,1H3,(H,8,9)(H,10,11) | 0 | 0 | 1 | 0 | 0 | 0 | 0 |
| 798 | InChI=1S/C6H8O8/c7-1-2(8)3(9)6(14,4(10)11)5(12)13/h1-3,8-9,14H,(H,10,11)(H,12,13) | 0 | 0 | 1 | 0 | 0 | 0 | 0 |
| 799 | InChI=1S/C6H8O8/c7-1-6(14,5(12)13)3(9)2(8)4(10)11/h1-3,8-9,14H,(H,10,11)(H,12,13) | 0 | 0 | 1 | 0 | 0 | 0 | 0 |
| 800 | InChI=1S/C6H8O8/c7-1-2(8)6(14,5(12)13)3(9)4(10)11/h1-3,8-9,14H,(H,10,11)(H,12,13) | 0 | 0 | 1 | 0 | 0 | 0 | 0 |
| 801 | InChI=1S/C6H8O8/c7-1-6(14,2(8)4(10)11)3(9)5(12)13/h1-3,8-9,14H,(H,10,11)(H,12,13) | 0 | 0 | 1 | 0 | 0 | 0 | 0 |
| 802 | InChI=1S/C6H8O8/c7-1-2(8)6(14,5(12)13)3(9)4(10)11/h2,7-8,14H,1H2,(H,10,11)(H,12,13) | 0 | 0 | 1 | 0 | 0 | 0 | 0 |
| 803 | InChI=1S/C6H8O8/c7-1-6(14,2(8)4(10)11)3(9)5(12)13/h2,7-8,14H,1H2,(H,10,11)(H,12,13) | 0 | 0 | 1 | 0 | 0 | 0 | 0 |
| 804 | InChI=1S/C6H8O8/c7-1-6(14,5(12)13)3(9)2(8)4(10)11/h3,7,9,14H,1H2,(H,10,11)(H,12,13) | 0 | 0 | 1 | 0 | 0 | 0 | 0 |
| 805 | InChI=1S/C6H8O8/c7-1-2(8)3(9)6(14,4(10)11)5(12)13/h2,7-8,14H,1H2,(H,10,11)(H,12,13) | 0 | 0 | 1 | 0 | 0 | 0 | 0 |
| 806 | InChI=1S/C6H8O8/c7-1-6(14,5(12)13)3(9)2(8)4(10)11/h2,7-8,14H,1H2,(H,10,11)(H,12,13) | 0 | 0 | 1 | 0 | 0 | 0 | 0 |
| 807 | InChI=1S/C6H8O8/c7-1-2(8)6(14,5(12)13)3(9)4(10)11/h3,7,9,14H,1H2,(H,10,11)(H,12,13) | 0 | 0 | 1 | 0 | 0 | 0 | 0 |
| 808 | InChI=1S/C6H8O8/c7-1-2(8)6(3(9)10,4(11)12)5(13)14/h2,7-8H,1H2,(H,9,10)(H,11,12)(H,13,14) | 0 | 0 | 1 | 0 | 1 | 0 | 0 |
| 809 | InChI=1S/C6H8O8/c7-1-2(8)3(9)6(14,4(10)11)5(12)13/h3,7,9,14H,1H2,(H,10,11)(H,12,13) | 0 | 0 | 1 | 0 | 0 | 0 | 0 |
| 810 | InChI=1S/C6H8O8/c7-1-6(4(11)12,5(13)14)2(8)3(9)10/h2,7-8H,1H2,(H,9,10)(H,11,12)(H,13,14) | 0 | 0 | 1 | 0 | 0 | 0 | 0 |
| 811 | InChI=1S/C6H8O8/c7-1-2(3(8)9)6(14,4(10)11)5(12)13/h2,7,14H,1H2,(H,8,9)(H,10,11)(H,12,13) | 0 | 0 | 1 | 0 | 0 | 0 | 0 |
| 812 | InChI=1S/C6H8O8/c7-2(3(8)9)1-6(14,4(10)11)5(12)13/h2,7,14H,1H2,(H,8,9)(H,10,11)(H,12,13) | 1 | 0 | 1 | 0 | 1 | 1 | 0 |
| 813 | InChI=1S/C6H8O8/c7-2(1-3(8)9)6(14,4(10)11)5(12)13/h2,7,14H,1H2,(H,8,9)(H,10,11)(H,12,13) | 0 | 0 | 1 | 0 | 1 | 0 | 0 |
| 814 | InChI=1S/C6H8O8/c7-1-6(14,5(12)13)2(3(8)9)4(10)11/h2,7,14H,1H2,(H,8,9)(H,10,11)(H,12,13) | 0 | 0 | 1 | 0 | 1 | 0 | 0 |
| 815 | InChI=1S/C6H8O8/c7-2(8)1-6(14,5(12)13)3(9)4(10)11/h3,9,14H,1H2,(H,7,8)(H,10,11)(H,12,13) | 1 | 1 | 1 | 0 | 1 | 1 | 0 |
| 816 | InChI=1S/C6H8O8/c7-1(3(9)5(11)12)2(8)4(10)6(13)14/h1-3,7-9H,(H,11,12)(H,13,14) | 1 | 0 | 1 | 0 | 1 | 1 | 0 |
| 817 | InChI=1S/C6H8O8/c7-1(3(9)5(11)12)2(8)4(10)6(13)14/h1,3-4,7,9-10H,(H,11,12)(H,13,14) | 0 | 0 | 1 | 0 | 1 | 0 | 0 |
| 818 | InChI=1S/C6H8O8/c7-2(3(8)6(13)14)1(4(9)10)5(11)12/h1-3,7-8H,(H,9,10)(H,11,12)(H,13,14) | 0 | 0 | 1 | 0 | 1 | 0 | 0 |
| 819 | InChI=1S/C6H8O8/c7-2(5(11)12)1(4(9)10)3(8)6(13)14/h1-3,7-8H,(H,9,10)(H,11,12)(H,13,14) | 0 | 0 | 1 | 0 | 1 | 1 | 0 |
| 820 | InChI=1S/C6H8O8/c7-1-5(13,2-8)6(14,3(9)10)4(11)12/h1,8,13-14H,2H2,(H,9,10)(H,11,12) | 0 | 0 | 1 | 0 | 0 | 0 | 0 |
| 821 | InChI=1S/C6H8O8/c7-1-5(13,3(9)10)6(14,2-8)4(11)12/h1,8,13-14H,2H2,(H,9,10)(H,11,12) | 0 | 0 | 1 | 0 | 0 | 0 | 0 |
| 822 | InChI=1S/C6H8O8/c1-5(13,2(7)8)6(14,3(9)10)4(11)12/h13-14H,1H3,(H,7,8)(H,9,10)(H,11,12) | 0 | 0 | 1 | 0 | 1 | 0 | 0 |
| 823 | InChI=1S/C6H8O9/c7-1(3(9)10)2(8)6(15,4(11)12)5(13)14/h1-2,7-8,15H,(H,9,10)(H,11,12)(H,13,14) | 1 | 0 | 1 | 0 | 1 | 1 | 0 |
| 824 | InChI=1S/C6H8O9/c7-1(3(9)10)6(15,5(13)14)2(8)4(11)12/h1-2,7-8,15H,(H,9,10)(H,11,12)(H,13,14) | 0 | 0 | 1 | 0 | 1 | 0 | 0 |
| 825 | InChI=1S/C6H8O9/c7-1-5(14,2(8)9)6(15,3(10)11)4(12)13/h7,14-15H,1H2,(H,8,9)(H,10,11)(H,12,13) | 0 | 0 | 1 | 0 | 1 | 0 | 0 |
| 826 | InChI=1S/C6H10O7/c7-1-3(9)4(10)6(13,2-8)5(11)12/h2-4,7,9-10,13H,1H2,(H,11,12) | 0 | 0 | 1 | 0 | 1 | 0 | 0 |
| 827 | InChI=1S/C6H10O7/c7-1-3(9)6(13,5(11)12)4(10)2-8/h1,3-4,8-10,13H,2H2,(H,11,12) | 0 | 0 | 1 | 0 | 0 | 0 | 0 |
| 828 | InChI=1S/C6H10O7/c7-1-3(9)4(10)6(13,2-8)5(11)12/h1,3-4,8-10,13H,2H2,(H,11,12) | 1 | 0 | 1 | 0 | 0 | 1 | 0 |
| 829 | InChI=1S/C6H10O7/c7-1-3(9)6(13,2-8)4(10)5(11)12/h2-4,7,9-10,13H,1H2,(H,11,12) | 0 | 0 | 1 | 0 | 0 | 0 | 0 |
| 830 | InChI=1S/C6H10O7/c7-1-6(13,2-8)4(10)3(9)5(11)12/h1,3-4,8-10,13H,2H2,(H,11,12) | 0 | 0 | 1 | 0 | 1 | 0 | 0 |
| 831 | InChI=1S/C6H10O7/c7-1-3(9)6(13,2-8)4(10)5(11)12/h1,3-4,8-10,13H,2H2,(H,11,12) | 0 | 0 | 1 | 0 | 1 | 0 | 0 |
| 832 | InChI=1S/C6H10O7/c7-1-2(8)3(9)4(10)5(11)6(12)13/h1-5,8-11H,(H,12,13) | 1 | 0 | 1 | 1 | 1 | 1 | 0 |
| 833 | InChI=1S/C6H10O7/c7-1-2(8)3(9)4(10)5(11)6(12)13/h2-4,7-10H,1H2,(H,12,13) | 1 | 0 | 1 | 1 | 1 | 1 | 0 |
| 834 | InChI=1S/C6H10O7/c7-1-2(8)3(9)4(10)5(11)6(12)13/h2-3,5,7-9,11H,1H2,(H,12,13) | 0 | 0 | 1 | 0 | 1 | 1 | 0 |
| 835 | InChI=1S/C6H10O7/c7-1-2(8)3(9)4(10)5(11)6(12)13/h2,4-5,7-8,10-11H,1H2,(H,12,13) | 1 | 0 | 1 | 0 | 1 | 1 | 0 |
| 836 | InChI=1S/C6H10O7/c7-1-2(8)3(9)4(10)5(11)6(12)13/h3-5,7,9-11H,1H2,(H,12,13) | 1 | 0 | 1 | 1 | 1 | 1 | 0 |
| 837 | InChI=1S/C6H10O7/c7-1-2(8)4(9)3(5(10)11)6(12)13/h2-4,7-9H,1H2,(H,10,11)(H,12,13) | 0 | 0 | 1 | 0 | 1 | 0 | 0 |
| 838 | InChI=1S/C6H10O7/c7-1-2(8)3(5(10)11)4(9)6(12)13/h2-4,7-9H,1H2,(H,10,11)(H,12,13) | 0 | 0 | 1 | 0 | 0 | 0 | 0 |
| 839 | InChI=1S/C6H10O7/c7-1-2(5(10)11)3(8)4(9)6(12)13/h2-4,7-9H,1H2,(H,10,11)(H,12,13) | 0 | 0 | 1 | 0 | 0 | 0 | 0 |
| 840 | InChI=1S/C6H10O7/c7-1-2(3(8)5(10)11)4(9)6(12)13/h2-4,7-9H,1H2,(H,10,11)(H,12,13) | 0 | 0 | 1 | 0 | 0 | 0 | 0 |
| 841 | InChI=1S/C6H10O7/c7-2(4(9)6(12)13)1-3(8)5(10)11/h2-4,7-9H,1H2,(H,10,11)(H,12,13) | 1 | 0 | 1 | 0 | 1 | 1 | 0 |
| 842 | InChI=1S/C6H10O7/c7-2(1-3(8)9)4(10)5(11)6(12)13/h2,4-5,7,10-11H,1H2,(H,8,9)(H,12,13) | 1 | 0 | 1 | 0 | 1 | 1 | 0 |
| 843 | InChI=1S/C6H10O7/c1-2(7)3(8)6(13,4(9)10)5(11)12/h2-3,7-8,13H,1H3,(H,9,10)(H,11,12) | 0 | 0 | 1 | 0 | 0 | 0 | 0 |
| 844 | InChI=1S/C6H10O7/c1-2(7)6(13,5(11)12)3(8)4(9)10/h2-3,7-8,13H,1H3,(H,9,10)(H,11,12) | 0 | 0 | 1 | 0 | 0 | 0 | 0 |
| 845 | InChI=1S/C6H10O7/c1-6(13,5(11)12)3(8)2(7)4(9)10/h2-3,7-8,13H,1H3,(H,9,10)(H,11,12) | 1 | 0 | 1 | 0 | 1 | 1 | 0 |
| 846 | InChI=1S/C6H10O7/c1-6(13,2(7)4(9)10)3(8)5(11)12/h2-3,7-8,13H,1H3,(H,9,10)(H,11,12) | 0 | 0 | 1 | 0 | 1 | 0 | 0 |
| 847 | InChI=1S/C6H10O7/c7-1-5(12,2-8)6(13,3-9)4(10)11/h3,7-8,12-13H,1-2H2,(H,10,11) | 0 | 0 | 1 | 0 | 0 | 0 | 0 |
| 848 | InChI=1S/C6H10O7/c7-1-5(12,2-8)6(13,3-9)4(10)11/h1,8-9,12-13H,2-3H2,(H,10,11) | 0 | 0 | 1 | 0 | 0 | 0 | 0 |
| 849 | InChI=1S/C6H10O7/c7-1-3(9)6(13,2-8)4(10)5(11)12/h3,7-9,13H,1-2H2,(H,11,12) | 0 | 0 | 1 | 0 | 0 | 0 | 0 |
| 850 | InChI=1S/C6H10O7/c7-1-6(13,2-8)4(10)3(9)5(11)12/h4,7-8,10,13H,1-2H2,(H,11,12) | 0 | 0 | 1 | 0 | 0 | 0 | 0 |
| 851 | InChI=1S/C6H10O7/c7-1-3(9)4(10)6(13,2-8)5(11)12/h3,7-9,13H,1-2H2,(H,11,12) | 0 | 0 | 1 | 0 | 0 | 0 | 0 |
| 852 | InChI=1S/C6H10O7/c7-1-6(13,2-8)4(10)3(9)5(11)12/h3,7-9,13H,1-2H2,(H,11,12) | 0 | 0 | 1 | 0 | 0 | 0 | 0 |
| 853 | InChI=1S/C6H10O7/c7-1-3(9)6(13,5(11)12)4(10)2-8/h3,7-9,13H,1-2H2,(H,11,12) | 0 | 0 | 1 | 0 | 1 | 0 | 0 |
| 854 | InChI=1S/C6H10O7/c7-1-3(9)6(13,2-8)4(10)5(11)12/h4,7-8,10,13H,1-2H2,(H,11,12) | 0 | 0 | 1 | 0 | 1 | 0 | 0 |
| 855 | InChI=1S/C6H10O7/c7-1-3(9)6(2-8,4(10)11)5(12)13/h3,7-9H,1-2H2,(H,10,11)(H,12,13) | 0 | 0 | 1 | 0 | 0 | 0 | 0 |
| 856 | InChI=1S/C6H10O7/c7-1-3(2-8)6(13,4(9)10)5(11)12/h3,7-8,13H,1-2H2,(H,9,10)(H,11,12) | 0 | 0 | 1 | 0 | 0 | 0 | 0 |
| 857 | InChI=1S/C6H10O7/c7-2-1-3(8)6(13,4(9)10)5(11)12/h3,7-8,13H,1-2H2,(H,9,10)(H,11,12) | 0 | 0 | 1 | 0 | 0 | 0 | 0 |
| 858 | InChI=1S/C6H10O7/c7-2-3(8)1-6(13,4(9)10)5(11)12/h3,7-8,13H,1-2H2,(H,9,10)(H,11,12) | 0 | 0 | 1 | 0 | 1 | 0 | 0 |
| 859 | InChI=1S/C6H10O7/c7-1-3(9)4(10)6(13,2-8)5(11)12/h4,7-8,10,13H,1-2H2,(H,11,12) | 0 | 0 | 1 | 0 | 0 | 0 | 0 |
| 860 | InChI=1S/C6H10O7/c7-1-6(2-8,5(12)13)3(9)4(10)11/h3,7-9H,1-2H2,(H,10,11)(H,12,13) | 0 | 0 | 1 | 0 | 0 | 0 | 0 |
| 861 | InChI=1S/C6H10O7/c7-1-3(4(9)10)6(13,2-8)5(11)12/h3,7-8,13H,1-2H2,(H,9,10)(H,11,12) | 0 | 0 | 1 | 0 | 0 | 0 | 0 |
| 862 | InChI=1S/C6H10O7/c7-2-1-6(13,5(11)12)3(8)4(9)10/h3,7-8,13H,1-2H2,(H,9,10)(H,11,12) | 1 | 0 | 1 | 0 | 0 | 1 | 0 |
| 863 | InChI=1S/C6H10O7/c7-2-6(13,5(11)12)1-3(8)4(9)10/h3,7-8,13H,1-2H2,(H,9,10)(H,11,12) | 1 | 0 | 1 | 0 | 1 | 1 | 0 |
| 864 | InChI=1S/C6H10O7/c7-2-3(8)6(13,5(11)12)1-4(9)10/h3,7-8,13H,1-2H2,(H,9,10)(H,11,12) | 1 | 0 | 1 | 0 | 0 | 1 | 0 |
| 865 | InChI=1S/C6H10O7/c7-2-6(13,5(11)12)3(8)1-4(9)10/h3,7-8,13H,1-2H2,(H,9,10)(H,11,12) | 0 | 0 | 1 | 0 | 0 | 0 | 0 |
| 866 | InChI=1S/C6H10O7/c7-1-6(13,2-8)3(4(9)10)5(11)12/h3,7-8,13H,1-2H2,(H,9,10)(H,11,12) | 0 | 0 | 1 | 0 | 0 | 0 | 0 |
| 867 | InChI=1S/C6H10O7/c7-2-6(13,1-3(8)9)4(10)5(11)12/h4,7,10,13H,1-2H2,(H,8,9)(H,11,12) | 0 | 0 | 1 | 0 | 0 | 0 | 0 |
| 868 | InChI=1S/C6H10O7/c1-5(12,2-7)6(13,3(8)9)4(10)11/h7,12-13H,2H2,1H3,(H,8,9)(H,10,11) | 0 | 0 | 1 | 0 | 0 | 0 | 0 |
| 869 | InChI=1S/C6H10O7/c1-5(12,3(8)9)6(13,2-7)4(10)11/h7,12-13H,2H2,1H3,(H,8,9)(H,10,11) | 0 | 0 | 1 | 0 | 0 | 0 | 0 |
| 870 | InChI=1S/C6H10O8/c7-1-2(8)3(9)6(14,4(10)11)5(12)13/h2-3,7-9,14H,1H2,(H,10,11)(H,12,13) | 0 | 0 | 1 | 0 | 1 | 0 | 0 |
| 871 | InChI=1S/C6H10O8/c7-1-2(8)6(14,5(12)13)3(9)4(10)11/h2-3,7-9,14H,1H2,(H,10,11)(H,12,13) | 0 | 0 | 1 | 0 | 0 | 0 | 0 |
| 872 | InChI=1S/C6H10O8/c7-1-6(14,5(12)13)3(9)2(8)4(10)11/h2-3,7-9,14H,1H2,(H,10,11)(H,12,13) | 1 | 0 | 1 | 0 | 1 | 1 | 0 |
| 873 | InChI=1S/C6H10O8/c7-1-6(14,2(8)4(10)11)3(9)5(12)13/h2-3,7-9,14H,1H2,(H,10,11)(H,12,13) | 0 | 0 | 1 | 0 | 0 | 0 | 0 |
| 874 | InChI=1S/C6H10O8/c7-1(3(9)5(11)12)2(8)4(10)6(13)14/h1-4,7-10H,(H,11,12)(H,13,14) | 1 | 0 | 1 | 1 | 1 | 1 | 0 |
| 875 | InChI=1S/C6H10O8/c7-1-5(13,2-8)6(14,3(9)10)4(11)12/h7-8,13-14H,1-2H2,(H,9,10)(H,11,12) | 0 | 0 | 1 | 0 | 0 | 0 | 0 |
| 876 | InChI=1S/C6H10O8/c7-1-5(13,3(9)10)6(14,2-8)4(11)12/h7-8,13-14H,1-2H2,(H,9,10)(H,11,12) | 0 | 0 | 1 | 0 | 0 | 0 | 0 |
| 877 | InChI=1S/C2H4O3/c3-1-2(4)5/h1-2,4-5H | 1 | 1 | 0 | 0 | 0 | 0 | 0 |
| 878 | InChI=1S/C2H4O4/c3-1(4)2(5)6/h1,3-4H,(H,5,6) | 1 | 1 | 0 | 0 | 0 | 0 | 0 |
| 879 | InChI=1S/C3H4O3/c4-2-1-3(5)6/h1-2,4H,(H,5,6) | 1 | 1 | 0 | 0 | 0 | 0 | 0 |
| 880 | InChI=1S/C3H4O3/c4-1-3(6)2-5/h1-2,4,6H | 1 | 0 | 0 | 0 | 0 | 0 | 0 |
| 881 | InChI=1S/C3H4O3/c1-2(4)3(5)6/h4H,1H2,(H,5,6) | 1 | 1 | 0 | 0 | 0 | 0 | 0 |
| 882 | InChI=1S/C3H4O4/c4-2(5)1-3(6)7/h1,4-5H,(H,6,7) | 1 | 0 | 0 | 0 | 0 | 0 | 0 |
| 883 | InChI=1S/C3H4O4/c4-1-2(5)3(6)7/h1,4-5H,(H,6,7) | 1 | 0 | 0 | 0 | 0 | 0 | 0 |
| 884 | InChI=1S/C3H4O4/c4-1-3(6,7)2-5/h1-2,6-7H | 1 | 0 | 0 | 0 | 0 | 0 | 0 |
| 885 | InChI=1S/C3H4O6/c4-1(5)3(8,9)2(6)7/h8-9H,(H,4,5)(H,6,7) | 1 | 1 | 0 | 0 | 0 | 0 | 0 |
| 886 | InChI=1S/C3H6O3/c1-2(4)3(5)6/h3,5-6H,1H3 | 1 | 1 | 0 | 0 | 0 | 0 | 0 |
| 887 | InChI=1S/C3H6O4/c1-3(6,7)2(4)5/h6-7H,1H3,(H,4,5) | 1 | 1 | 0 | 0 | 0 | 0 | 0 |
| 888 | InChI=1S/C4H2O4/c5-1-3(7)4(8)2-6/h7-8H | 1 | 0 | 0 | 0 | 0 | 0 | 0 |
| 889 | InChI=1S/C4H4O4/c5-2-1-3(6)4(7)8/h1-2,6H,(H,7,8) | 1 | 0 | 0 | 0 | 0 | 0 | 0 |
| 890 | InChI=1S/C4H4O4/c5-2-1-3(6)4(7)8/h1-2,5H,(H,7,8) | 1 | 0 | 0 | 0 | 0 | 0 | 0 |
| 891 | InChI=1S/C4H4O5/c5-2(4(8)9)1-3(6)7/h1,5H,(H,6,7)(H,8,9) | 1 | 1 | 0 | 0 | 0 | 0 | 0 |
| 892 | InChI=1S/C4H4O6/c5-1(3(7)8)2(6)4(9)10/h5-6H,(H,7,8)(H,9,10) | 1 | 0 | 0 | 0 | 0 | 0 | 0 |
| 893 | InChI=1S/C4H6O4/c1-2(3(5)6)4(7)8/h5-6H,1H3,(H,7,8) | 1 | 0 | 0 | 0 | 0 | 0 | 0 |
| 894 | InChI=1S/C4H6O5/c5-1-2(6)3(7)4(8)9/h5-7H,1H2,(H,8,9) | 1 | 0 | 0 | 0 | 0 | 0 | 0 |
| 895 | InChI=1S/C4H6O6/c5-2(6)1-4(9,10)3(7)8/h9-10H,1H2,(H,5,6)(H,7,8) | 1 | 1 | 0 | 0 | 0 | 0 | 0 |
| 896 | InChI=1S/C4H6O8/c5-1(6)3(9,10)4(11,12)2(7)8/h9-12H,(H,5,6)(H,7,8) | 1 | 0 | 0 | 0 | 0 | 0 | 0 |
| 897 | InChI=1S/C5H4O7/c6-1(2(7)4(9)10)3(8)5(11)12/h6-7H,(H,9,10)(H,11,12) | 1 | 0 | 0 | 0 | 0 | 0 | 0 |
| 898 | InChI=1S/C5H4O7/c6-2(5(11)12)1(3(7)8)4(9)10/h6H,(H,7,8)(H,9,10)(H,11,12) | 1 | 0 | 0 | 0 | 0 | 0 | 0 |
| 899 | InChI=1S/C5H6O5/c1-2(4(7)8)3(6)5(9)10/h6H,1H3,(H,7,8)(H,9,10) | 1 | 0 | 0 | 0 | 0 | 0 | 0 |
| 900 | InChI=1S/C5H6O6/c6-1-2(7)3(8)4(9)5(10)11/h6,8-9H,1H2,(H,10,11) | 1 | 0 | 0 | 0 | 0 | 0 | 0 |
| 901 | InChI=1S/C5H6O7/c6-1(2(7)4(9)10)3(8)5(11)12/h2,6-8H,(H,9,10)(H,11,12) | 1 | 0 | 0 | 0 | 0 | 0 | 0 |
| 902 | InChI=1S/C5H8O6/c6-3(7)1-2-5(10,11)4(8)9/h10-11H,1-2H2,(H,6,7)(H,8,9) | 1 | 0 | 0 | 0 | 0 | 0 | 0 |
| 903 | InChI=1S/C5H8O6/c1-2(3(6)7)5(10,11)4(8)9/h2,10-11H,1H3,(H,6,7)(H,8,9) | 1 | 0 | 0 | 0 | 0 | 0 | 0 |
| 904 | InChI=1S/C6H6O6/c7-3(5(9)10)1-2-4(8)6(11)12/h1-2,7-8H,(H,9,10)(H,11,12) | 1 | 0 | 0 | 0 | 0 | 0 | 0 |
| 905 | InChI=1S/C6H6O6/c7-2-3(8)1-4(9)5(10)6(11)12/h1,7,9H,2H2,(H,11,12) | 1 | 0 | 0 | 0 | 0 | 0 | 0 |
| 906 | InChI=1S/C6H6O7/c7-3(8)1-2(5(10)11)4(9)6(12)13/h9H,1H2,(H,7,8)(H,10,11)(H,12,13) | 1 | 0 | 0 | 0 | 0 | 0 | 0 |
| 907 | InChI=1S/C6H8O7/c7-1-2(8)3(9)4(10)5(11)6(12)13/h2,7-8,10-11H,1H2,(H,12,13) | 1 | 0 | 0 | 0 | 0 | 0 | 0 |
| 908 | InChI=1S/C6H10O7/c7-1-2(8)3(9)4(10)5(11)6(12)13/h2-3,7-11H,1H2,(H,12,13) | 1 | 0 | 0 | 0 | 0 | 0 | 0 |
| 909 | InChI=1S/C6H10O8/c7-1-6(13,14)4(10)2(8)3(9)5(11)12/h2,4,7-8,10,13-14H,1H2,(H,11,12) | 1 | 0 | 0 | 0 | 0 | 0 | 0 |
| 910 | InChI=1S/C6H10O10/c7-1(5(13,14)3(9)10)2(8)6(15,16)4(11)12/h1-2,7-8,13-16H,(H,9,10)(H,11,12) | 1 | 0 | 0 | 0 | 0 | 0 | 0 |
| 911 | InChI=1S/CH4O/c1-2/h2H,1H3 | 0 | 1 | 0 | 0 | 0 | 0 | 0 |
| 912 | InChI=1S/CH4O2/c2-1-3/h2-3H,1H2 | 0 | 1 | 0 | 0 | 0 | 0 | 0 |
| 913 | InChI=1S/C2H4/c1-2/h1-2H2 | 0 | 1 | 0 | 0 | 0 | 0 | 0 |
| 914 | InChI=1S/C2H4O/c1-2-3/h2-3H,1H2 | 0 | 1 | 0 | 0 | 0 | 0 | 0 |
| 915 | InChI=1S/C2H4O/c1-2-3/h2H,1H3 | 0 | 1 | 0 | 0 | 0 | 0 | 0 |
| 916 | InChI=1S/C2H4O2/c1-2(3)4/h3-4H,1H2 | 0 | 1 | 0 | 0 | 0 | 0 | 0 |
| 917 | InChI=1S/C2H4O3/c3-1-2(4)5/h1,3-5H | 0 | 1 | 0 | 0 | 0 | 0 | 0 |
| 918 | InChI=1S/C2H6/c1-2/h1-2H3 | 0 | 1 | 0 | 0 | 0 | 0 | 0 |
| 919 | InChI=1S/C2H6O/c1-2-3/h3H,2H2,1H3 | 0 | 1 | 0 | 0 | 0 | 0 | 0 |
| 920 | InChI=1S/C2H6O2/c1-2(3)4/h2-4H,1H3 | 0 | 1 | 0 | 0 | 0 | 0 | 0 |
| 921 | InChI=1S/C2H6O2/c3-1-2-4/h3-4H,1-2H2 | 0 | 1 | 0 | 0 | 0 | 0 | 0 |
| 922 | InChI=1S/C2H6O3/c3-1-2(4)5/h2-5H,1H2 | 0 | 1 | 0 | 0 | 0 | 0 | 0 |
| 923 | InChI=1S/C2H6O3/c1-2(3,4)5/h3-5H,1H3 | 0 | 1 | 0 | 0 | 0 | 0 | 0 |
| 924 | InChI=1S/C2H6O4/c3-1(4)2(5)6/h1-6H | 0 | 1 | 0 | 0 | 0 | 0 | 0 |
| 925 | InChI=1S/C3H4O2/c1-2-3(4)5/h2H,1H2,(H,4,5) | 0 | 1 | 0 | 0 | 0 | 0 | 0 |
| 926 | InChI=1S/C3H4O5/c4-1(2(5)6)3(7)8/h2,5-6H,(H,7,8) | 0 | 1 | 0 | 0 | 0 | 0 | 0 |
| 927 | InChI=1S/C3H6/c1-3-2/h3H,1H2,2H3 | 0 | 1 | 0 | 0 | 0 | 0 | 0 |
| 928 | InChI=1S/C3H6O/c1-2-3-4/h3H,2H2,1H3 | 0 | 1 | 0 | 0 | 0 | 0 | 0 |
| 929 | InChI=1S/C3H6O/c1-2-3-4/h2,4H,1,3H2 | 0 | 1 | 0 | 0 | 0 | 0 | 0 |
| 930 | InChI=1S/C3H6O/c1-3(2)4/h4H,1H2,2H3 | 0 | 1 | 0 | 0 | 0 | 0 | 0 |
| 931 | InChI=1S/C3H6O2/c1-2-3(4)5/h2-5H,1H2 | 0 | 1 | 0 | 0 | 0 | 0 | 0 |
| 932 | InChI=1S/C3H6O2/c1-2-3(4)5/h2H2,1H3,(H,4,5) | 0 | 1 | 0 | 0 | 0 | 0 | 0 |
| 933 | InChI=1S/C3H6O2/c4-2-1-3-5/h1-2,4-5H,3H2 | 0 | 1 | 0 | 0 | 0 | 0 | 0 |
| 934 | InChI=1S/C3H6O2/c1-3(5)2-4/h4-5H,1-2H2 | 0 | 1 | 0 | 0 | 0 | 0 | 0 |
| 935 | InChI=1S/C3H6O4/c4-2(5)1-3(6)7/h2,4-5H,1H2,(H,6,7) | 0 | 1 | 0 | 0 | 0 | 0 | 0 |
| 936 | InChI=1S/C3H8/c1-3-2/h3H2,1-2H3 | 0 | 1 | 0 | 0 | 0 | 0 | 0 |
| 937 | InChI=1S/C3H8O/c1-2-3-4/h4H,2-3H2,1H3 | 0 | 1 | 0 | 0 | 0 | 0 | 0 |
| 938 | InChI=1S/C3H8O/c1-3(2)4/h3-4H,1-2H3 | 0 | 1 | 0 | 0 | 0 | 0 | 0 |
| 939 | InChI=1S/C3H8O2/c1-2-3(4)5/h3-5H,2H2,1H3 | 0 | 1 | 0 | 0 | 0 | 0 | 0 |
| 940 | InChI=1S/C3H8O2/c4-2-1-3-5/h4-5H,1-3H2 | 0 | 1 | 0 | 0 | 0 | 0 | 0 |
| 941 | InChI=1S/C3H8O2/c1-3(5)2-4/h3-5H,2H2,1H3 | 0 | 1 | 0 | 0 | 0 | 0 | 0 |
| 942 | InChI=1S/C3H8O2/c1-3(2,4)5/h4-5H,1-2H3 | 0 | 1 | 0 | 0 | 0 | 0 | 0 |
| 943 | InChI=1S/C3H8O3/c1-2(4)3(5)6/h2-6H,1H3 | 0 | 1 | 0 | 0 | 0 | 0 | 0 |
| 944 | InChI=1S/C3H8O3/c4-2-1-3(5)6/h3-6H,1-2H2 | 0 | 1 | 0 | 0 | 0 | 0 | 0 |
| 945 | InChI=1S/C3H8O3/c4-1-3(6)2-5/h3-6H,1-2H2 | 0 | 1 | 0 | 0 | 0 | 0 | 0 |
| 946 | InChI=1S/C3H8O4/c4-2(5)1-3(6)7/h2-7H,1H2 | 0 | 1 | 0 | 0 | 0 | 0 | 0 |
| 947 | InChI=1S/C3H8O4/c4-1-2(5)3(6)7/h2-7H,1H2 | 0 | 1 | 0 | 0 | 0 | 0 | 0 |
| 948 | InChI=1S/C4H4O3/c1-2-3(5)4(6)7/h2H,1H2,(H,6,7) | 0 | 1 | 0 | 0 | 0 | 0 | 0 |
| 949 | InChI=1S/C4H4O3/c5-3-1-2-4(6)7/h1-3H,(H,6,7) | 0 | 1 | 0 | 0 | 0 | 0 | 0 |
| 950 | InChI=1S/C4H4O5/c5-1-2(3(6)7)4(8)9/h1,5H,(H,6,7)(H,8,9) | 0 | 1 | 0 | 0 | 0 | 0 | 0 |
| 951 | InChI=1S/C4H6/c1-3-4-2/h3-4H,1-2H2 | 0 | 1 | 0 | 0 | 0 | 0 | 0 |
| 952 | InChI=1S/C4H6O/c1-3-4(2)5/h3,5H,1-2H2 | 0 | 1 | 0 | 0 | 0 | 0 | 0 |
| 953 | InChI=1S/C4H6O2/c1-3(2)4(5)6/h1H2,2H3,(H,5,6) | 0 | 1 | 0 | 0 | 0 | 0 | 0 |
| 954 | InChI=1S/C4H6O2/c1-2-3-4(5)6/h2H,1,3H2,(H,5,6) | 0 | 1 | 0 | 0 | 0 | 0 | 0 |
| 955 | InChI=1S/C4H6O2/c1-2-3-4(5)6/h2-3H,1H3,(H,5,6) | 0 | 1 | 0 | 0 | 0 | 0 | 0 |
| 956 | InChI=1S/C4H6O3/c1-3(5)2-4(6)7/h2,5H,1H3,(H,6,7) | 0 | 1 | 0 | 0 | 0 | 0 | 0 |
| 957 | InChI=1S/C4H6O3/c1-2-3(5)4(6)7/h2-3,5H,1H2,(H,6,7) | 0 | 1 | 0 | 0 | 0 | 0 | 0 |
| 958 | InChI=1S/C4H6O3/c1-2-3(5)4(6)7/h2H2,1H3,(H,6,7) | 0 | 1 | 0 | 0 | 0 | 0 | 0 |
| 959 | InChI=1S/C4H6O3/c5-3-1-2-4(6)7/h1-2,5H,3H2,(H,6,7) | 0 | 1 | 0 | 0 | 0 | 0 | 0 |
| 960 | InChI=1S/C4H6O3/c1-3(2-5)4(6)7/h2-3H,1H3,(H,6,7) | 0 | 1 | 0 | 0 | 0 | 0 | 0 |
| 961 | InChI=1S/C4H6O3/c5-3-1-2-4(6)7/h3H,1-2H2,(H,6,7) | 0 | 1 | 0 | 0 | 0 | 0 | 0 |
| 962 | InChI=1S/C4H6O3/c1-3(5)2-4(6)7/h5H,1-2H2,(H,6,7) | 0 | 1 | 0 | 0 | 0 | 0 | 0 |
| 963 | InChI=1S/C4H6O4/c1-2(5)3(6)4(7)8/h5-6H,1H3,(H,7,8) | 0 | 1 | 0 | 0 | 0 | 0 | 0 |
| 964 | InChI=1S/C4H6O4/c5-3(6)1-2-4(7)8/h1-3,5-6H,(H,7,8) | 0 | 1 | 0 | 0 | 0 | 0 | 0 |
| 965 | InChI=1S/C4H6O5/c1-2(5)4(8,9)3(6)7/h8-9H,1H3,(H,6,7) | 0 | 1 | 0 | 0 | 0 | 0 | 0 |
| 966 | InChI=1S/C4H6O5/c5-2(4(8)9)1-3(6)7/h1-2,5-7H,(H,8,9) | 0 | 1 | 0 | 0 | 0 | 0 | 0 |
| 967 | InChI=1S/C4H8O/c1-2-3-4-5/h2,5H,1,3-4H2 | 0 | 1 | 0 | 0 | 0 | 0 | 0 |
| 968 | InChI=1S/C4H8O/c1-2-3-4-5/h2-3,5H,4H2,1H3 | 0 | 1 | 0 | 0 | 0 | 0 | 0 |
| 969 | InChI=1S/C4H8O/c1-2-3-4-5/h3-5H,2H2,1H3 | 0 | 1 | 0 | 0 | 0 | 0 | 0 |
| 970 | InChI=1S/C4H8O2/c1-2-4(6)3-5/h2,4-6H,1,3H2 | 0 | 1 | 0 | 0 | 0 | 0 | 0 |
| 971 | InChI=1S/C4H8O2/c1-3(2)4(5)6/h3H,1-2H3,(H,5,6) | 0 | 1 | 0 | 0 | 0 | 0 | 0 |
| 972 | InChI=1S/C4H8O2/c1-2-3-4(5)6/h2-6H,1H3 | 0 | 1 | 0 | 0 | 0 | 0 | 0 |
| 973 | InChI=1S/C4H8O2/c1-2-3-4(5)6/h2-3H2,1H3,(H,5,6) | 0 | 1 | 0 | 0 | 0 | 0 | 0 |
| 974 | InChI=1S/C4H8O2/c5-3-1-2-4-6/h1,3,5-6H,2,4H2 | 0 | 1 | 0 | 0 | 0 | 0 | 0 |
| 975 | InChI=1S/C4H8O2/c1-3(2)4(5)6/h4-6H,1H2,2H3 | 0 | 1 | 0 | 0 | 0 | 0 | 0 |
| 976 | InChI=1S/C4H8O3/c1-3(5)2-4(6)7/h3,5H,2H2,1H3,(H,6,7) | 0 | 1 | 0 | 0 | 0 | 0 | 0 |
| 977 | InChI=1S/C4H8O3/c1-2-3(5)4(6)7/h3,5H,2H2,1H3,(H,6,7) | 0 | 1 | 0 | 0 | 0 | 0 | 0 |
| 978 | InChI=1S/C4H8O3/c5-3-1-2-4(6)7/h5H,1-3H2,(H,6,7) | 0 | 1 | 0 | 0 | 0 | 0 | 0 |
| 979 | InChI=1S/C4H8O3/c1-3(2-5)4(6)7/h3,5H,2H2,1H3,(H,6,7) | 0 | 1 | 0 | 0 | 0 | 0 | 0 |
| 980 | InChI=1S/C4H8O3/c5-3-1-2-4(6)7/h3-4,6-7H,1-2H2 | 0 | 1 | 0 | 0 | 0 | 0 | 0 |
| 981 | InChI=1S/C4H8O3/c1-4(2,7)3(5)6/h7H,1-2H3,(H,5,6) | 0 | 1 | 0 | 0 | 0 | 0 | 0 |
| 982 | InChI=1S/C4H8O4/c1-2(5)3(6)4(7)8/h2-3,5-6H,1H3,(H,7,8) | 0 | 1 | 0 | 0 | 0 | 0 | 0 |
| 983 | InChI=1S/C4H8O4/c1-2(3(5)6)4(7)8/h2-3,5-6H,1H3,(H,7,8) | 0 | 1 | 0 | 0 | 0 | 0 | 0 |
| 984 | InChI=1S/C4H8O4/c5-3(6)1-2-4(7)8/h1-8H | 0 | 1 | 0 | 0 | 0 | 0 | 0 |
| 985 | InChI=1S/C4H8O4/c5-3(6)1-2-4(7)8/h3,5-6H,1-2H2,(H,7,8) | 0 | 1 | 0 | 0 | 0 | 0 | 0 |
| 986 | InChI=1S/C4H8O4/c5-2-1-3(6)4(7)8/h3,5-6H,1-2H2,(H,7,8) | 0 | 1 | 0 | 0 | 0 | 0 | 0 |
| 987 | InChI=1S/C4H8O4/c5-2-3(6)1-4(7)8/h3,5-6H,1-2H2,(H,7,8) | 0 | 1 | 0 | 0 | 0 | 0 | 0 |
| 988 | InChI=1S/C4H8O5/c1-2(5)4(8,9)3(6)7/h2,5,8-9H,1H3,(H,6,7) | 0 | 1 | 0 | 0 | 0 | 0 | 0 |
| 989 | InChI=1S/C4H8O6/c5-1(3(7)8)2(6)4(9)10/h1-3,5-8H,(H,9,10) | 0 | 1 | 0 | 0 | 0 | 0 | 0 |
| 990 | InChI=1S/C4H10O/c1-2-3-4-5/h5H,2-4H2,1H3 | 0 | 1 | 0 | 0 | 0 | 0 | 0 |
| 991 | InChI=1S/C4H10O2/c5-3-1-2-4-6/h5-6H,1-4H2 | 0 | 1 | 0 | 0 | 0 | 0 | 0 |
| 992 | InChI=1S/C4H10O2/c1-4(6)2-3-5/h4-6H,2-3H2,1H3 | 0 | 1 | 0 | 0 | 0 | 0 | 0 |
| 993 | InChI=1S/C4H10O2/c1-2-4(6)3-5/h4-6H,2-3H2,1H3 | 0 | 1 | 0 | 0 | 0 | 0 | 0 |
| 994 | InChI=1S/C4H10O2/c1-2-3-4(5)6/h4-6H,2-3H2,1H3 | 0 | 1 | 0 | 0 | 0 | 0 | 0 |
| 995 | InChI=1S/C4H10O3/c1-3(5)2-4(6)7/h3-7H,2H2,1H3 | 0 | 1 | 0 | 0 | 0 | 0 | 0 |
| 996 | InChI=1S/C4H10O3/c5-3-1-2-4(6)7/h4-7H,1-3H2 | 0 | 1 | 0 | 0 | 0 | 0 | 0 |
| 997 | InChI=1S/C4H10O3/c5-2-1-4(7)3-6/h4-7H,1-3H2 | 0 | 1 | 0 | 0 | 0 | 0 | 0 |
| 998 | InChI=1S/C4H10O3/c1-3(6)4(7)2-5/h3-7H,2H2,1H3 | 0 | 1 | 0 | 0 | 0 | 0 | 0 |
| 999 | InChI=1S/C4H10O4/c5-3(6)1-2-4(7)8/h3-8H,1-2H2 | 0 | 1 | 0 | 0 | 0 | 0 | 0 |
| 1000 | InChI=1S/C4H10O6/c5-1(3(7)8)2(6)4(9)10/h1-10H | 0 | 1 | 0 | 0 | 0 | 0 | 0 |
| 1001 | InChI=1S/C5H6O2/c1-2-3-4-5(6)7/h2-4H,1H2,(H,6,7) | 0 | 1 | 0 | 0 | 0 | 0 | 0 |
| 1002 | InChI=1S/C5H6O3/c1-3(2)4(6)5(7)8/h1H2,2H3,(H,7,8) | 0 | 1 | 0 | 0 | 0 | 0 | 0 |
| 1003 | InChI=1S/C5H6O3/c1-2-3-4(6)5(7)8/h2H,1,3H2,(H,7,8) | 0 | 1 | 0 | 0 | 0 | 0 | 0 |
| 1004 | InChI=1S/C5H6O3/c1-2-3-4(6)5(7)8/h2-3H,1H3,(H,7,8) | 0 | 1 | 0 | 0 | 0 | 0 | 0 |
| 1005 | InChI=1S/C5H6O4/c1-2-3(4(6)7)5(8)9/h2H,1H3,(H,6,7)(H,8,9) | 0 | 1 | 0 | 0 | 0 | 0 | 0 |
| 1006 | InChI=1S/C5H6O4/c1-3(5(8)9)2-4(6)7/h2H,1H3,(H,6,7)(H,8,9) | 0 | 1 | 0 | 0 | 0 | 0 | 0 |
| 1007 | InChI=1S/C5H6O4/c6-4(7)2-1-3-5(8)9/h1-2H,3H2,(H,6,7)(H,8,9) | 0 | 1 | 0 | 0 | 0 | 0 | 0 |
| 1008 | InChI=1S/C5H6O4/c6-3-1-2-4(7)5(8)9/h3H,1-2H2,(H,8,9) | 0 | 1 | 0 | 0 | 0 | 0 | 0 |
| 1009 | InChI=1S/C5H6O4/c1-3(5(8)9)2-4(6)7/h1-2H2,(H,6,7)(H,8,9) | 0 | 1 | 0 | 0 | 0 | 0 | 0 |
| 1010 | InChI=1S/C5H8O2/c1-2-3-4-5(6)7/h3-4H,2H2,1H3,(H,6,7) | 0 | 1 | 0 | 0 | 0 | 0 | 0 |
| 1011 | InChI=1S/C5H8O2/c1-2-3-4-5(6)7/h2-7H,1H2 | 0 | 1 | 0 | 0 | 0 | 0 | 0 |
| 1012 | InChI=1S/C5H8O2/c1-2-3-4-5(6)7/h2H,1,3-4H2,(H,6,7) | 0 | 1 | 0 | 0 | 0 | 0 | 0 |
| 1013 | InChI=1S/C5H8O3/c1-2-3-4(6)5(7)8/h2,4,6H,1,3H2,(H,7,8) | 0 | 1 | 0 | 0 | 0 | 0 | 0 |
| 1014 | InChI=1S/C5H8O3/c1-2-3-4(6)5(7)8/h2-4,6H,1H3,(H,7,8) | 0 | 1 | 0 | 0 | 0 | 0 | 0 |
| 1015 | InChI=1S/C5H8O3/c1-2-3-4(6)5(7)8/h2-3H2,1H3,(H,7,8) | 0 | 1 | 0 | 0 | 0 | 0 | 0 |
| 1016 | InChI=1S/C5H8O3/c1-3(2)4(6)5(7)8/h4,6H,1H2,2H3,(H,7,8) | 0 | 1 | 0 | 0 | 0 | 0 | 0 |
| 1017 | InChI=1S/C5H8O3/c1-3(2)4(6)5(7)8/h3H,1-2H3,(H,7,8) | 0 | 1 | 0 | 0 | 0 | 0 | 0 |
| 1018 | InChI=1S/C5H8O3/c1-2-4(6)3-5(7)8/h2,4,6H,1,3H2,(H,7,8) | 0 | 1 | 0 | 0 | 0 | 0 | 0 |
| 1019 | InChI=1S/C5H8O4/c1-3(5(8)9)2-4(6)7/h3H,2H2,1H3,(H,6,7)(H,8,9) | 0 | 1 | 0 | 0 | 0 | 0 | 0 |
| 1020 | InChI=1S/C5H8O4/c6-4(7)2-1-3-5(8)9/h1-3H2,(H,6,7)(H,8,9) | 0 | 1 | 0 | 0 | 0 | 0 | 0 |
| 1021 | InChI=1S/C5H8O4/c6-3-4(7)1-2-5(8)9/h1-2,4,6-7H,3H2,(H,8,9) | 0 | 1 | 0 | 0 | 0 | 0 | 0 |
| 1022 | InChI=1S/C5H8O4/c1-5(2,3(6)7)4(8)9/h1-2H3,(H,6,7)(H,8,9) | 0 | 1 | 0 | 0 | 0 | 0 | 0 |
| 1023 | InChI=1S/C5H8O4/c1-3(6)2-4(7)5(8)9/h3,6H,2H2,1H3,(H,8,9) | 0 | 1 | 0 | 0 | 0 | 0 | 0 |
| 1024 | InChI=1S/C5H8O4/c1-2-3(4(6)7)5(8)9/h3H,2H2,1H3,(H,6,7)(H,8,9) | 0 | 1 | 0 | 0 | 0 | 0 | 0 |
| 1025 | InChI=1S/C5H8O4/c1-3(5(8)9)2-4(6)7/h2,4,6-7H,1H3,(H,8,9) | 0 | 1 | 0 | 0 | 0 | 0 | 0 |
| 1026 | InChI=1S/C5H8O4/c1-5(2,9)3(6)4(7)8/h9H,1-2H3,(H,7,8) | 0 | 1 | 0 | 0 | 0 | 0 | 0 |
| 1027 | InChI=1S/C5H8O5/c1-5(10,4(8)9)2-3(6)7/h10H,2H2,1H3,(H,6,7)(H,8,9) | 0 | 1 | 0 | 0 | 0 | 0 | 0 |
| 1028 | InChI=1S/C5H8O5/c1-2-5(10,3(6)7)4(8)9/h10H,2H2,1H3,(H,6,7)(H,8,9) | 0 | 1 | 0 | 0 | 0 | 0 | 0 |
| 1029 | InChI=1S/C5H8O5/c1-2(4(7)8)3(6)5(9)10/h2-3,6H,1H3,(H,7,8)(H,9,10) | 0 | 1 | 0 | 0 | 0 | 0 | 0 |
| 1030 | InChI=1S/C5H8O5/c6-3(5(9)10)1-2-4(7)8/h3,6H,1-2H2,(H,7,8)(H,9,10) | 0 | 1 | 0 | 0 | 0 | 0 | 0 |
| 1031 | InChI=1S/C5H8O5/c6-3(1-4(7)8)2-5(9)10/h3,6H,1-2H2,(H,7,8)(H,9,10) | 0 | 1 | 0 | 0 | 0 | 0 | 0 |
| 1032 | InChI=1S/C5H8O5/c6-2-3(7)1-4(8)5(9)10/h3,6-7H,1-2H2,(H,9,10) | 0 | 1 | 0 | 0 | 0 | 0 | 0 |
| 1033 | InChI=1S/C5H10O3/c1-2-3-4(6)5(7)8/h4,6H,2-3H2,1H3,(H,7,8) | 0 | 1 | 0 | 0 | 0 | 0 | 0 |
| 1034 | InChI=1S/C5H10O4/c6-3-1-2-4(7)5(8)9/h4,6-7H,1-3H2,(H,8,9) | 0 | 1 | 0 | 0 | 0 | 0 | 0 |
| 1035 | InChI=1S/C5H10O4/c1-2-3(6)4(7)5(8)9/h3-4,6-7H,2H2,1H3,(H,8,9) | 0 | 1 | 0 | 0 | 0 | 0 | 0 |
| 1036 | InChI=1S/C5H10O5/c6-2-3(7)1-4(8)5(9)10/h3-4,6-8H,1-2H2,(H,9,10) | 0 | 1 | 0 | 0 | 0 | 0 | 0 |
| 1037 | InChI=1S/C6H6O5/c7-4(6(10)11)2-1-3-5(8)9/h1-2H,3H2,(H,8,9)(H,10,11) | 0 | 1 | 0 | 0 | 0 | 0 | 0 |
| 1038 | InChI=1S/C6H6O5/c7-4(6(10)11)2-1-3-5(8)9/h1,3H,2H2,(H,8,9)(H,10,11) | 0 | 1 | 0 | 0 | 0 | 0 | 0 |
| 1039 | InChI=1S/C6H6O5/c1-3(5(8)9)2-4(7)6(10)11/h1-2H2,(H,8,9)(H,10,11) | 0 | 1 | 0 | 0 | 0 | 0 | 0 |
| 1040 | InChI=1S/C6H8O5/c7-4(6(10)11)2-1-3-5(8)9/h1-3H2,(H,8,9)(H,10,11) | 0 | 1 | 0 | 0 | 0 | 0 | 0 |
| 1041 | InChI=1S/C6H8O5/c1-3(5(8)9)2-4(7)6(10)11/h3H,2H2,1H3,(H,8,9)(H,10,11) | 0 | 1 | 0 | 0 | 0 | 0 | 0 |
| 1042 | InChI=1S/C6H10O5/c7-4(6(10)11)2-1-3-5(8)9/h4,7H,1-3H2,(H,8,9)(H,10,11) | 0 | 1 | 0 | 0 | 0 | 0 | 0 |
| 1043 | InChI=1S/C6H10O6/c1-6(12,5(10)11)2-3(7)4(8)9/h3,7,12H,2H2,1H3,(H,8,9)(H,10,11) | 0 | 1 | 0 | 0 | 0 | 0 | 0 |
| 1044 | InChI=1S/C6H10O7/c7-3(8)1-6(13,5(11)12)2-4(9)10/h5,11-13H,1-2H2,(H,7,8)(H,9,10) | 0 | 1 | 0 | 0 | 0 | 0 | 0 |
| 1045 | InChI=1S/C6H10O7/c7-3(8)1-6(13,5(11)12)2-4(9)10/h3,7-8,13H,1-2H2,(H,9,10)(H,11,12) | 0 | 1 | 0 | 0 | 0 | 0 | 0 |
| 1046 | InChI=1S/C7H6O7/c8-4(7(13)14)1-3(6(11)12)2-5(9)10/h2H,1H2,(H,9,10)(H,11,12)(H,13,14) | 0 | 1 | 0 | 0 | 0 | 0 | 0 |
| 1047 | InChI=1S/C7H6O7/c8-4(7(13)14)1-3(6(11)12)2-5(9)10/h1H,2H2,(H,9,10)(H,11,12)(H,13,14) | 0 | 1 | 0 | 0 | 0 | 0 | 0 |
| 1048 | InChI=1S/C7H8O6/c8-4(6(10)11)2-1-3-5(9)7(12)13/h1-3H2,(H,10,11)(H,12,13) | 0 | 1 | 0 | 0 | 0 | 0 | 0 |
| 1049 | InChI=1S/C7H8O7/c8-4(9)2-1-3(6(11)12)5(10)7(13)14/h3H,1-2H2,(H,8,9)(H,11,12)(H,13,14) | 0 | 1 | 0 | 0 | 0 | 0 | 0 |
| 1050 | InChI=1S/C7H8O7/c8-4(7(13)14)1-3(6(11)12)2-5(9)10/h3H,1-2H2,(H,9,10)(H,11,12)(H,13,14) | 0 | 1 | 0 | 0 | 0 | 0 | 0 |
| 1051 | InChI=1S/C7H8O8/c8-3(9)1-2(5(10)11)4(6(12)13)7(14)15/h2,4H,1H2,(H,8,9)(H,10,11)(H,12,13)(H,14,15) | 0 | 1 | 0 | 0 | 0 | 0 | 0 |
| 1052 | InChI=1S/C7H8O8/c8-3(5(11)12)1-7(15,6(13)14)2-4(9)10/h15H,1-2H2,(H,9,10)(H,11,12)(H,13,14) | 0 | 1 | 0 | 0 | 0 | 0 | 0 |

**Table SI1**: Correspondence of the compounds found in Morowitz *et al.* ^1^, Zubarev *et al.* ^2^, chemical databases (eMolecules, PubChem and Reaxys, respectively), the rTCA cycle and the present study (MOLGEN) represented by InChi. Rows 1-876 represent structures occurring in our rTCA chemical space. Rows 877-910 show structures additionally present in Morowitz *et al.* ^1^, and rows 911-1052 further structures appearing only in the set of Zubarev *et al.* ^2^. For the latter two groups no database queries were executed and the corresponding fields are left blank. This table is also provided as tab-separated text file named TableSI1.txt.

|  | Formula | Morowitz | Zubarev | MOLGEN | eMolecules | PubChem | Reaxys | rTCA |
| --- | --- | --- | --- | --- | --- | --- | --- | --- |
| 1 | CH_2_O | 1 | 1 | 1 | 1 | 1 | 1 | 0 |
| 2 | CH_2_O_2_ | 1 | 1 | 1 | 1 | 1 | 1 | 0 |
| 3 | CH_2_O_3_ | 0 | 0 | 1 | 1 | 1 | 1 | 0 |
| 4 | C_2_H_2_O_2_ | 1 | 1 | 1 | 1 | 1 | 1 | 0 |
| 5 | C_2_H_2_O_3_ | 1 | 1 | 1 | 1 | 1 | 1 | 0 |
| 6 | C_2_H_2_O_4_ | 1 | 1 | 1 | 1 | 1 | 1 | 0 |
| 7 | C_2_H_4_O_2_ | 2 | 3 | 2 | 2 | 2 | 2 | 1 |
| 8 | C_2_H_4_O_3_ | 2 | 3 | 1 | 1 | 1 | 1 | 0 |
| 9 | C_3_H_2_O_3_ | 1 | 0 | 1 | 0 | 1 | 1 | 0 |
| 10 | C_3_H_2_O_4_ | 1 | 1 | 1 | 0 | 1 | 1 | 0 |
| 11 | C_3_H_2_O_5_ | 1 | 1 | 1 | 1 | 1 | 1 | 0 |
| 12 | C_3_H_4_O_3_ | 7 | 4 | 4 | 3 | 4 | 4 | 1 |
| 13 | C_3_H_4_O_4_ | 6 | 3 | 3 | 1 | 3 | 3 | 0 |
| 14 | C_3_H_4_O_5_ | 1 | 2 | 1 | 1 | 1 | 1 | 0 |
| 15 | C_3_H_6_O_3_ | 5 | 3 | 4 | 4 | 4 | 4 | 0 |
| 16 | C_3_H_6_O_4_ | 2 | 3 | 1 | 1 | 1 | 1 | 0 |
| 17 | C_4_H_2_O_4_ | 2 | 0 | 1 | 0 | 1 | 1 | 0 |
| 18 | C_4_H_2_O_5_ | 0 | 0 | 1 | 0 | 0 | 0 | 0 |
| 19 | C_4_H_2_O_6_ | 1 | 1 | 1 | 1 | 1 | 1 | 0 |
| 20 | C_4_H_4_O_4_ | 6 | 4 | 9 | 1 | 8 | 5 | 1 |
| 21 | C_4_H_4_O_5_ | 2 | 3 | 6 | 1 | 2 | 2 | 1 |
| 22 | C_4_H_4_O_6_ | 2 | 2 | 3 | 0 | 2 | 1 | 0 |
| 23 | C_4_H_4_O_7_ | 1 | 1 | 1 | 0 | 1 | 1 | 0 |
| 24 | C_4_H_6_O_4_ | 11 | 9 | 15 | 2 | 14 | 10 | 1 |
| 25 | C_4_H_6_O_5_ | 6 | 5 | 7 | 1 | 7 | 5 | 1 |
| 26 | C_4_H_6_O_6_ | 3 | 2 | 2 | 1 | 2 | 2 | 0 |
| 27 | C_5_H_2_O_5_ | 1 | 0 | 1 | 0 | 0 | 0 | 0 |
| 28 | C_5_H_2_O_6_ | 0 | 0 | 1 | 0 | 0 | 0 | 0 |
| 29 | C_5_H_2_O_7_ | 0 | 0 | 1 | 0 | 0 | 0 | 0 |
| 30 | C_5_H_4_O_5_ | 2 | 2 | 15 | 0 | 2 | 2 | 0 |
| 31 | C_5_H_4_O_6_ | 1 | 1 | 12 | 0 | 1 | 1 | 0 |
| 32 | C_5_H_4_O_7_ | 4 | 0 | 6 | 0 | 2 | 2 | 0 |
| 33 | C_5_H_4_O_8_ | 1 | 0 | 2 | 0 | 1 | 1 | 0 |
| 34 | C_5_H_6_O_5_ | 5 | 3 | 44 | 2 | 18 | 7 | 1 |
| 35 | C_5_H_6_O_6_ | 5 | 3 | 25 | 0 | 8 | 5 | 0 |
| 36 | C_5_H_6_O_7_ | 4 | 2 | 9 | 0 | 6 | 4 | 0 |
| 37 | C_5_H_6_O_8_ | 1 | 0 | 1 | 0 | 1 | 1 | 0 |
| 38 | C_5_H_8_O_6_ | 10 | 3 | 18 | 1 | 12 | 9 | 0 |
| 39 | C_5_H_8_O_7_ | 2 | 0 | 3 | 1 | 3 | 2 | 0 |
| 40 | C_6_H_2_O_6_ | 0 | 0 | 1 | 0 | 0 | 0 | 0 |
| 41 | C_6_H_2_O_7_ | 0 | 0 | 1 | 0 | 0 | 0 | 0 |
| 42 | C_6_H_2_O_8_ | 0 | 0 | 1 | 0 | 0 | 0 | 0 |
| 43 | C_6_H_4_O_6_ | 1 | 0 | 27 | 0 | 1 | 1 | 0 |
| 44 | C_6_H_4_O_7_ | 0 | 0 | 20 | 0 | 0 | 0 | 0 |
| 45 | C_6_H_4_O_8_ | 1 | 0 | 11 | 0 | 1 | 1 | 0 |
| 46 | C_6_H_4_O_9_ | 0 | 0 | 3 | 0 | 0 | 0 | 0 |
| 47 | C_6_H_6_O_6_ | 7 | 3 | 122 | 1 | 16 | 7 | 1 |
| 48 | C_6_H_6_O_7_ | 2 | 2 | 73 | 0 | 6 | 1 | 1 |
| 49 | C_6_H_6_O_8_ | 3 | 4 | 31 | 1 | 4 | 3 | 0 |
| 50 | C_6_H_6_O_9_ | 0 | 0 | 6 | 0 | 1 | 0 | 0 |
| 51 | C_6_H_6_O10 | 0 | 0 | 1 | 0 | 0 | 0 | 0 |
| 52 | C_6_H_8_O_6_ | 6 | 5 | 198 | 1 | 39 | 12 | 0 |
| 53 | C_6_H_8_O_7_ | 7 | 2 | 93 | 2 | 16 | 8 | 2 |
| 54 | C_6_H_8_O_8_ | 3 | 1 | 25 | 0 | 10 | 4 | 0 |
| 55 | C_6_H_8_O_9_ | 1 | 0 | 3 | 0 | 3 | 1 | 0 |
| 56 | C_6_H10O_7_ | 12 | 2 | 44 | 3 | 17 | 12 | 0 |
| 57 | C_6_H10O_8_ | 3 | 0 | 7 | 1 | 3 | 2 | 0 |
| 58 | C_2_H_4_O_4_ | 1 | 1 | 0 | 0 | 0 | 0 | 0 |
| 59 | C_3_H_4_O_6_ | 1 | 1 | 0 | 0 | 0 | 0 | 0 |
| 60 | C_4_H_6_O_8_ | 1 | 0 | 0 | 0 | 0 | 0 | 0 |
| 61 | C_6_H10O10 | 1 | 0 | 0 | 0 | 0 | 0 | 0 |
| 62 | CH_4_O | 0 | 1 | 0 | 0 | 0 | 0 | 0 |
| 63 | CH_4_O_2_ | 0 | 1 | 0 | 0 | 0 | 0 | 0 |
| 64 | C_2_H_4_ | 0 | 1 | 0 | 0 | 0 | 0 | 0 |
| 65 | C_2_H_4_O | 0 | 2 | 0 | 0 | 0 | 0 | 0 |
| 66 | C_2_H_6_ | 0 | 1 | 0 | 0 | 0 | 0 | 0 |
| 67 | C_2_H_6_O | 0 | 1 | 0 | 0 | 0 | 0 | 0 |
| 68 | C_2_H_6_O_2_ | 0 | 2 | 0 | 0 | 0 | 0 | 0 |
| 69 | C_2_H_6_O_3_ | 0 | 2 | 0 | 0 | 0 | 0 | 0 |
| 70 | C_2_H_6_O_4_ | 0 | 1 | 0 | 0 | 0 | 0 | 0 |
| 71 | C_3_H_4_O_2_ | 0 | 1 | 0 | 0 | 0 | 0 | 0 |
| 72 | C_3_H_6_ | 0 | 1 | 0 | 0 | 0 | 0 | 0 |
| 73 | C_3_H_6_O | 0 | 3 | 0 | 0 | 0 | 0 | 0 |
| 74 | C_3_H_6_O_2_ | 0 | 4 | 0 | 0 | 0 | 0 | 0 |
| 75 | C_3_H_8_ | 0 | 1 | 0 | 0 | 0 | 0 | 0 |
| 76 | C_3_H_8_O | 0 | 2 | 0 | 0 | 0 | 0 | 0 |
| 77 | C_3_H_8_O_2_ | 0 | 4 | 0 | 0 | 0 | 0 | 0 |
| 78 | C_3_H_8_O_3_ | 0 | 3 | 0 | 0 | 0 | 0 | 0 |
| 79 | C_3_H_8_O_4_ | 0 | 2 | 0 | 0 | 0 | 0 | 0 |
| 80 | C_4_H_4_O_3_ | 0 | 2 | 0 | 0 | 0 | 0 | 0 |
| 81 | C_4_H_6_ | 0 | 1 | 0 | 0 | 0 | 0 | 0 |
| 82 | C_4_H_6_O | 0 | 1 | 0 | 0 | 0 | 0 | 0 |
| 83 | C_4_H_6_O_2_ | 0 | 3 | 0 | 0 | 0 | 0 | 0 |
| 84 | C_4_H_6_O_3_ | 0 | 7 | 0 | 0 | 0 | 0 | 0 |
| 85 | C_4_H_8_O | 0 | 3 | 0 | 0 | 0 | 0 | 0 |
| 86 | C_4_H_8_O_2_ | 0 | 6 | 0 | 0 | 0 | 0 | 0 |
| 87 | C_4_H_8_O_3_ | 0 | 6 | 0 | 0 | 0 | 0 | 0 |
| 88 | C_4_H_8_O_4_ | 0 | 6 | 0 | 0 | 0 | 0 | 0 |
| 89 | C_4_H_8_O_5_ | 0 | 1 | 0 | 0 | 0 | 0 | 0 |
| 90 | C_4_H_8_O_6_ | 0 | 1 | 0 | 0 | 0 | 0 | 0 |
| 91 | C_4_H10O | 0 | 1 | 0 | 0 | 0 | 0 | 0 |
| 92 | C_4_H10O_2_ | 0 | 4 | 0 | 0 | 0 | 0 | 0 |
| 93 | C_4_H10O_3_ | 0 | 4 | 0 | 0 | 0 | 0 | 0 |
| 94 | C_4_H10O_4_ | 0 | 1 | 0 | 0 | 0 | 0 | 0 |
| 95 | C_4_H10O_6_ | 0 | 1 | 0 | 0 | 0 | 0 | 0 |
| 96 | C_5_H_6_O_2_ | 0 | 1 | 0 | 0 | 0 | 0 | 0 |
| 97 | C_5_H_6_O_3_ | 0 | 3 | 0 | 0 | 0 | 0 | 0 |
| 98 | C_5_H_6_O_4_ | 0 | 5 | 0 | 0 | 0 | 0 | 0 |
| 99 | C_5_H_8_O_2_ | 0 | 3 | 0 | 0 | 0 | 0 | 0 |
| 100 | C_5_H_8_O_3_ | 0 | 6 | 0 | 0 | 0 | 0 | 0 |
| 101 | C_5_H_8_O_4_ | 0 | 8 | 0 | 0 | 0 | 0 | 0 |
| 102 | C_5_H_8_O_5_ | 0 | 6 | 0 | 0 | 0 | 0 | 0 |
| 103 | C_5_H10O_3_ | 0 | 1 | 0 | 0 | 0 | 0 | 0 |
| 104 | C_5_H10O_4_ | 0 | 2 | 0 | 0 | 0 | 0 | 0 |
| 105 | C_5_H10O_5_ | 0 | 1 | 0 | 0 | 0 | 0 | 0 |
| 106 | C_6_H_6_O_5_ | 0 | 3 | 0 | 0 | 0 | 0 | 0 |
| 107 | C_6_H_8_O_5_ | 0 | 2 | 0 | 0 | 0 | 0 | 0 |
| 108 | C_6_H10O_5_ | 0 | 1 | 0 | 0 | 0 | 0 | 0 |
| 109 | C_6_H10O_6_ | 0 | 1 | 0 | 0 | 0 | 0 | 0 |
| 110 | C_7_H_6_O_7_ | 0 | 2 | 0 | 0 | 0 | 0 | 0 |
| 111 | C_7_H_8_O_6_ | 0 | 1 | 0 | 0 | 0 | 0 | 0 |
| 112 | C_7_H_8_O_7_ | 0 | 2 | 0 | 0 | 0 | 0 | 0 |
| 113 | C_7_H_8_O_8_ | 0 | 2 | 0 | 0 | 0 | 0 | 0 |

**Table SI2.** Summary of the formulas found in Morowitz *et al.* ^1^, Zubarev *et al.* ^2^, chemical databases (eMolecules, PubChem and Reaxys, respectively), the rTCA cycle and the present study (MOLGEN). Rows 1-57 represent formulas occurring in our rTCA chemical space. Rows 58-61 show formulas additionally present in Morowitz *et al.* ^1^, and rows 62-113 further formulas appearing only in the set of Zubarev *et al.* ^2^. For the latter two groups no database queries were executed and the corresponding fields are left blank.

**Commands used for operating MOLGEN 5**

These are the two commands used for structure generation with MOLGEN, executed in the Windows PowerShell:

Measure-Command{C:/Programs/Molgen5.0/mgen.exe C1-3H2-99O1-7 -sum O-C=0-99
-sum 2O-H=0-99 -substr open 1-99 ../mol/C=O.mol
–badlist ../sdf/BadListTCA.sdf -cycles 0 -maxbond 2 -v
-o MorowitzC1-3.sdf 2> MorowitzC1-3.number.txt} > MorowitzC1-3.time.txt

Measure-Command{C:/Programs/Molgen5.0/mgen.exe C4-6H2-99O1-13 -sum O-C=0-99
-sum 3O-2H=0-99 -substr open 1-99 ../mol/C=O.mol
-badlist ../sdf/BadMorowitzList.sdf -cycles 0 -maxbond 2 -v
-o MorowitzC4-6.sdf 2> MorowitzC4-6.number.txt} > MorowitzC4-6.time.txt

We split the generation of our set into two parts: one for 1-3 C atoms where the ratio of numbers of H and O atoms n(H)/n(O) ≤ 2 and a second part for 4-6 C atoms where n(H)/n(O) ≤ 1.5.

MeasureCommand is a PowerShell command to measure the time required for the execution of a program. Its output is redirected to MorowitzC1-3.time.txt and MorowitzC4-6.time.txt. C:/Programs/Molgen5.0/mgen.exe is the path to the MOLGEN 5 executable.

C1-3H2-99O1-7 and C4-6H2-99O1-13 are fuzzy molecular formulas used for the two program calls. The upper bound of 99 for the number of H atoms is just an arbitrary setting to a sufficiently high number. MOLGEN finds tighter bounds itself. The command line arguments -sum O-C=0-99 represent the rule n(C)/n(O) ≤ 1, which is equivalent to n(O)-n(C) ≥ 0. Literally the arguments -sum O-C=0-99 mean that the number of O atoms minus the number of C atoms must be within the interval [0, 99] where 99 is an arbitrary upper limit. In a similar way, arguments -sum 2O-H=0-99 and -sum 3O-2H=0-99 encode the other constraints on the molecular formulas, n(H)/n(O) ≤ 2 and n(H)/n(O) ≤ 1.5. Arguments -substr open 1-99 ../mol/C=O.mol prescribe the presence of a C=O (carbonyl) substructure, where C=O is encoded in a molfile named C=O.mol. Arguments -badlist ../sdf/BadListTCA.sdf define an SDfile of six forbidden substructures. These substructures are depicted in Figure SI1. The molfile C=O.mol and the SDfile BadListTCA.sdf are also part of the SI.

The command line parameters -cycles 0 set the number of cycles to 0, *i.e.*, only acyclic structures are generated. Parameters -maxbond 2 set the maximum bond multiplicity to 2, *i.e.*, no triple bonds are allowed. Option -v enables verbosity on the lowest level, *i.e.*, MOLGEN writes a summary of the input and the number of generated structures to the standard output, which is redirected to text files named MorowitzC1-3.number.txt and MorowitzC4-6.number.txt. Parameters -o MorowitzC1-3.sdf and -o MorowitzC4-6.sdf tell the program to write the generated structures to SDfiles named MorowitzC1-3.sdf and MorowitzC1-3.sdf.

The software user manual of MOLGEN 5 is provided here: http://molgen.de/documents/manual_molgen50.pdf

**Figure SI1.** Forbidden substructures used for structure generation: **(1)** and **(2)** are directly taken from Morowitz *et al.* ^1^ **(3)** is adopted from Schuster ^3^. **(4)**, **(5)** and **(6)** were introduced to avoid tautomeric duplicates (hydrates, enols). “A” here represents any heavy atom except H.

**Working principle of MOLGEN 5**

In order to sketch MOLGEN’s working principle we here focus on the features used in the present study. A more comprehensive description can be found in reference 4. Approaching the molecular structure we distinguish several levels of detail:

- *Fuzzy molecular formula*: For each chemical element the minimum and the maximum number of atoms is defined.
- *Exact molecular formula*: For each chemical element the exact number of atoms is defined.
- *Atom state pattern*: For each non-H atom in the molecular formula, its state is fully defined, including the numbers of bonds of various types (single, double, triple, etc.) and the number of H atoms attached to it.
- *Molecular graph*: A chemical structure can be understood as a graph, where nodes correspond to atoms and edges encode covalent bonds between atoms. For this purpose, the nodes are labelled by the chemical identity of the represented atoms along with additional higher order information describing the state of the atom, while edges represent the bond type.

Generation can be started from any of the first three levels and stop at any of the last three levels. In our application we do indeed start from two fuzzy formulas and go down to molecular graphs. As part of the algorithm so-called *induced restrictions* are automatically introduced to the appropriate levels. *E.g.* the prescribed substructure C=O induces at least one C atom with a double bond and at least one O atom with a double bond to the level of atom state patterns. Below we describe in more detail which principles and rules are applied when stepping from the level of fuzzy molecular formulas via exact molecular formulas and atom state patterns to molecular graphs.

- From *fuzzy formula* to *exact formulas*: For a given fuzzy formula the generator runs through all corresponding exact formulas. The implementation is straightforward, via backtracking. Several tests are executed before a molecular formula is written to the output or passed to the next level. They are derived from graph theory and chemistry:
  - The sum of valences must be even.

Let *a* denote the number of atoms including H atoms and *b* be half of the sum of valences, *i.e.*, the sum of all bond multiplicities in any graph corresponding to the formula.

- - Then *b* must be greater than or equal to the maximum valence occurring in the formula,
  - *a* – *b* ≤ *c_max_* must be fulfilled, where c_max_ is the maximal allowed number of connected components (default is 1).

Further, all user–defined restrictions on molecular formulas must be fulfilled, which are in our application the atom sums.

- From *exact molecular formula* to *atom state patterns*: A system of linear equations is established, where the variables are restricted to non-negative integer values. MOLGEN contains its own algorithm called ‘solvediophant’ to solve these systems of equations. It is based on the mathematical concept of lattice basis reduction^5,6^. Let *a_i_*, *t_i_*, *d_i_* be the numbers of aromatic, triple and double bonds incident with non–H atom *i*, *s_i_* its number of single bonds to non–H atoms, and *h_i_* its number of H atoms. Then the number of bonds in the molecule is equal to half of the sum ∑*_i_* (*a_i_* + *t_i_* + *d_i_* + *s_i_* + 2*h_i_*). The following restrictions are formulated as diophantine equations (all sums are over the non–H atoms):
  - The numbers of aromatic, triple, double, single bonds fulfill the corresponding restrictions. In our application *a_i_ = t_i_* = 0.
  - The number of bonds, rings and connected components fulfil their corresponding restrictions. In our application the number of rings is 0 and the number of connected components is 1.
  - The sum ∑*_i_* (*a_i_* + *t_i_* + *d_i_* + *s_i_* + 2*h_i_*) is even (as it is twice the number of bonds).
  - The sums ∑*_i_ a_i_*, ∑*_i_ t_i_*, ∑*_i_* *d_i_*, ∑*_i_* *s_i_* are all even (as they are twice the number of aromatic, triple, double or single bonds between non–H atoms).
  - The sum ∑*_I_* *h_i_* is equal to the number of H atoms in the exact molecular formula.
  - The following equation must be fulfilled: atoms (incl. H) + cycles = bonds + connected components. In our application the number of rings is 0 and the number of connected components is 1.
  - For each non–H atom, the sum of valences resulting from incident bonds and attached H atoms must be consistent with its valence defined for the chemical element. In our application default valences are used: 2 for O, 4 for C.
  - Additional equations ensure that each atom state pattern is produced only once by the diophantic solver. We allow only such atom state patterns in which the list of atom states is sorted in lexicographically decreasing order.
- From *atom state pattern* to *molecular graphs*: The construction of all molecular graphs corresponding to an atom state pattern is done using the principle of orderly generation. It is convenient to use the lexicographical order on the adjacency matrix A as construction sequence. Objects with maximal adjacency matrix are deﬁned to be canonical orbit representatives. Before starting to ﬁll the adjacency matrix, the atom states are assigned to rows (and columns) of A. The assignment of atom states to rows and columns of the adjacency matrix introduces a block structure as depicted below.

A^(1)^

A^(2)^

A^(^*^r^*^)^

A^(^*^t^*^)^

•

•

•

•

•

•

{

{

{

{

A **=**

λ_1_

λ_2_

λ*_r_*

λ*_t_*

Each block A^(^*^r^*^)^ belongs to one of the *t* diﬀerent atom states; λ*_r_* equals the number of atoms of a state *r*. The algorithm sketched below is taken from references 7 and 8 and shows how the structure generator in MOLGEN ﬁlls the adjacency matrix. Filling matrix blocks (steps 3 and 4) is iterated with testing canonicity for matrix blocks (step 5). For canonicity testing of block *r* only permutations from the formerly calculated automorphism group Aut^(^*^r^*^−1)^(A) of blocks 1, ..., *r* − 1 have to be taken into account.

Algorithm: *MOLGEN orderly enumeration*

1. Start: set *r* := 0 and goto (3).
2. Stop criterion: if *r* = 0 stop; else goto (4).
3. Maximum ﬁlling: ﬁll block A^(^*^r^*^)^ (depending on A^(1)^, ..., A^(^*^r^*^−1)^) in lexicographically maximal manner so that A^(^*^r^*^)^ fulﬁlls the desired matrix properties (regarding atom states and further constraints).
   If no such ﬁlling exists then set *r* := *r* − 1 and goto (2); else goto (5).
4. Next smaller ﬁlling: ﬁll block A^(^*^r^*^)^ (depending on A^(1)^, ..., A^(^*^r^*^−1)^) in lexicographically next smaller manner so that A (r) fulﬁlls the desired matrix properties (regarding atom states and further constraints).
   If no such ﬁlling exists then set *r* := *r* − 1 and goto (2); else goto (5).
5. Test canonicity: if ∀π ∈ Aut^(^*^r^*^−1)^(A) : A^(^*^r^*^)^ ≥ π(A^(^*^r^*^)^), then

if *r* = *t* (canonical matrix complete) then

- - 1. if constraints are fulﬁlled then output A
    2. goto (4)

else determine Aut^(^*^r^*^)^(A), set *r* := *r* + 1 and goto (3).

else goto (4)

**References**

1 Morowitz, H. J., Kostelnik, J. D., Yang, J. & Cody, G. D. The origin of intermediary metabolism. *Proceedings of the National Academy of Sciences* **97**, 7704-7708 (2000).

2 Zubarev, D. Y., Rappoport, D. & Aspuru-Guzik, A. Uncertainty of prebiotic scenarios: The case of the non-enzymatic reverse tricarboxylic acid cycle. *Scientific Reports* **5** (2015).

3 Schuster, P. Taming combinatorial explosion. *Proceedings of the National Academy of Sciences* **97**, 7678-7680 (2000).

4 Gugisch, R. *et al.* MOLGEN 5.0, A Molecular Structure Generator. In *Advances in Mathematical Chemistry and Applications: Revised Edition* Vol. 1 113-138 (2016).

5 Wassermann, A. Finding simple t-designs with enumeration techniques. *Journal of Combinatorial Design* **6**, 79-90 (1998).

6 Wassermann, A. Attacking the market split problem with lattice point enumeration. *Journal of Combinatorial* *Optimization* **6**, 5–16 (2002).

7 Grund, R. Konstruktion molekularer Graphen mit gegebenen Hybridisierungen und überlappungsfreien Fragmenten. *Bayreuther Mathematische Schriften* **49**, 1–113 (1995).

8 Meringer, M. Structure Enumeration and Sampling. In *Handbook of Chemoinformatics Algorithms.* 233-267 (CRC/Chapmann & Hall, 2010).
